# Improving ovarian cancer treatment decision using a novel risk predictive tool

**DOI:** 10.18632/aging.204023

**Published:** 2022-04-19

**Authors:** Zhenyi Xu, Jiali Song, Lei Cao, Zhiwei Rong, Wenjie Zhang, Jia He, Kang Li, Yan Hou

**Affiliations:** 1 Department of Epidemiology and Biostatistics, School of Public Health, Harbin Medical University, Harbin 150086, China; 2 Department of Biostatistics, Peking University, Beijing 100000, China

**Keywords:** tumor microenvironment, predictive factor, immunotherapy, targeted therapy, gene mutation

## Abstract

Background: As a major component of the tumor tissue, the tumor microenvironment (TME) has been proven to associate with tumor progression and immunotherapy. Ovarian cancer accounts for the highest mortality rate among gynecologic malignancies. Its clinical treatment decision is highly correlated with the prognosis, underscoring the need to evaluate the prognosis and choose the proper clinical treatment through TME information.

Method: This study constructs a score with TME information obtained by the CIBERSORT algorithm, which classifies the patients into high and low TMEscore groups with quantified TME infiltration patterns through the PCA algorithm. TMEscore was constructed by TCGA cohort and validated in GEO cohort. Univariate and multivariate Cox proportional hazards model analyses were used to demonstrate prognostic value of TMEscore in overall and stratified analysis.

Result: TMEscore is highly correlated with survival and high TMEscore group has a better prognosis. In order to improve treatment decision, the expression of immune checkpoints, immunophenoscore (IPS) and ESTIMATE score showed a high TMEscore have a better immune microenvironment and respond better to immune checkpoint inhibitors (ICIs). Meanwhile, the mutation landscape between TMEscore groups was profiled, and 13 genes were found mutated differently between the two groups. Among them, BRCA1 has more mutations in the high TMEscore group and speculated that high TMEscore patients might be a beneficiary population of PARP inhibitors combined with immunotherapy.

Conclusion: TMEscore based on TME with prognostic value and clinical value is proposed for the identification of targets treatment and immunotherapy strategies for ovarian cancer.

## INTRODUCTION

The tumor microenvironment (TME) is created by the tumor and dominated by tumor-induced interactions [1]. Within the TME infrastructure, various types of immune (innate and adaptive immune cells) and non-immune (stromal cells, fibroblasts, endothelial cells) are found. These cells drive a chronic inflammatory, immunosuppressive, and pro-angiogenic intratumoral environment by secreting downstream factors [2, 3]. To date, several studies have found that TME not only plays an essential role in tumor initiation, disease progression, and metastatic development but also has profound effects on therapeutic efficacy [4]. Therefore, identifying effective targeted drugs with TME provides new insights into case-specific medical care. Immune checkpoints such as programmed death (PD-1) and its ligand (PD-1 ligand [PD-L1]) have been found ligand-receptor interactions between cancer cells and host immune cells in TME. The corresponding monoclonal antibody agents like nivolumab and pembrolizumab have been approved by the Food and Drug Administration (FDA) to treat melanoma, lung, gastric cancer, etc. [5, 6]. Even though these drugs could prolong overall survival (OS) and progression-free survival (PFS), still only a small subset of patients benefit from these drugs [7, 8]. Therefore, it is vital to accurately identify prognostic biomarkers and the therapeutic beneficiaries in future research directions.

Ovarian cancer is the leading cause of death among patients with gynecologic malignancies [9]. About 80% of patients with serious carcinomas are diagnosed at an advanced stage [10]. Even though more than 80% of patients with advanced-stage respond to cytoreductive surgery and adjuvant chemotherapies, most of them ultimately relapse and eventually develop chemotherapy-resistant disease [11]. Recently, PARP inhibitors and Bevacizumab have been approved for ovarian cancer in addition to platinum-based therapies [12]. Immunotherapy presents a potentially novel frontier in ovarian cancer treatment, but the response rates among ovarian cancer patients is not quite high as expected. A phase II study of nivolumab (anti–PD-1 antibody) monotherapy found a 15% overall response rate (ORR) in 20 patients with platinum-resistant disease, while another combination therapy with nivolumab and bevacizumab show a 40% ORR in 38 patients with relapsed ovarian cancer in platinum-sensitive patients and 16.7% in platinum-resistant patients [7, 13]. It is evident that not all patients can benefit from immunotherapy, and patients sensitive to first-line platinum-based therapy had a higher response rate to immunotherapy than platinum-resistant patients.

This paper aims to construct a prognostic biomarker with TME and predict therapeutic effects. At present, the prognostic models consist of the commonly used prognostic indicators, such as clinicopathological characteristics and various biomarkers. However, the interaction between genes and the bias of gene expression values across platforms may lead to differences across studies. In this study, we started by systematically characterizing TME in ovarian cancer using CIBERSORT computational algorithms [14]. Then, the score with quantified TME infiltration pattern (namely TMEscore) through Principal Components Analysis (PCA) algorithm was constructed and systematically correlated with platinum-based therapy and clinical features in ovarian cancer. Finally, we explored the response to ICIs in different TMEscore groups and the association between TMEscore and mutation landscape to identify potential targets and feasible treatments.

## RESULTS

### Landscape of TME cells in ovarian cancer

**Figure 1:**
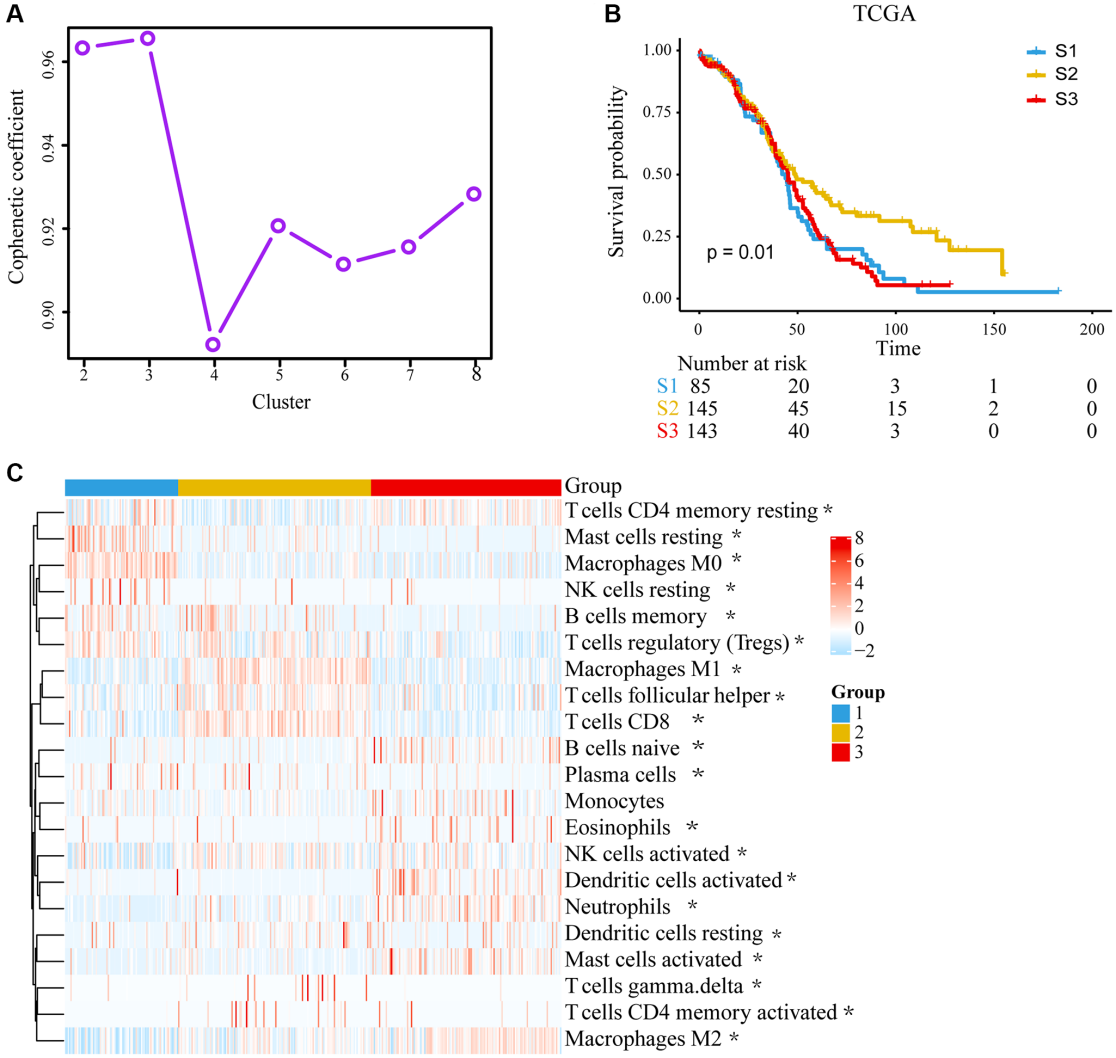
**Unsupervised clustering of tumor microenvironment (TME) cells and subtype characteristics for 373 ovarian cancer patients in the TCGA cohort.** (**A**) Cophenetic correlation coefficient of different clusters. (**B**) Kaplan–Meier (K–M) curves for overall survival (OS) of different 3 subtypes (log-rank test, *P* = 0.010). (**C**) Expression pattern of 21 TME cell types in 3 TME subtypes. The differences were confirmed by Kruskal–Wallis tests in the three TME subgroups with TME cell infiltration, and they were statistically significant except Monocytes. The asterisks represented the statistical *P* value. (^*^P < 0.05).

The workflow for this study is shown in Supplementary Figure 1. The landscape with LM22 in TCGA was analyzed by CIBERSORT computational algorithms (Supplementary Table 1). With the NMF method, cluster numbers *k* = 3 was chosen as a final cluster number because of the suitability of clustering (Figure 1A) [15]. Finally, 373 samples with TME cell expression profiles were divided into three subgroups, named TME cluster-1, TME cluster-2 and TME cluster-3. We found that TME cluster-2 had a significantly longer OS than others (*P* = 0.0102; Figure 1B). TME cluster-2 exhibited high infiltration of CD8 T cells, activated memory CD4 T cells, follicular helper T cells, M1 macrophages, gamma delta T cells and so forth. While TME cluster-1 and TME cluster-3 were characterized by increases in the infiltration of resting mast cells, resting NK cells and M0 macrophages or M2 macrophages, activated dendritic cells, neutrophils and activated mast cells, respectively (Figure 1C).

### TME signature and functional annotation

**Figure 2:**
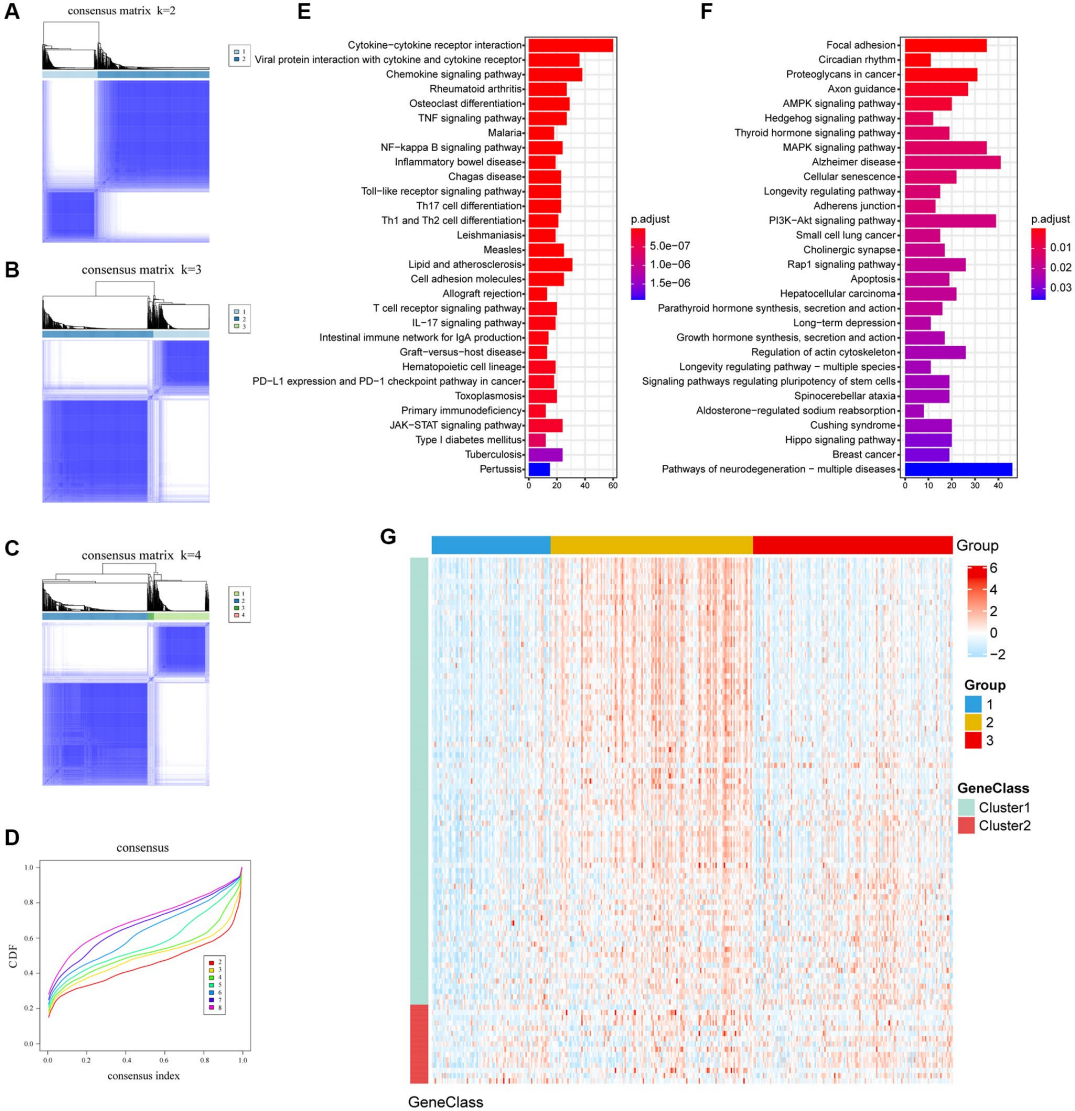
**The clusters of DEGs with consensus clustering algorithm and KEGG enrichment.** (**A**–**C**) Consensus matrixes of TCGA cohorts for each *k* (*k* = 2–4), displaying the clustering stability using 1000 iterations of hierarchical clustering. (**D**) Cumulative distribution function (CDF) curve; Different colors represent different clusters, x-axis denotes consensus index and y-axis denotes CDF values. (**E**, **F**) Enrichment pathways of the top 30 KEGG in gene cluster A and gene cluster B. (**G**) Expression profile heatmap of top 100 DEGs obtained by LIMMA and Random Forest.

We identify 1,351 TME-related DEGs, which might be associated with the biological behavior of TME. Consensus clustering was used to unsupervised learning clusters of genes (Figure 2A–2D). According to heatmap of the consensus matrices, consensus cumulative distribution function (CDF) curve, two gene clusters, termed as gene-cluster A and gene-cluster B, were identified. The functions and pathways associated with the DEGs were analyzed using KEGG and GO. The gene-cluster A enriched in Cytokine-cytokine receptor interaction pathway, Chemokine signaling pathway, Viral protein interaction with cytokine and cytokine receptor pathway and so on. In contrast, gene-cluster B enriched in Focal adhesion pathway, PI3K-Akt signaling pathway, MAPK signaling pathway, Proteoglycans in cancer pathway etc. GO enrichment (biological process) showed that gene-cluster A mainly involved T cell activation, leukocyte cell-cell adhesion and leukocyte migration; gene-cluster B were significantly enriched in axonogenesis and regulation of protein-containing complex assembly. (Figure 2E, 2F, Supplementary Tables 2, 3). And top 100 DEGs were shown in Figure 2G.

### Establishment of the TMEscore in TCGA database

**Figure 3:**
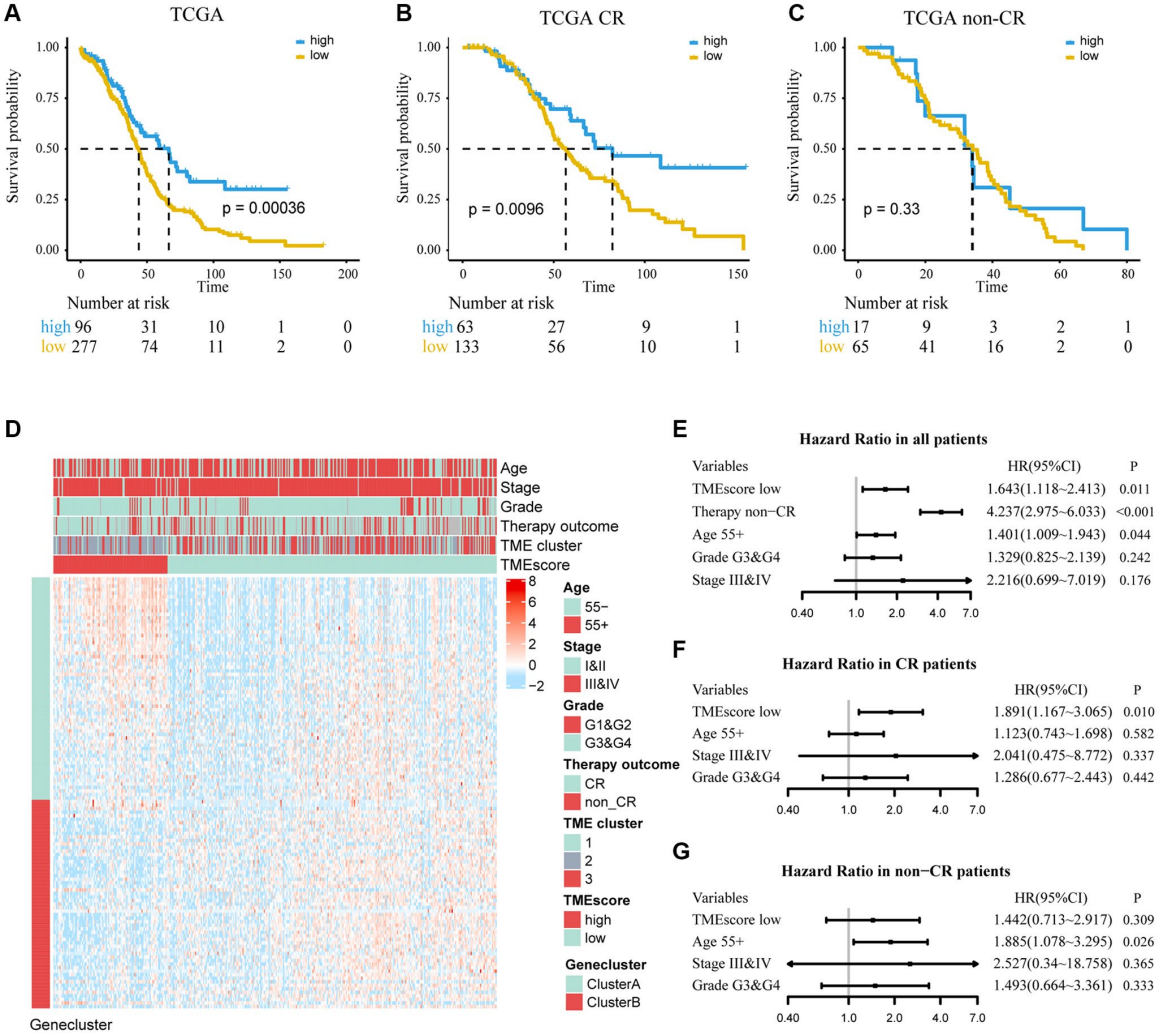
**Determine the prognostic group of 373 ovarian cancer patients based on TMEscore in TCGA and evaluate the predictive ability.** (**A**) K–M curve for OS of different TMEscore groups (log-rank test, *P* < 0.001). (**B**, **C**) According to chemotherapy outcome-stratified analysis (278 ovarian cancer patients), K–M curves in patients with complete response (CR) or non-complete response (non-CR) in different TMEscore group (log-rank test, *P* = 0.001; log-rank test, *P* = 0.33). (**D**) Expression profile of DEGs with survival significance. TMEscore, age, stage, grade, therapy outcome and TME cluster are shown as patient annotations. GeneClass is shown as gene annotations. Top legend, gray indicates missing value. (**E**–**G**) Forest plots illustrate the results of multivariate Cox proportional hazards model of clinical feature in all patients, CR patients and non-CR patients respectively.

The TMEscore was defined as $\text{TMEscore}=\sum PC1_{i}-\sum PC1_{j}.$ The 373 patients in the TCGA cohort were divided into high or low TMEscore groups based on the TMEscore cutoff value (0.83). The patients with low TMEscore had a worse prognosis in univariate analysis (HR, 1.818; 95% CI, 1.303–2.536; *P* < 0.001; Figure 3A). Adjusting for age, grade, stage and chemotherapy outcome, TMEscore (HR, 1.643; 95% CI, 1.118–2.413; *P* = 0.011) was an independent predictive factor of ovarian cancer, and its C-index was 0.688 ± 0.024 (Figure 3E). Furthermore, in subgroup analysis, we found that the TMEscore was still an independent predictive factor for patients with complete response (CR) of platinum drug chemotherapy, while TMEscore could not be identified as an independent predictive factor for non-CR (non-CR: partial response, progressive and stable disease) patients (Figure 3B, 3C and 3F, 3G). In addition, we combined CR and PR as the responders (CR and PR) and the others as the non-responders (non-CR and non-PR) and found that TMEscore could be used as an independent predictive factor in responders but not in non-responders (Supplementary Figure 2). Compared with the high TMEscore group, only a small number of DEGs in high TMEscore group were highly expressed in gene-cluster A. Meanwhile, high TMEscore group was concentrated on the CR, TME clusters-2, which were all related with better survival (Figure 3D).

### Validation of the TMEscore in GEO database

**Figure 4:**
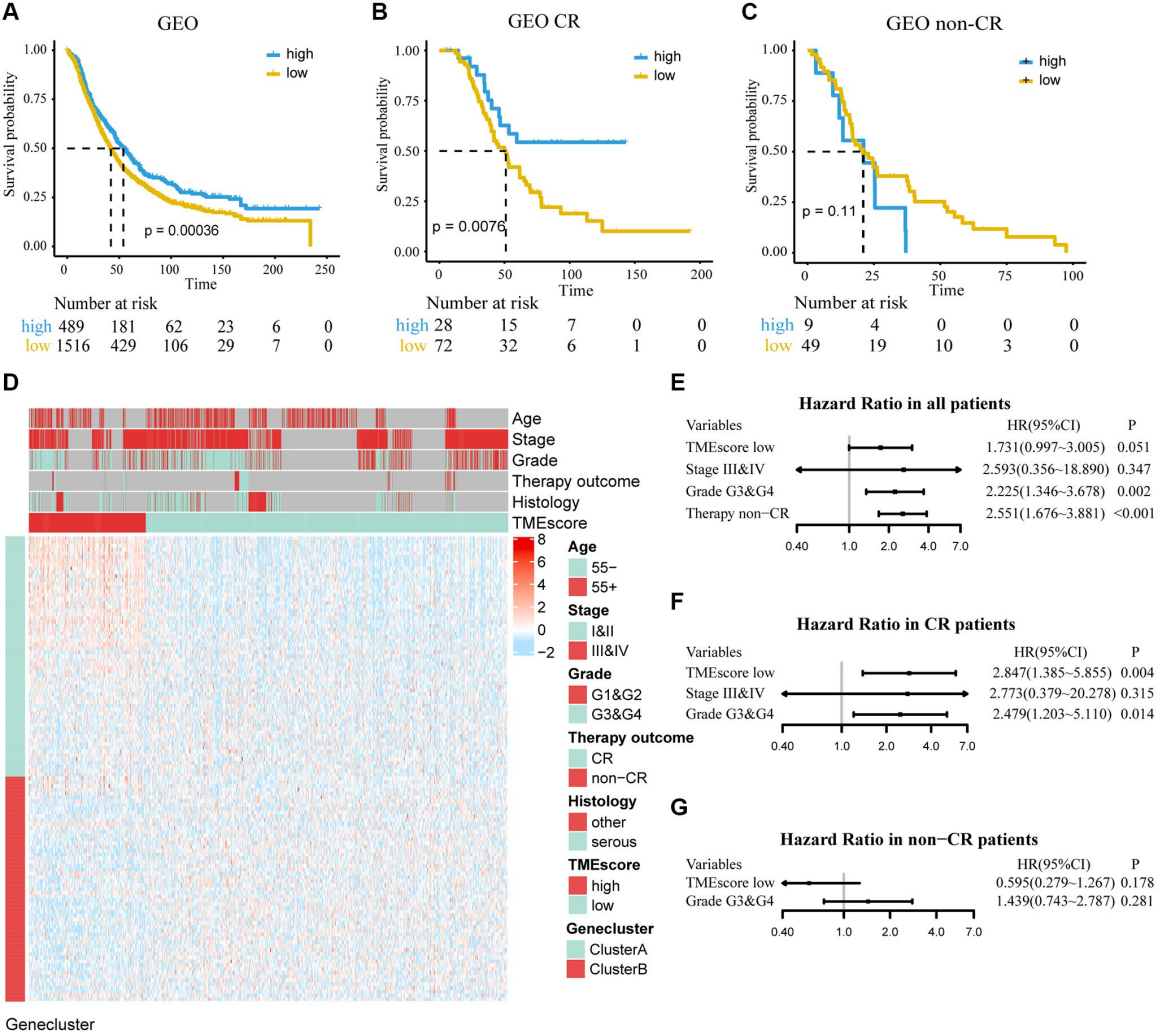
**Determine the prognostic group of 2005 ovarian cancer patients based on TMEscore in GEO and evaluate the predictive ability.** (**A**) K–M curve for OS of different TMEscore groups (log-rank test, *P* < 0.001). (**B**, **C**) According to chemotherapy outcome-stratified analysis (158 ovarian cancer patients), K–M curves in patients with complete response (CR) or non-complete response (non-CR) in different TMEscore group (log-rank test, *P* = 0.008; log-rank test, *P* = 0.11). (**D**) Expression profile of DEGs with survival significance. TMEscore, age, stage, grade, therapy outcome and histology are shown as patient annotations. Top legend, gray indicates missing value. (**E**–**G**) Forest plots illustrate the results of multivariate Cox proportional hazards model of clinical feature in all patients, CR patients and non-CR patients respectively.

We used independent datasets from the GEO database to further validate TMEscore. A total of 2,005 ovarian cancer patients were divided into high and low TMEscore groups by the same cutoff value. It was found that low TMEscore group had a worse prognosis (HR, 1.275; 95% CI, 1.116–1.457; *P* < 0.001; Figure 4A). A multivariate Cox regression model was constructed, and its C-index was 0.677 ± 0.030, in which TMEscore (HR, 1.731; 95% CI, 0.997–3.005; *P* = 0.051) was might an independent prognostic factor (Figure 4E). Expression profile of DEGs was similar to TCGA and shown in Figure 4D. Further, in subgroup analysis, we found that TMEscore had a better performance to predict the prognosis for patients who showed CR than non-CR to chemotherapy (Figure 4B, 4C and 4F, 4G). Consistent with TCGA, we redefined the chemotherapy outcome (CR and PR as responder; non-CR and non-PR as non-responder) and found TMEscore could be used as an independent predictive factor in responders (Supplementary Figure 3).

### Establish a nomogram to predict the OS of ovarian cancer

To predict mortality in ovarian cancer patients, a nomogram was drawn in the TCGA dataset to serve as a clinically relevant quantitative method and age, stage, grade and chemotherapy outcome were included respectively (Supplementary Figure 4A). Furthermore, calibration curves showed that the nomogram had similar performance to an ideal model, which could predict ovarian cancer survival at 3 and 5 years in a relatively stable manner (Supplementary Figure 4B, 4C). Due to the incomplete information on clinical features in the GEO dataset, we cannot further verify the nomogram in the GEO.

### Profile tumor somatic mutation between TMEscore groups

To comprehensively understand and explore the appropriate treatment strategy for high and low TMEscore groups, the top 30 highly mutated genes distribution were shown in Supplementary Figure 5A, 5B. The mutation rate of TP53 was the highest, reaching 87% and 92% in high and low TMEscore groups. Among a total of 13 differentially mutated genes between two groups (*P* < 0.05; Supplementary Figure 5C), eight genes such as BRCA1, OR2G6, SHROOM3 showed a higher mutation frequency in the high TMEscore group. The other five genes, such as CHD6, TECTA, had a higher mutation frequency in low TMEscore group. Subsequently, we calculated the TMB and found that TMEscore could distinguish TMB and related to patients’ OS (*P* = 0.018) (Supplementary Figure 5D).

### Expression of immune-checkpoint between TMEscore groups

**Figure 5:**
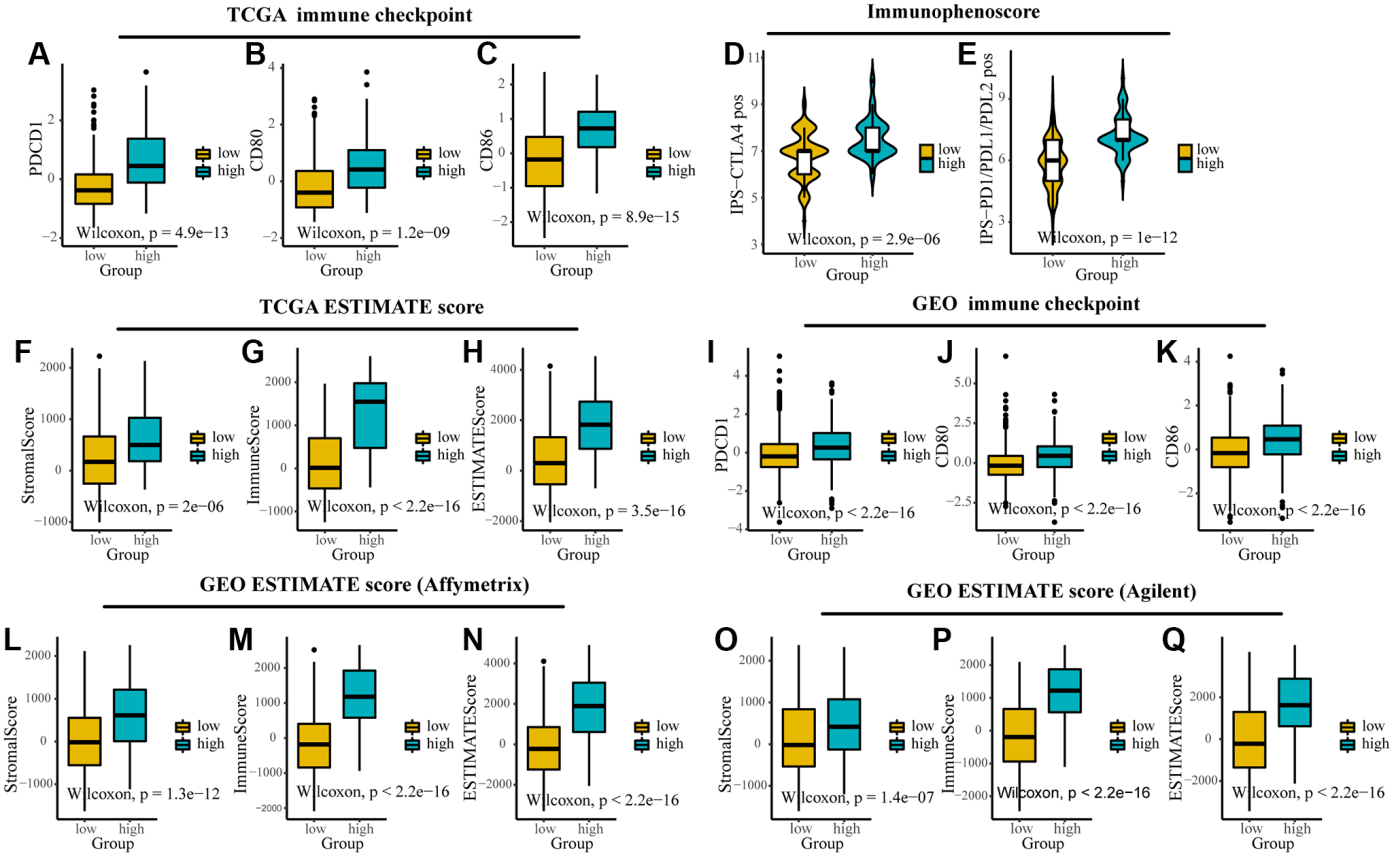
**The expression of immune checkpoints and immune-related scores between different TMEscore groups in TCGA and GEO.** (**A**–**C**) Expression of immune checkpoints (PDCD1, CD80 and CD86) between different groups in TCGA. (**D**, **E**) The relative probabilities to respond to anti-CTLA-4 and anti-PD-1/PD-L1 treatment in the low and high TMEscore group. (**F**–**I**) Expression of stromal score, immune score and ESTIMATE score between different groups in TCGA. (**J**–**L**) Expression of immune checkpoints (PDCD1, CD80 and CD86) between different groups in GEO. (**M**–**Q**) Expression of stromal score, immune score and ESTIMATE score between different groups in different platforms in GEO. The lines in the boxes represented median value.

In addition, we found that TMEscore could predict the response of ICIs and could provide a basis for subsequent immunotherapy. By comparing the expression of immune checkpoints (CD80, CD86, and PDCD1) and stromal and immune scores computed by ESTIMATE between different groups, we found that they were highly expressed in high TMEscore group in TCGA and GEO (Figure 5A–5C, 5F–5H, 5I–5K, 5L–5Q). IPS was introduced to evaluate the patients’ responses to ICI treatment because of the information on ICI treatment was not available in TCGA and GEO datasets. The IPS values (IPS-CTLA-4_pos and IPS-PD-1/PD-L1/PD-L2_pos) as the alternative of the ovarian cancer patients’ responses to anti-CTLA-4 and anti-PD-1/PD-L1 treatment were increased in the high TMEscore group (Figure 5D, 5E). It’s likely that the patients in the high TMEscore group have a better immune microenvironment and respond better to ICIs.

## DISCUSSION

The researchers have made various attempts to prolong the survival time of ovarian cancer patients by combining targeted therapy and chemotherapy [12]. Increased understanding of TME has shifted from a tumor cell centered view of cancer progression to the concept of a complex TME that supports tumor growth and metastatic dissemination. TME significantly influences therapeutic response and clinical outcomes for patients [4, 16]. In this study, TMEscore with the information of TME, developed and validated with more than 2,000 ovarian cancer patients, was found to be an independent prognostic biomarker and could improve treatment decisions in ovarian cancer.

Three distinct subtypes of ovarian cancer were found based on TME. According to previous studies, CD8 T cells were generally associated with a longer OS in tumors, while the presence of M0, M2 macrophages favoring tumor growth and spreading was associated with poor outcomes [17–19] (Figure 1). Through the enrichment of DEGs between subtypes of TME, we found that gene-cluster A was related to immune functions such as, chemokine, TNF and other cytokines act through cell surface receptors (Figure 2E). Cytokines are associated with expanding and reactivating effector NK and T lymphocytes, promoting lymphocytes tumor infiltration and persistence in TME [20]. And several biological processes correlated with immune regulation were found, including T-cell activation and leukocyte migration [21, 22]. While the pathways in gene-cluster B were usually related to cell growth, migration and proliferation (Figure 2F). Studies demonstrated that PI3K/AKT/mTOR pathway, one of the most important signaling pathways for therapeutic intervention in ovarian cancer, has been reported as the frequently altered signaling pathway in ovarian cancer [23]. And EGFR, VEGFR and BRAF targets in MAPK signaling pathway has been extensively studied for promising cancer treatment [24].

Because of characterizing and quantifying above cluster of genes, TMEscore is more biologically meaningful. At present, the predictive marker constructed by gene expression may be affected by some factors such as different platform expression and complexity of biological networks in a large number of genes. PCA is a mathematical algorithm that reduces the dimensionality of the data while retaining most of the variation in the data set. In this study, PCA was used to reduce further the dimension of the gene clusters with biological information.

Recently, numerous successful clinical trials have demonstrated that ICI treatment opens new cancer immunology avenues. However, only a small proportion of patients benefit from this therapy [25]. Therefore, we aim to find the biomarker for predicting therapeutic effect. As a significant component of the TME, immune infiltrates have been proven to mediate the tumor progression and immunotherapy responses [26]. For example, higher immune infiltration is associated with improved disease-specific survival under different treatment conditions of muscle-invasive bladder cancer. In contrast, higher stromal infiltration is associated with unfavorable disease-specific survival [27]. Meanwhile, high expression of PD-L1 and tumor-infiltrating lymphocytes are more likely to respond to ICI in advanced melanoma [28]. This study found that TMEscore had great potential in predicting ICI response. IPS values, checkpoints expression and immune score were highly expressed in high TMEscore group. Resultantly, the high TMEscore group was more likely to benefit from immunotherapy and TMEscore could be used as a biomarker to immunotherapies and individualize treatment strategies.

Furthermore, we found that BRCA1 had more mutations in the high TMEscore group and speculated that high TMEscore patients might be a beneficiary population of PARP inhibitors combined with immunotherapy. As a key gene in gynecological tumors, some studies concluded that patients with BRCA mutations have a better response to platinum therapy than those without BRCA mutations [29]. And HR (Homologous Recombination) deficient ovarian cancer may be more sensitive to PD-1/PD-L1 inhibitors, and BRCA 1 and 2 mutations may leading HR deficient [30]. Olaparib, a PARP inhibition, had been approved by EMA and FDA for ovarian cancer patients with conditions such as BRCA mutations [31]. Therefore, we believe that the PARP inhibition treatment may be feasible in the high TMEscore group. Currently, the experimental results show that the combination of Olaparib and PD-1 can further improve the clinical outcome of ovarian cancer [32]. Besides, emerging clinical data suggest additive activity between PARP inhibition and PD-1/PD-L1 blockade, regardless of BRCA1/2-mutation status and HR deficiency [33]. These results collectively illustrate that high TMEscore group could benefit from a combination of ICIs and PARP inhibition treatment, and TMEscore is a promising therapeutic predictor in ovarian cancer.

Despite the comprehensive analysis of TMEscore, there are still some limitations in this study. Firstly, the method of PCA dimension reduction of multiple genes may be inferior to the predictive factor constructed by gene expression in biological interpretation. Secondly, incomplete clinical information in GEO has decreased the statistical power in multivariable Cox regression analysis. Third, the results are based only on the public databases, so further experiments are needed to verify its biological function.

In conclusion, TMEscore was a promising prognostic marker to explore the therapeutic direction of ovarian cancer as well as possible drugs that have substantial benefit for patients. Further biological validation is needed for this exploratory study.

## MATERIALS AND METHODS

### Datasets and preprocessing

We systematically searched for ovarian cancer gene-expression datasets that were publicly available and reported with full clinical annotations in GEO (https://www.ncbi.nlm.nih.gov/geo/) [34] and TCGA (https://xenabrowser.net/datapages/) [35]. Patients without survival information were removed from further evaluation. Meanwhile, patients initially recorded as recurrent in TCGA were also removed. Finally, we gathered sixteen ovarian cancer cohorts from GEO and TCGA for this study (Table 1) and summarized the detailed medication information (Supplementary Table 4). RNA-seq and microarray gene expression data were preprocessed separately. For microarray data from Affymetrix^®^, we downloaded the raw “CEL” files and processed them using the RMA algorithm for performing background adjustment, quantile normalization etc., in the “Affy” package. The normalized matrix files were downloaded directly for microarray data from other platforms. The raw data provided by GSE13876 was processed by log2 transformation and quantile standardization. For TCGA dataset, RNA sequencing data (FPKM value) of gene expression were downloaded and transformed into transcripts per kilobase million (TPM). Probesets that mapped to more than one gene symbol were summarized by their median expression value. Genes with a missing value of more than 50% were deleted, and the remaining missing values were imputed with KNN imputation approaches. Batch effects from non-biological technical biases were corrected using the “ComBat” algorithm of “sva” package [36]. The somatic mutation data (SNPs and small INDELs) was downloaded from TCGA database (MuTect2 Variant Aggregation and Masking) (https://xenabrowser.net/datapages/) [35]. Finally, a total of 2,378 ovarian cancer patients with 6,008 genes were included in this study.

**Table 1:** Demographic and clinic features descriptions for ovarian cancer patients in TCGA and GEO.

**Characteristics**	**GSE13876**	**GSE14764**	**GSE17260**	**GSE19829**	**GSE23554**	**GSE26193**	**GSE26712**	**GSE30161**	**GSE32062**	**GSE32063**	**GSE49997**	**GSE51088**	**GSE53963**	**GSE63885**	**GSE73614**	**TCGA**
Number of samples	415	80	110	70	28	107	185	58	260	40	194	116	160	75	107	373
Median survival time (month) (95% CI)	24 (21,30)	55 (51, NA)	54 (49, NA)	46 (36,72)	53.3 (37.4, NA)	36.6 (29.9, 54.7)	46.0 (38.9, 58.0)	51.0 (32.8, 75.0)	60 (50,80)	54 (40, NA)	NA (44, NA)	42.5 (36.0, 57.0)	35.3 (26.0, 45.5)	37.5 (29.9, 43.9)	97 (73, 156)	45.2 (41.6, 49.5)
Number of Death (%)	302 (72.8)	21 (26.3)	46 (41.8)	40 (57.1)	14 (50.0)	76 (71.0)	129 (69.7)	36 (62.1)	121 (46.5)	22 (55.0)	57 (29.4)	91 (78.4)	139 (86.9)	66 (88.0)	58 (54.2)	230 (61.7)
Median PFS/DFS/RECUR time (95% CI)	–	–	19 (15, 26)	19 (15, 34)	–	21.3 (18.3, 24.5)	–	13.4 (10.9, 17.0)	19 (18, 23)	21 (15, 43)	19 (15, 21)	–	–	–	–	18.4 (16.8, 20.9)
Number of PFS/DFS/RECUR (%)															
YES	–	–	76 (69.1)	41 (58.6)	–	80 (74.8)	–	48 (82.8)	193 (74.2)	27 (67.5)	124 (63.9)	–	–	–	–	244 (65.4)
NO	–	–	34 (30.9)	14 (20.0)	–	27 (25.2)	–	6 (10.3)	67 (25.8)	13 (32.5)	70 (36.1)	–	–	–	–	129 (34.6)
unknown	–	–	–	15 (21.4)	–	–	–	4 (6.9)	–	–	–	–	–	–	–	–
Age (years)	57.95 ± 12.29	–	–	60.50 ± 11.22	–	–	–	62.57 ± 10.61	–	–	57.66 ± 11.82	60.65 ± 12.07	63.61 ± 11.41	–	61.31 ± 10.89	59.60 ± 11.37
<55	160 (38.6)	–	–	25 (35.7)	–	–	–	15 (25.9)	–	–	77 (39.7)	37 (31.9)	36 (22.5)	–	32 (29.9)	131 (35.1)
≥55	255 (61.4)	–	–	45 (64.3)	–	–	–	43 (74.1)	–	–	117 (60.3)	79 (68.1)	124 (77.5)	–	75 (70.1)	242 (64.9)
Histology type (%)	–	–	–	–	–	–	–	–	–	–	–	–	–	–	–	–
serous	–	68 (85)	–	65 (92.9)	–	79 (73.8)	–	47 (81.0)	–	–	171 (88.1)	90 (77.6)	–	70 (93.3)	4 (3.7)	373 (100.0)
others	–	12 (15)	–	5 (7.1)	–	28 (26.2)	–	9 (15.5)	–	–	23 (11.9)	26 (22.4)	–	5 (6.7)	103 (96.3)	0 (0)
unknown	–	–	–	–	–	–	–	2 (3.4)	–	–	–	–	–	–	–	–
FIGO stage (%)	–	–	–	–	–	–	–	–	–	–	–	–	–	–	–	–
I & II	–	9 (11.2)	0 (0)	3 (4.3)	–	31 (29.0)	–	0 (0)	0 (0)	0 (0)	9 (4.6)	21 (18.1)	7 (4.4)	2 (2.7)	48 (44.9)	22 (5.9)
III & IV	–	71 (88.8)	110 (100.0)	67 (95.7)	–	76 (71.0)	–	58 (100.0)	260 (100.0)	40 (100.0)	185 (95.4)	95 (81.9)	153 (95.6)	73 (97.3)	59 (55.1)	348 (93.3)
unknown	–	–	–	–	–	–	–	–	–	–	–	–	–	–	–	3 (0.8)
Grade (%)	–	–	–	–	–	–	–	–	–	–	–	–	–	–	–	–
G1 & G2 (Mod & Well)	–	26 (32.5)	67 (60.9)	10 (14.3)	10 (35.7)	40 (37.4)	–	21 (36.2)	131 (50.4)	23 (57.5)	50 (25.8)	19 (16.4)	3 (1.9)	9 (12.0)	29 (27.1)	43 (11.5)
G3 & G4 (Poor)	–	54 (67.5)	43 (39.1)	60 (85.7)	18 (64.3)	67 (62.6)	–	33 (56.9)	129 (49.6)	17 (42.5)	143 (73.7)	97 (83.6)	157 (98.1)	66 (88.0)	78 (72.9)	320 (85.8)
unknown	–	–	–	–	–	–	–	4 (6.9)	–	–	1 (0.5)	–	–	–	–	10 (2.7)
Therapy outcome	–	–	–	–	–	–	–	–	–	–	–	–	–	–	–	–
CR	–	–	–	–	18 (64.3)	–	–	32 (55.2)	–	–	–	–	–	50 (66.7)	–	196 (70.5)
non-CR	–	–	–	–	10 (35.7)	–	–	23 (39.7)	–	–	–	–	–	25 (33.3)	–	82 (29.5)
unknown	–	–	–	–	–	–	–	3 (5.2)	–	–	–	–	–	–	–	–

### Inference of infiltrating cells in TME and phenotype-associated genes

To explore TME and TME-related phenotypes in ovarian cancer, a deconvolution algorithm, CIBERSORT, was utilized to accurately quantify the proportions of immune cells in TME within a complex gene expression mixture (https://cibersort.stanford.edu/index.php). It distinguishes 22 human hematopoietic cell phenotypes (LM22), including seven T-cell types, naïve and memory B cells, plasma cells, natural killer (NK) cells and myeloid subsets. Next, nonnegative matrix factorization (NMF), an unsupervised algorithm, was used to find the cluster of TME-infiltrating cells. The differentially expressed genes (DEGs) associated with the TME phenotype were determined by both linear models and nonlinear models for RNA-seq data. The linear model was performed using the “limma” package with adjusted *P* < 0.05 [37]. Random forest classification algorithm was used as the non-linear model for DEGs identification.

### TME gene signature

To predict the prognosis of ovarian cancer, TMEscore was constructed by quantifying the TME characterization of individual tumor. A three-step method was used to establish TMEscore. First, consensus clustering algorithm was applied to define the cluster of DEGs, as DEGs may be related to different biological functions in TME. Then, the “clusterProfiler” package [38] was adopted to annotate gene patterns of Kyoto Encyclopedia of Genes and Genomes (KEGG) pathway and Gene Ontology (GO) enrichment [39, 40]. Furthermore, univariate Cox regression was performed to identify prognostic genes for each gene (transformed into a z-score) in the cluster. Moreover, PCA was used to construct TME relevant gene signature. Principal component 1 was selected to act as signature scores. Finally, TMEscore was calculated similar to GGI method [41]:


$$\text{TMEscore}=\sum PC1_{i}-\sum PC1_{j}$$

where *i* is the signature score of clusters whose Cox coefficient is negative, and *j* is the expression of genes whose Cox coefficient is positive. This method has the advantage of focusing the score on the set with the largest block of well correlated (or anticorrelated) genes in the set, while down-weighting contributions from genes that do not track with other set members.

### A prognostic nomogram with TMEscore

To determine the prognostic significance of TMEscore in ovarian cancer, log-rank test and multivariate Cox regression model were fitted in this analysis. Meanwhile, to eliminate the influence of chemotherapy outcome on prognosis, we performed a chemotherapy outcome-stratified analysis of all patients with therapeutic information. A nomogram is considered an important component of decision-making in modern medicine. It could generate an individual possibility of a clinical event by integrating various prognostic and determinant variables, providing assistance for personalized medicine [42]. Therefore, we constructed a nomogram with age, stage, grade, chemotherapy outcome and TMEscore to predict the probability of 3- and 5-year OS. The calibration curve was drawn to evaluate the nomogram prediction possibilities against the observed rates.

### Identification of gene mutations

Some mutant-specific drugs are emerging as the preferred first-line therapy for cancers [43]. We combined the mutation information with TMEscore to explore appropriate treatment of different TMEscore groups and improve the treatment strategy. The “Maftools” package was used to present the mutation landscape, identify the differentially genes mutations between groups and calculate tumor mutational burden (TMB).

### Predicting the patients’ response to ICI

The Cancer Immunome Atlas (https://tcia.at/) analyzed the immune landscapes and antigenomes of 20 solid tumors and were quantified by Immunophenoscore [44] (IPS, a superior immune response molecular marker). The IPS value, ranged from 0 to 10, was positively correlated to tumor immunogenicity and has been verified that IPS could predict the patients’ response to immune checkpoints inhibitors (ICIs treatment). The immune checkpoint expressions between different TMEscore groups were observed intuitively by drawing box and violin diagrams. The fraction of stromal and immune cells was inferred using the ESTIMATE method [45].

### Statistical analysis

Continuous variables were summarized as mean ± SD and categorized variables were described by frequency (n) and proportion (%). Wilcoxon rank-sum test or Kruskal-Wallis test were used to compare two or three groups of quantitative variables. For comparisons of qualitative variables, statistical significance was estimated by Fisher’s exact tests. The cutoff value was calculated based on the correlation between the patients’ survival and the TMEscore in TCGA using “surv_cutpoint” function with the “survminer” package. The “surv_cutpoint” function, which repeatedly tested all potential thresholds in order for the maximum rank statistic, was applied to dichotomize TMEscore. Kaplan–Meier (K–M) curves were generated to illustrate the prognostic analysis and log-rank tests were utilized to identify significance of differences. The concordance index (C-index) was calculated to investigate performance of the TMEscore prognostic model. The nomogram and calibration curve were generated with “rms” package. The heatmap was produced by R package “ComplexHeatmap”. We list all R packages used in this paper in Supplementary Table 5. All statistical *P* values were two sides, with *P* < 0.05 as statistically significance. All data processing was done in R 4.0.2 software.

## Supplementary Materials

Supplementary Figures

Supplementary Tables 1-3

Supplementary Tables 4 and 5
