# Supplementary material for: Improving ovarian cancer treatment decision using a novel risk predictive tool
Source: Aging (Albany NY). 2022 Apr 19;14(8):3464–83. doi: 10.18632/aging.204023 (PMC9085236; doi:10.18632/aging.204023)
Supplement: Supplementary Figures [file aging-14-204023-s001.pdf]

SUPPLEMENTARY FIGURES

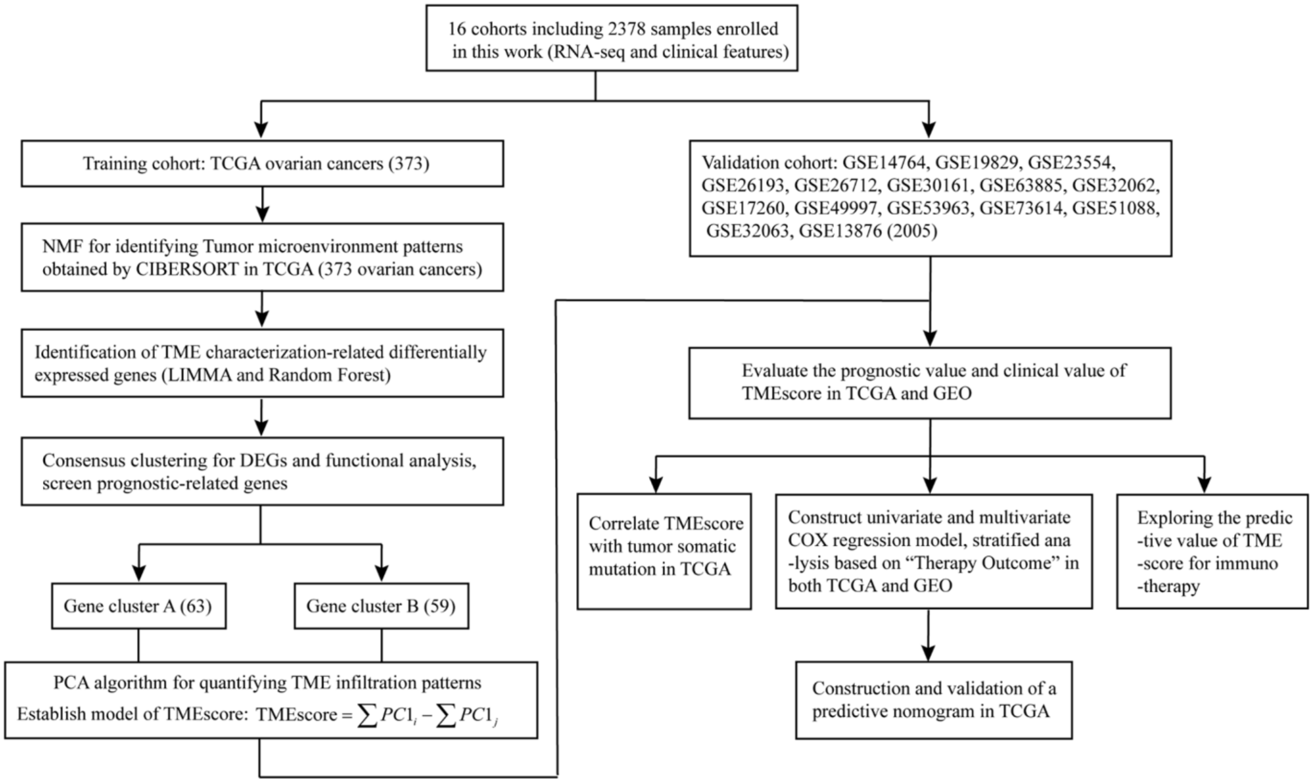

Supplementary Figure 1. Overview of study design.

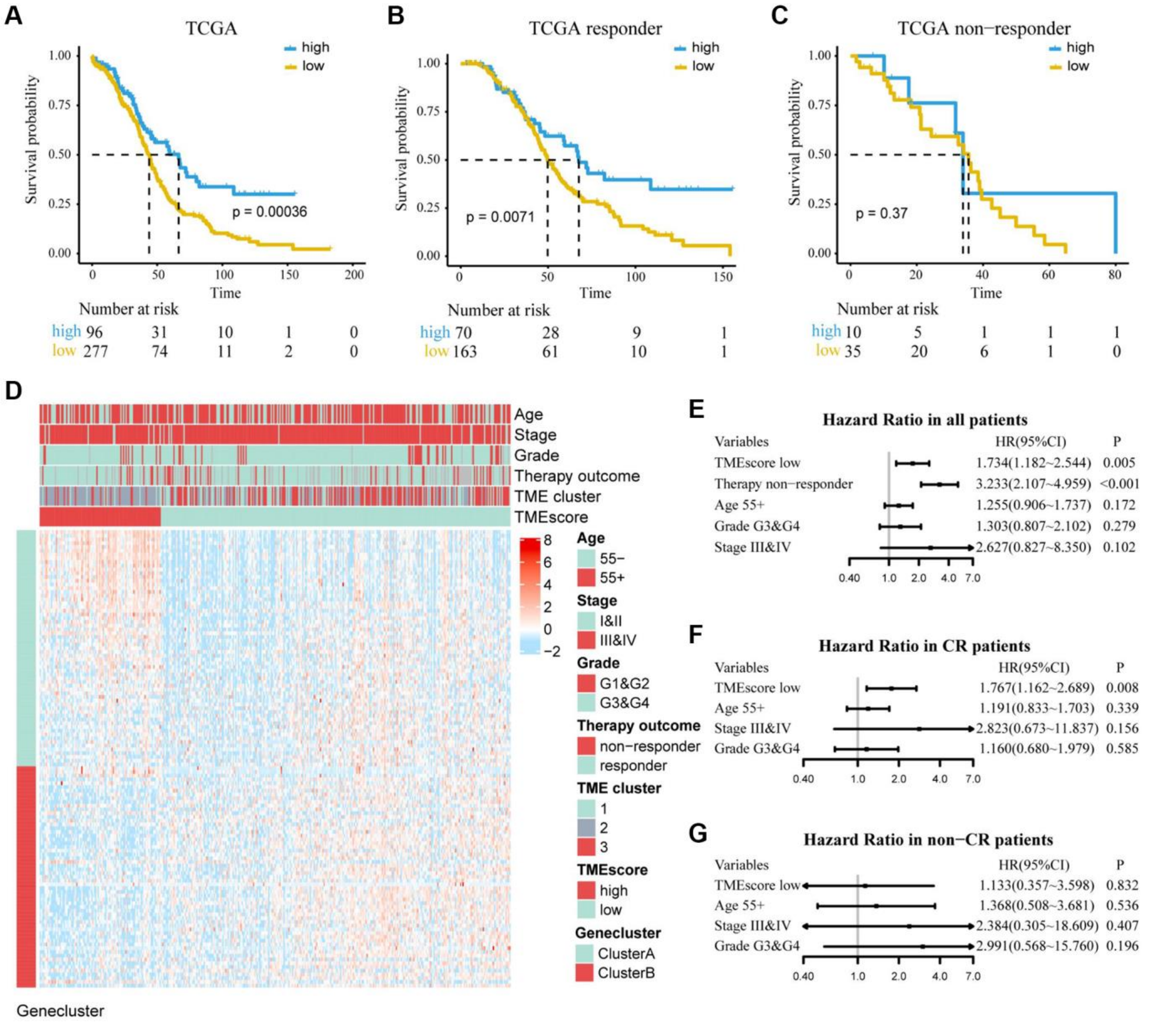

Supplementary Figure 2. Sensitivity analysis determine the prognostic group of 373 ovarian cancer patients based on TMEscore in TCGA and evaluate the predictive ability. (**A**) K–M curve for OS of different TMEscore groups (log-rank test, $P < 0.001$). (**B**, **C**) According to chemotherapy outcome-stratified analysis (278 ovarian cancer patients), K–M curves in patients with responders (CR and PR) or non-responders (non-CR and non-PR) in different TMEscore group (log-rank test, $P = 0.007$; log-rank test, $P = 0.37$). (**D**) Expression profile of DEGs with survival significance. TMEscore, age, stage, grade, therapy outcome and TME cluster are shown as patient annotations. GeneClass is shown as gene annotations. Top legend, gray indicates missing value. (**E**–**G**) Forest plots illustrate the results of multivariate Cox proportional hazards model of clinical feature in all patients, responders and non-responders respectively.

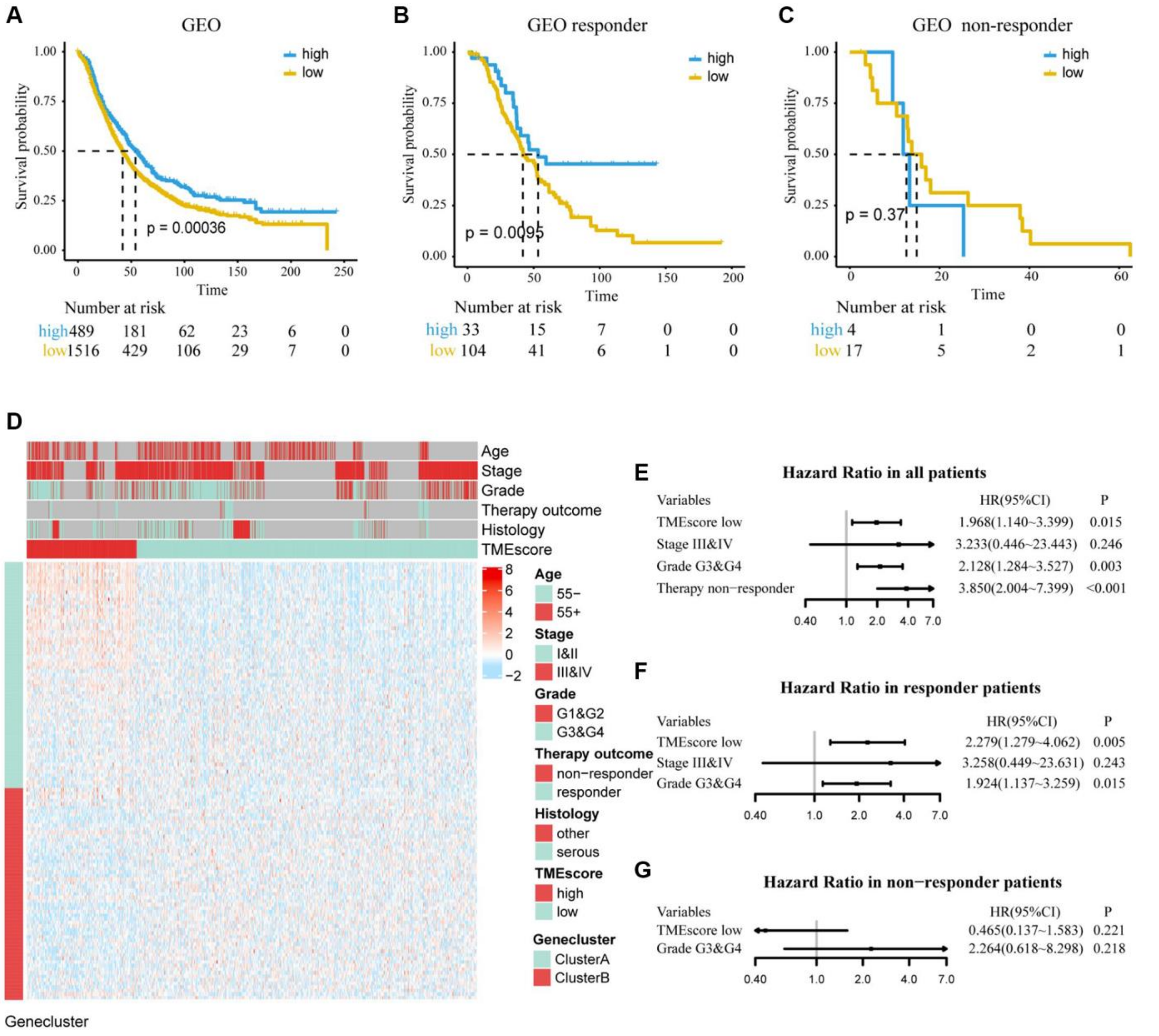

Supplementary Figure 3. Sensitivity analysis determine the prognostic group of 2005 ovarian cancer patients based on TMEscore in GEO and evaluate the predictive ability. (**A**) K–M curve for OS of different TMEscore groups (log-rank test, $P < 0.001$). (**B**, **C**) According to chemotherapy outcome-stratified analysis (158 ovarian cancer patients), K–M curves in patients with responders (CR and PR) or non-responders (non-CR and non-PR) in different TMEscore group (log-rank test, $P = 0.0095$; log-rank test, $P = 0.37$). (**D**) Expression profile of DEGs with survival significance. TMEscore, age, stage, grade, therapy outcome and histology are shown as patient annotations. Top legend, gray indicates missing value. (**E**–**G**) Forest plots illustrate the results of multivariate Cox proportional hazards model of clinical feature in all patients, responders and non-responders respectively.

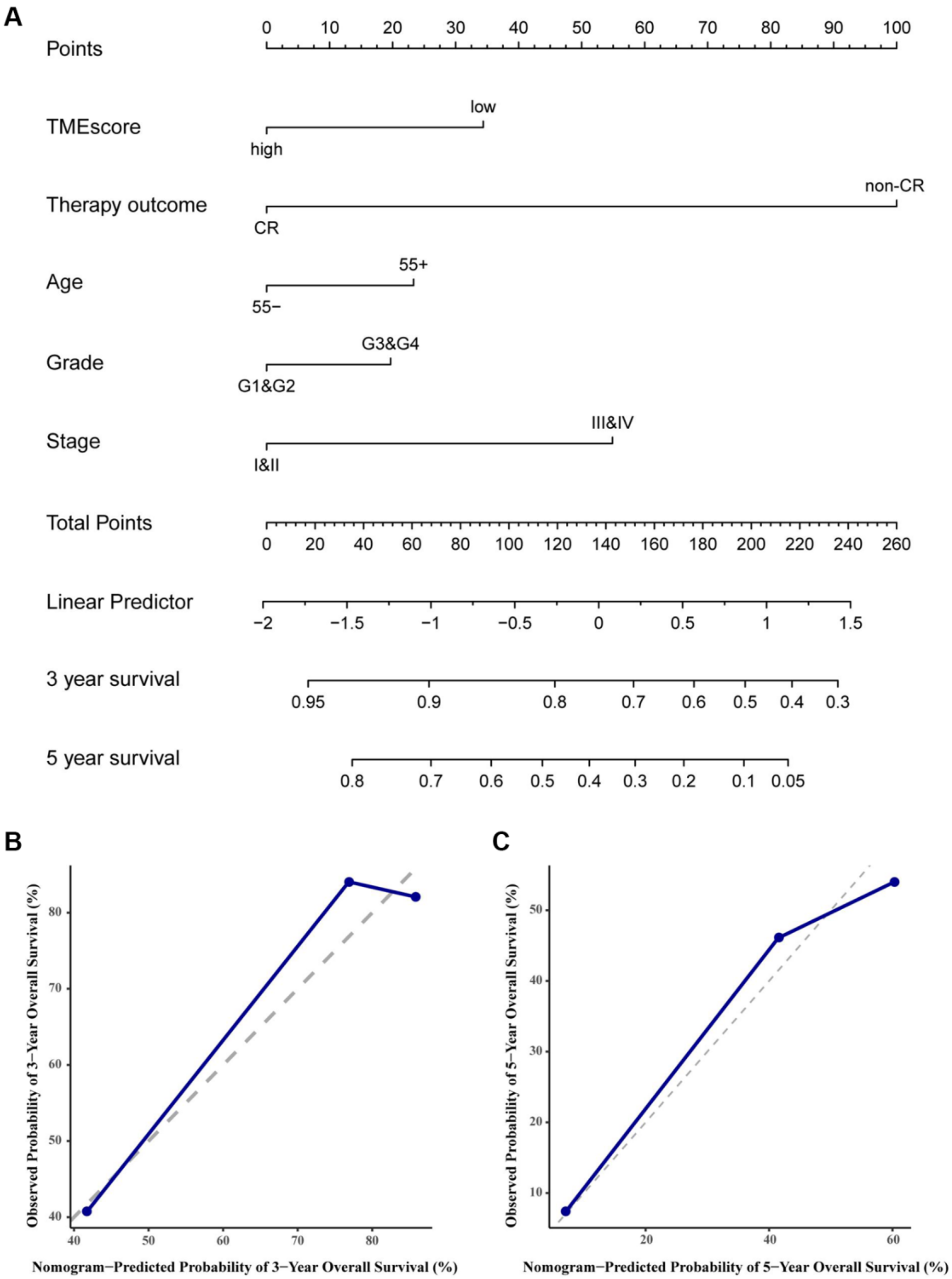

Supplementary Figure 4. Nomogram of clinical features in TCGA. (**A**) Nomogram for predicting the probability at 3- or 5-year OS based on clinical features. (**B**, **C**) Calibration curves of the nomogram for predicting the probability of OS at 3-, and 5-years.

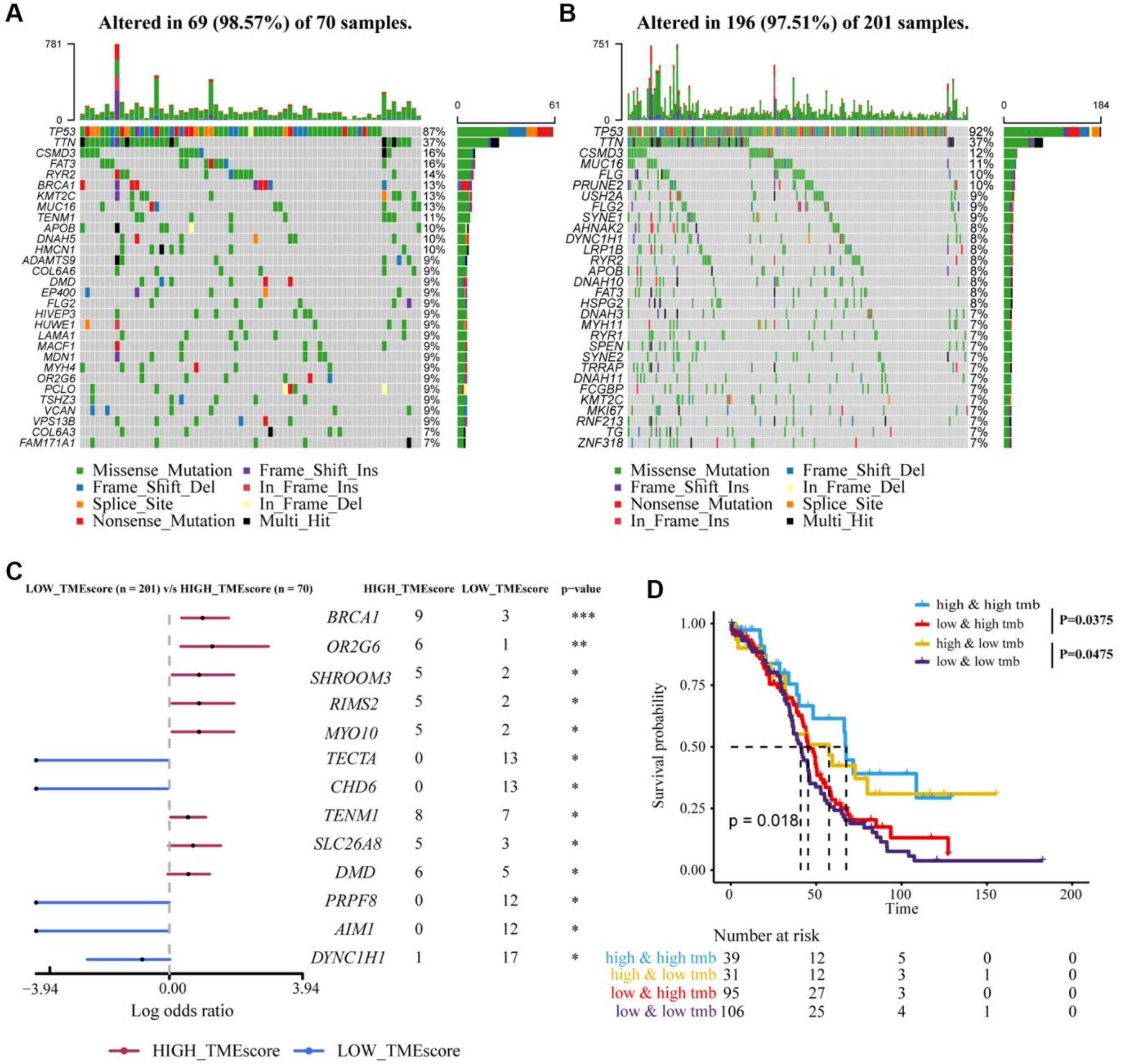

Supplementary Figure 5. The mutation landscape between high and low TMEscore groups. (**A**, **B**) Mutation profile of high and low TMEscore groups. (**C**) Comparing differential mutated genes between TMEscore groups by Fisher's exact tests. The asterisks represented the statistical *P* value. ($^{*}P < 0.05$; $^{**}P < 0.01$; $^{***}P < 0.001$). (**D**) K–M curves for patients in TCGA cohort stratified by both receipt of tumor mutational burden (TMB, cutoff value was calculated by median) and TMEscore (log-rank test, $P = 0.018$).
