# Supplementary material for: Improving ovarian cancer treatment decision using a novel risk predictive tool
Source: Aging (Albany NY). 2022 Apr 19;14(8):3464–83. doi: 10.18632/aging.204023 (PMC9085236; doi:10.18632/aging.204023)
Supplement: Supplementary Tables 1-3 [file aging-14-204023-s002.docx]

**Supplementary Table 1. The proportions of tumor microenvironment cells of 373 ovarian cancer patients.**

| Input Sample | B cells naive | B cells memory | Plasma cells | T cells CD8 | T cells CD4 naive | T cells CD4 memory resting | T cells CD4 memory activated | T cells follicular helper | T cells regulatory (Tregs) | T cells gamma delta | NK cells resting | NK cells activated | Monocytes | Macrophages M0 | Macrophages M1 | Macrophages M2 | Dendritic cells resting | Dendritic cells activated | Mast cells resting | Mast cells activated | Eosinophils | Neutrophils | P-value | Pearson Correlation | RMSE |
| --- | --- | --- | --- | --- | --- | --- | --- | --- | --- | --- | --- | --- | --- | --- | --- | --- | --- | --- | --- | --- | --- | --- | --- | --- | --- |
| TCGA-04-1331-01A | 0 | 0.082013 | 0 | 0.057985 | 0 | 0.115685 | 0 | 0.054142 | 0.105353 | 0 | 0 | 0.088273 | 0.021529 | 0.211569 | 0.122827 | 0.045595 | 0 | 0 | 0.078943 | 0 | 0.016086 | 0 | 0.2 | 0.108377 | 1.055784 |
| TCGA-04-1332-01A | 0 | 0.0003 | 0 | 0.079907 | 0 | 0.166873 | 0 | 0.026285 | 0.079668 | 0 | 0 | 0.100286 | 0.015768 | 0.021229 | 0.053016 | 0.350116 | 0.021841 | 0 | 0.058087 | 0 | 0 | 0.026641 | 0.4 | 0.033514 | 1.081302 |
| TCGA-04-1338-01A | 0.0001 | 0 | 0 | 0.168507 | 0 | 0.110944 | 0.037491 | 0.057661 | 0.001758 | 0 | 0 | 0.087847 | 0.004531 | 0 | 0.081838 | 0.38959 | 0 | 0 | 0 | 0.047036 | 0.01039 | 0.002354 | 0 | 0.291576 | 0.973102 |
| TCGA-04-1341-01A | 0 | 0.079163 | 0 | 0.139495 | 0 | 0.229343 | 0 | 0.06342 | 0.117701 | 0 | 0 | 0.063272 | 0.00437 | 0.139089 | 0.083223 | 0.027711 | 0 | 0.032872 | 0.020342 | 0 | 0 | 0 | 0.3 | 0.086171 | 1.074247 |
| TCGA-04-1343-01A | 0 | 0.083322 | 0 | 0.145513 | 0 | 0.147752 | 0 | 0.078147 | 0.074263 | 0.003121 | 0 | 0.070284 | 0.006117 | 0.026468 | 0.05664 | 0.208298 | 0 | 0 | 0 | 0.100075 | 0 | 0 | 0.15 | 0.125392 | 1.038693 |
| TCGA-04-1347-01A | 0 | 0 | 0 | 0.072959 | 0 | 0.054188 | 0 | 0.047673 | 0.111858 | 0 | 0 | 0 | 0 | 0.356284 | 0.023181 | 0.172919 | 0 | 0.025415 | 0.121244 | 0 | 0 | 0.014278 | 0.42 | 0.028613 | 1.135631 |
| TCGA-04-1350-01A | 0 | 0 | 0 | 0.407502 | 0 | 0 | 0 | 0.07127 | 0.102787 | 0 | 0 | 0 | 0.012206 | 0.207126 | 0.013641 | 0.005674 | 0 | 0 | 0.179792 | 0 | 0 | 0 | 0.54 | 0.015521 | 1.119314 |
| TCGA-04-1356-01A | 0.003032 | 0.004945 | 0.007984 | 0.099481 | 0 | 0 | 0 | 0.049389 | 0.089464 | 0.009571 | 0.024065 | 0 | 0 | 0.361715 | 0.032324 | 0.121718 | 0 | 0 | 0 | 0.176326 | 0 | 0.019985 | 0.04 | 0.221305 | 1.043154 |
| TCGA-04-1361-01A | 0 | 0.007939 | 0 | 0.148298 | 0 | 0.142391 | 0 | 0.004013 | 0.123026 | 0 | 0 | 0.063705 | 0.011087 | 0 | 0.025188 | 0.320772 | 0.0007 | 0.010969 | 0.126894 | 0 | 0 | 0.01499 | 0.16 | 0.12214 | 1.038287 |
| TCGA-04-1362-01A | 0.024012 | 0 | 0 | 0.043271 | 0 | 0.156593 | 0 | 0.025179 | 0.062463 | 0 | 0 | 0.088864 | 0.090702 | 0 | 0.007101 | 0.301108 | 0 | 0.040604 | 0 | 0.157188 | 0 | 0.002915 | 0.33 | 0.062135 | 1.060723 |
| TCGA-04-1364-01A | 0.383284 | 0 | 0.011866 | 0.036028 | 0 | 0.240629 | 0 | 0.012787 | 0.052414 | 0 | 0 | 0 | 0 | 0.260749 | 0 | 0 | 0 | 0 | 0 | 0 | 0.002242 | 0 | 0.31 | 0.066521 | 1.100755 |
| TCGA-04-1365-01A | 0 | 0.04723 | 0.010727 | 0.193369 | 0 | 0 | 0 | 0.07453 | 0.069794 | 0 | 0 | 0.038245 | 0.021757 | 0.11251 | 0.119131 | 0.208222 | 0.022974 | 0 | 0 | 0.051992 | 0 | 0.029519 | 0 | 0.341434 | 0.947154 |
| TCGA-04-1514-01A | 0.125529 | 0 | 0 | 0.233424 | 0 | 0.08264 | 0 | 0 | 0.106113 | 0 | 0 | 0.108296 | 0.031723 | 0.0001 | 0.112944 | 0.191468 | 0 | 0 | 0 | 0 | 0 | 0.007767 | 0.63 | 0.002183 | 1.114635 |
| TCGA-04-1519-01A | 0 | 0.029012 | 0 | 0 | 0 | 0.274484 | 0 | 0.020532 | 0.126063 | 0 | 0 | 0.037773 | 0 | 0.141383 | 0.001832 | 0.075577 | 0 | 0 | 0.274866 | 0 | 0 | 0.018477 | 0.33 | 0.055135 | 1.069933 |
| TCGA-04-1530-01A | 0.021089 | 0 | 0 | 0.060693 | 0 | 0.144233 | 0 | 0.086558 | 0.012096 | 0 | 0 | 0.029993 | 0.002772 | 0.061387 | 0.166034 | 0.296129 | 0.039754 | 0 | 0 | 0.079262 | 0 | 0 | 0 | 0.376524 | 0.933605 |
| TCGA-04-1536-01A | 0 | 0.002075 | 0 | 0.079863 | 0 | 0.125727 | 0 | 0.028G07 | 0.062314 | 0 | 0 | 0.086887 | 0.100653 | 0 | 0.040686 | 0.291784 | 0 | 0.008784 | 0 | 0.109988 | 0.026139 | 0.037031 | 0.33 | 0.056455 | 1.064309 |
| TCGA-04-1542-01A | 0.007386 | 0 | 0 | 0.071969 | 0 | 0.243871 | 0 | 0.019124 | 0.111248 | 0 | 0 | 0.037685 | 0.020812 | 0.002128 | 0.013197 | 0.301401 | 0 | 0.148601 | 0 | 0 | 0 | 0.022577 | 0.72 | -0.0105 | 1.10537 |
| TCGA-04-1648-01A | 0.0007 | 0.001316 | 0.00884 | 0.073747 | 0 | 0.160497 | 0 | 0.014909 | 0.078466 | 0 | 0 | 0.042342 | 0 | 0 | 0.037629 | 0.226225 | 0.038606 | 0 | 0.158906 | 0 | 0 | 0.157838 | 0.36 | 0.050218 | 1.06137 |
| TCGA-04-1651-01A | 0.048556 | 0 | 0 | 0.020371 | 0 | 0.161759 | 0.049935 | 0.094046 | 0.049923 | 0 | 0 | 0.181824 | 0.032003 | 0 | 0.050112 | 0.127204 | 0 | 0.148332 | 0 | 0 | 0.014356 | 0.02158 | 0.33 | 0.055175 | 1.085062 |
| TCGA-04-1655-01A | 0 | 0 | 0.050002 | 0.104665 | 0 | 0.044803 | 0 | 0.055004 | 0.033046 | 0 | 0 | 0.100648 | 0 | 0.237637 | 0 | 0 | 0.001738 | 0 | 0.372458 | 0 | 0 | 0 | 0.9 | -0.0287 | 1.129035 |
| TCGA-09-0364-01A | 0.266414 | 0 | 0 | 0.033698 | 0 | 0.211423 | 0 | 0.056884 | 0 | 0 | 0 | 0.132544 | 0.017804 | 0 | 0 | 0.204674 | 0 | 0.019706 | 0 | 0 | 0.047565 | 0.009289 | 0.42 | 0.03111 | 1.084278 |
| TCGA-09-0366-01A | 0.019893 | 0 | 0 | 0.104804 | 0 | 0.023374 | 0 | 0.012721 | 0.046621 | 0 | 0 | 0.056307 | 0.202792 | 0 | 0.010463 | 0.363915 | 0 | 0.019613 | 0 | 0.102638 | 0.028335 | 0.008524 | 0.05 | 0.215709 | 1.005384 |
| TCGA-09-0367-01A | 0 | 0.085481 | 0 | 0.055041 | 0 | 0.188003 | 0 | 0.040521 | 0.078853 | 0 | 0 | 0.094506 | 0.003177 | 0.093787 | 0.025885 | 0.190433 | 0.053107 | 0 | 0 | 0.064975 | 0 | 0.026231 | 0.18 | 0.116423 | 1.039864 |
| TCGA-09-0369-01A | 0.007711 | 0 | 0.007149 | 0 | 0 | 0.167918 | 0 | 0.00185 | 0.066481 | 0 | 0.039035 | 0 | 0.02308 | 0.32699 | 0.024002 | 0.290284 | 0 | 0 | 0 | 0.045499 | 0 | 0 | 0.09 | 0.176107 | 1.072264 |
| TCGA-09-1659-01B | 0 | 0 | 0 | 0.103864 | 0 | 0 | 0 | 0.015687 | 0.092162 | 0 | 0 | 0.140223 | 0.023739 | 0.264091 | 0.008309 | 0.318176 | 0 | 0 | 0 | 0.033555 | 0 | 0.0002 | 0.15 | 0.128469 | 1.089163 |
| TCGA-09-1661-01B | 0 | 0.008572 | 0 | 0.138668 | 0 | 0.217768 | 0 | 0.037877 | 0.066499 | 0 | 0 | 0.018738 | 0.037812 | 0.060367 | 0.018853 | 0.216386 | 0 | 0.036716 | 0.135824 | 0 | 0 | 0.005921 | 0.3 | 0.074882 | 1.057608 |
| TCGA-09-1662-01A | 0.00968 | 0 | 0 | 0.051331 | 0 | 0.203413 | 0 | 0.01368 | 0.066395 | 0 | 0 | 0.092801 | 0.013841 | 0.178282 | 0.013873 | 0.259426 | 0 | 0 | 0 | 0.078507 | 0 | 0.018772 | 0.03 | 0.234723 | 1.005571 |
| TCGA-09-1665-01B | 0 | 0.164211 | 0 | 0.133101 | 0 | 0 | 0 | 0.085075 | 0.078509 | 0.010732 | 0 | 0.085936 | 0.007823 | 0 | 0.163486 | 0.220028 | 0.002941 | 0.001424 | 0 | 0.03752 | 0 | 0.009214 | 0 | 0.262511 | 0.987485 |
| TCGA-09-1666-01A | 0.018112 | 0 | 0 | 0.120142 | 0 | 0.146468 | 0 | 0.078192 | 0.051721 | 0 | 0 | 0.076108 | 0 | 0.07045 | 0.139241 | 0.225098 | 0.001428 | 0 | 0 | 0.046823 | 0 | 0.026219 | 0 | 0.281346 | 0.980727 |
| TCGA-09-1667-01C | 0 | 0.018219 | 0 | 0.096003 | 0 | 0.078108 | 0 | 0.097388 | 0.074016 | 0 | 0 | 0.082557 | 0 | 0.155924 | 0.151042 | 0.11464 | 0.025691 | 0 | 0 | 0.106412 | 0 | 0 | 0 | 0.338432 | 0.953757 |
| TCGA-09-1668-01B | 0.09326 | 0 | 0 | 0.06793 | 0 | 0.166201 | 0.016528 | 0.053843 | 0.024385 | 0 | 0 | 0.062024 | 0 | 0.115301 | 0.158178 | 0.134189 | 0.002249 | 0 | 0 | 0.095858 | 0 | 0.010053 | 0.12 | 0.142757 | 1.046107 |
| TCGA-09-1669-01A | 0.007124 | 0 | 0 | 0.160877 | 0 | 0.15079 | 0 | 0.092622 | 0.093596 | 0 | 0 | 0.067793 | 0.020203 | 0.084586 | 0.097489 | 0.182511 | 0.0009 | 0 | 0.041542 | 0 | 0 | 0 | 0.01 | 0.247982 | 0.994092 |
| TCGA-09-1670-01A | 0.004361 | 0.015123 | 0 | 0.072541 | 0 | 0.202892 | 0 | 0.025723 | 0.091085 | 0 | 0 | 0.11441 | 0.009103 | 0 | 0.048383 | 0.279458 | 0 | 0.009364 | 0 | 0.040286 | 0 | 0.087271 | 0.17 | 0.120931 | 1.044567 |
| TCGA-09-1673-01A | 0 | 0 | 0.006451 | 0.098072 | 0 | 0.0633 | 0 | 0.051512 | 0.120589 | 0 | 0 | 0.107724 | 0.023967 | 0.169316 | 0.032889 | 0.059456 | 0 | 0 | 0.259867 | 0 | 0 | 0.006856 | 0.3 | 0.075942 | 1.063337 |
| TCGA-09-2044-01B | 0 | 0.123062 | 0 | 0.116104 | 0 | 0.015951 | 0 | 0.042212 | 0.08546 | 0 | 0 | 0.005467 | 0.009583 | 0 | 0.05147 | 0.40034 | 0 | 0 | 0 | 0.126307 | 0 | 0.024043 | 0.13 | 0.142167 | 1.030975 |
| TCGA-09-2045-01A | 0.019132 | 0 | 0 | 0.083572 | 0 | 0.184746 | 0 | 0.0242 | 0.090863 | 0 | 0 | 0.086844 | 0.009365 | 0 | 0.012482 | 0.415702 | 0 | 0.051222 | 0 | 0.001342 | 0 | 0.020531 | 0.3 | 0.076283 | 1.069947 |
| TCGA-09-2048-01A | 0 | 0.02693 | 0.063164 | 0.121792 | 0 | 0.056001 | 0 | 0.050684 | 0.06598 | 0 | 0 | 0.043954 | 0.021332 | 0 | 0.041951 | 0.15275 | 0.007139 | 0 | 0 | 0.299471 | 0.027586 | 0.021267 | 0.98 | -0.0533 | 1.106647 |
| TCGA-09-2051-01A | 0.014267 | 0 | 0.078779 | 0.137258 | 0 | 0.005118 | 0 | 0.089673 | 0.112101 | 0 | 0 | 0.144208 | 0.030664 | 0.073762 | 0.050755 | 0.247768 | 0 | 0.009275 | 0 | 0.00637 | 0 | 0 | 0.11 | 0.166391 | 1.025552 |
| TCGA-09-2053-01C | 0.075226 | 0 | 0.047025 | 0.062619 | 0 | 0.12312 | 0 | 0.068845 | 0.038143 | 0 | 0 | 0.09227 | 0 | 0.047265 | 0.135332 | 0.287851 | 0 | 0 | 0 | 0.022304 | 0 | 0 | 0 | 0.33093 | 0.951739 |
| TCGA-09-2054-01A | 0.003616 | 0.015216 | 0.023838 | 0.098265 | 0 | 0.206675 | 0 | 0.03934 | 0.064664 | 0 | 0 | 0.087873 | 0.033585 | 0.109549 | 0.064602 | 0.174218 | 0 | 0 | 0.078559 | 0 | 0 | 0 | 0.3 | 0.074674 | 1.061483 |
| TCGA-09-2056-01B | 0 | 0.046233 | 0 | 0.19299 | 0 | 0.096144 | 0 | 0 | 0.058448 | 0 | 0 | 0.107927 | 0.004602 | 0.067019 | 0.066089 | 0.147473 | 0.067798 | 0 | 0 | 0.064962 | 0 | 0.080314 | 0.14 | 0.139863 | 1.038332 |
| TCGA-10-0927-01A | 0 | 0.005027 | 0.074272 | 0.226608 | 0 | 0.061508 | 0 | 0.034919 | 0.07219 | 0 | 0 | 0.16825 | 0.009219 | 0.139679 | 0 | 0.0011 | 0.016929 | 0 | 0.190301 | 0 | 0 | 0 | 0.61 | 0.004745 | 1.112301 |
| TCGA-10-0928-01A | 0 | 0.029323 | 0.001257 | 0 | 0 | 0.240823 | 0 | 0.020552 | 0.112809 | 0 | 0.035158 | 0 | 0.01499 | 0 | 0 | 0.0007 | 0.025407 | 0.014355 | 0 | 0.500424 | 0 | 0.004206 | 0.41 | 0.032288 | 1.129161 |
| TCGA-10-0931-01A | 0 | 0.057882 | 0 | 0.213233 | 0 | 0.029611 | 0 | 0.05082 | 0.10512 | 0 | 0 | 0.145786 | 0.02107 | 0.013264 | 0.020893 | 0.119198 | 0 | 0.018665 | 0 | 0.10095 | 0.086682 | 0.016826 | 0.33 | 0.062706 | 1.072523 |
| TCGA-10-0933-01A | 0 | 0.094429 | 0 | 0.16183 | 0 | 0.092164 | 0 | 0.060848 | 0.012573 | 0 | 0 | 0.130252 | 0.064495 | 0 | 0.065851 | 0.248886 | 0 | 0.058909 | 0 | 0 | 0.007089 | 0.002673 | 0.33 | 0.063393 | 1.07239 |
| TCGA-10-0936-01A | 0 | 0 | 0 | 0.156125 | 0 | 0.141184 | 0 | 0.081651 | 0.148689 | 0 | 0 | 0.106086 | 0.006813 | 0 | 0.086996 | 0.197332 | 0 | 0.034019 | 0 | 0.010174 | 0 | 0.03093 | 0.13 | 0.142234 | 1.048554 |
| TCGA-10-0937-01A | 0 | 0.136488 | 0 | 0.19308 | 0 | 0.022052 | 0 | 0.054469 | 0.104705 | 0 | 0 | 0.103537 | 0.022853 | 0.0006 | 0.026096 | 0.263155 | 0 | 0 | 0 | 0.072943 | 0 | 0 | 0.03 | 0.231978 | 0.991939 |
| TCGA-10-0938-01A | 0 | 0.128542 | 0.034683 | 0.157185 | 0 | 0.003187 | 0 | 0.094964 | 0.175848 | 0 | 0 | 0.082131 | 0 | 0.199245 | 0.041217 | 0 | 0 | 0 | 0.013191 | 0 | 0.069808 | 0 | 0.33 | 0.060388 | 1.069123 |
| TCGA-13-0714-01A | 0.005079 | 0.032503 | 0 | 0.112256 | 0 | 0.130581 | 0 | 0.004622 | 0.068301 | 0 | 0 | 0.110704 | 0.022052 | 0.07401 | 0.088184 | 0.251968 | 0.009858 | 0 | 0.089883 | 0 | 0 | 0 | 0.18 | 0.112354 | 1.048199 |
| TCGA-13-0720-01A | 0 | 0.006281 | 0 | 0.07097 | 0 | 0.119533 | 0 | 0.06608 | 0.101938 | 0 | 0 | 0.180003 | 0 | 0.01779 | 0.054495 | 0.044261 | 0 | 0.123478 | 0 | 0.109139 | 0 | 0.106031 | 0.37 | 0.045101 | 1.09237 |
| TCGA-13-0724-01A | 0.060046 | 0 | 0 | 0.261651 | 0 | 0 | 0 | 0.085634 | 0.157692 | 0 | 0 | 0.030847 | 0.019596 | 0.1504 | 0.043218 | 0.12456 | 0.003894 | 0 | 0 | 0.035973 | 0 | 0.026488 | 0.23 | 0.107457 | 1.057541 |
| TCGA-13-0725-01A | 0 | 0.065227 | 0 | 0.086143 | 0 | 0.167604 | 0 | 0.021642 | 0.127115 | 0 | 0.022141 | 0 | 0 | 0 | 0.049408 | 0.334926 | 0 | 0 | 0.064807 | 0 | 0 | 0.060988 | 0.05 | 0.215177 | 0.997265 |
| TCGA-13-0726-01A | 0.003462 | 0 | 0 | 0.067121 | 0 | 0.162453 | 0 | 0 | 0.146585 | 0 | 0 | 0.251463 | 0.013924 | 0.100699 | 0.036747 | 0.054572 | 0.007979 | 0 | 0.138017 | 0 | 0 | 0.016978 | 0.28 | 0.09242 | 1.084954 |
| TCGA-13-0727-01A | 0 | 0.025673 | 0 | 0.074912 | 0 | 0.13042 | 0 | 0.019376 | 0.116347 | 0 | 0 | 0.008054 | 0.015904 | 0.004917 | 0.018013 | 0.33991 | 0.003036 | 0 | 0.21544 | 0 | 0 | 0.027997 | 0.3 | 0.078023 | 1.052043 |
| TCGA-13-0730-01A | 0 | 0.048313 | 0 | 0.277022 | 0 | 0.005977 | 0 | 0.044336 | 0.162266 | 0 | 0 | 0.06662 | 0.024381 | 0.20155 | 0.032363 | 0.130422 | 0 | 0 | 0 | 0.00675 | 0 | 0 | 0.17 | 0.119761 | 1.063725 |
| TCGA-13-0762-01A | 0.071731 | 0 | 0 | 0.010703 | 0 | 0.071798 | 0 | 0 | 0.049944 | 0 | 0 | 0.123055 | 0.00743 | 0.233487 | 0.011618 | 0.337239 | 0 | 0 | 0 | 0.082996 | 0 | 0 | 0.24 | 0.103152 | 1.091507 |
| TCGA-13-0765-01A | 0 | 0.057274 | 0 | 0.052793 | 0 | 0.212463 | 0 | 0.031649 | 0.070333 | 0 | 0 | 0.050049 | 0.008495 | 0.244617 | 0.00333 | 0.174382 | 0 | 0 | 0 | 0.09179 | 0 | 0.002825 | 0.07 | 0.201256 | 1.024247 |
| TCGA-13-0766-01A | 0 | 0 | 0 | 0.12246 | 0 | 0.104006 | 0 | 0.058086 | 0.0738 | 0 | 0 | 0.125737 | 0.009359 | 0 | 0.086884 | 0.151617 | 0 | 0.122112 | 0.026967 | 0 | 0.082081 | 0.036891 | 0.11 | 0.159202 | 1.035091 |
| TCGA-13-0768-01A | 0 | 0.070385 | 0 | 0.260253 | 0 | 0.067014 | 0.017351 | 0.044533 | 0.109824 | 0 | 0 | 0.142499 | 0.007783 | 0 | 0.099662 | 0.151092 | 0.008195 | 0 | 0 | 0.003881 | 0 | 0.017527 | 0 | 0.406052 | 0.920479 |
| TCGA-13-0795-01A | 0 | 0.020446 | 0 | 0.167846 | 0 | 0.131346 | 0 | 0.045 | 0.051284 | 0 | 0 | 0.055613 | 0.040728 | 0 | 0.035256 | 0.375222 | 0 | 0.029364 | 0 | 0.016809 | 0 | 0.031086 | 0.09 | 0.176444 | 1.022729 |
| TCGA-13-0797-01A | 0 | 0.046896 | 0.008968 | 0.172154 | 0 | 0.08556 | 0 | 0.042869 | 0.089898 | 0 | 0 | 0.141261 | 0.00821 | 0.060479 | 0.031911 | 0.15713 | 0 | 0 | 0.14077 | 0 | 0 | 0.013893 | 0.28 | 0.094519 | 1.056143 |
| TCGA-13-0800-01A | 0 | 0.05893 | 0 | 0 | 0 | 0.30546 | 0 | 0.032561 | 0.17636 | 0 | 0 | 0.06901 | 0 | 0.113778 | 0.001709 | 0.014288 | 0 | 0 | 0.198671 | 0 | 0.029233 | 0 | 0.53 | 0.017756 | 1.085108 |
| TCGA-13-0804-01A | 0 | 0.100799 | 0 | 0.104278 | 0 | 0.040272 | 0 | 0.045466 | 0.12014 | 0 | 0.009073 | 0.080371 | 0.003847 | 0 | 0.022394 | 0.333735 | 0.029493 | 0 | 0.00312 | 0 | 0 | 0.107011 | 0.24 | 0.101513 | 1.048109 |
| TCGA-13-0883-01A | 0.028681 | 0 | 0 | 0.083051 | 0 | 0.111943 | 0 | 0.013135 | 0.040106 | 0 | 0 | 0.030708 | 0 | 0.179233 | 0.045085 | 0.379851 | 0.01508 | 0 | 0 | 0.048751 | 0 | 0.024377 | 0.03 | 0.2336 | 1.016106 |
| TCGA-13-0884-01B | 0 | 0.114014 | 0 | 0.168647 | 0 | 0.098347 | 0 | 0.022339 | 0.163986 | 0 | 0 | 0.240319 | 0.009874 | 0.020518 | 0.031784 | 0.096342 | 0.001129 | 0.002556 | 0 | 0 | 0 | 0.030144 | 0.14 | 0.131837 | 1.076797 |
| TCGA-13-0885-01A | 0 | 0.097681 | 0 | 0.151297 | 0 | 0.123081 | 0 | 0.033318 | 0.099693 | 0 | 0 | 0.075343 | 0.004555 | 0 | 0.110634 | 0.24994 | 0.015186 | 0 | 0 | 0.017434 | 0 | 0.021839 | 0 | 0.334961 | 0.948605 |
| TCGA-13-0886-01A | 0 | 0.062108 | 0 | 0.111675 | 0 | 0.07072 | 0 | 0.059487 | 0.123812 | 0 | 0 | 0.070743 | 0 | 0.121761 | 0.096475 | 0.217957 | 0.010687 | 0 | 0.033815 | 0 | 0.001453 | 0.019307 | 0 | 0.351556 | 0.94063 |
| TCGA-13-0887-01A | 0.082256 | 0 | 0 | 0.114719 | 0 | 0.024947 | 0 | 0.048709 | 0.049514 | 0 | 0 | 0.089605 | 0.044736 | 0 | 0 | 0.229782 | 0 | 0.150386 | 0 | 0.13199 | 0.01983 | 0.013526 | 0.33 | 0.062242 | 1.058453 |
| TCGA-13-0888-01A | 0 | 0.003955 | 0 | 0 | 0 | 0.325168 | 0 | 0.032681 | 0.161348 | 0 | 0 | 0.124692 | 0 | 0.210785 | 0.0002 | 0.007661 | 0 | 0 | 0.126006 | 0 | 0.007525 | 0 | 0.42 | 0.03131 | 1.101123 |
| TCGA-13-0891-01A | 0 | 0.092891 | 0.057783 | 0.08065 | 0 | 0.057028 | 0 | 0.058814 | 0.058167 | 0 | 0.043079 | 0.044963 | 0 | 0.228674 | 0.049178 | 0.154301 | 0 | 0.053084 | 0.017892 | 0 | 0 | 0.003495 | 0.25 | 0.099421 | 1.059734 |
| TCGA-13-0893-01B | 0 | 0.061357 | 0.02074 | 0.176356 | 0 | 0.079999 | 0 | 0.04829 | 0.134934 | 0 | 0 | 0.185992 | 0.006244 | 0.041973 | 0.059273 | 0.066489 | 0.00663 | 0 | 0.097116 | 0 | 0 | 0.014608 | 0.07 | 0.204082 | 1.020066 |
| TCGA-13-0897-01A | 0.003962 | 0 | 0 | 0.123528 | 0 | 0.123785 | 0 | 0.03302 | 0.112555 | 0 | 0 | 0.070072 | 0.004214 | 0.006899 | 0.155406 | 0.361039 | 0.00552 | 0 | 0 | 0 | 0 | 0 | 0 | 0.28152 | 0.985893 |
| TCGA-13-0900-01B | 0 | 0.059958 | 0 | 0.074635 | 0 | 0.085097 | 0 | 0.110088 | 0.048947 | 0 | 0 | 0.040133 | 0.005342 | 0 | 0.229159 | 0.301874 | 0.003723 | 0 | 0.041046 | 0 | 0 | 0 | 0 | 0.282872 | 0.99314 |
| TCGA-13-0901-01B | 0.138033 | 0 | 0 | 0.035635 | 0 | 0.056321 | 0 | 0.043424 | 0.02147 | 0 | 0 | 0.080574 | 0.046391 | 0 | 0.004534 | 0.293949 | 0 | 0.188621 | 0 | 0.024638 | 0.045901 | 0.02051 | 0.4 | 0.037853 | 1.074873 |
| TCGA-13-0905-01B | 0 | 0.006007 | 0 | 0.141579 | 0 | 0.117951 | 0 | 0.077631 | 0.180912 | 0 | 0 | 0.052949 | 0 | 0.148333 | 0.048904 | 0.060302 | 0 | 0 | 0 | 0.087688 | 0 | 0.077744 | 0.24 | 0.100554 | 1.059994 |
| TCGA-13-0906-01A | 0.034562 | 0 | 0 | 0.149952 | 0 | 0.134528 | 0 | 0.072517 | 0.089979 | 0 | 0 | 0.113892 | 0.01722 | 0.023832 | 0.193714 | 0.089177 | 0.005675 | 0.011041 | 0 | 0.04108 | 0.022831 | 0 | 0 | 0.260248 | 1.006316 |
| TCGA-13-0908-01B | 0 | 0.007033 | 0 | 0.047692 | 0 | 0.257247 | 0 | 0.066256 | 0.058398 | 0 | 0 | 0.026649 | 0.003836 | 0.080418 | 0.097188 | 0.317844 | 0 | 0 | 0 | 0.022986 | 0 | 0.014452 | 0.09 | 0.190867 | 1.023052 |
| TCGA-13-0911-01A | 0 | 0.057171 | 0 | 0.03744 | 0 | 0 | 0 | 0.001447 | 0.070296 | 0 | 0.032707 | 0 | 0.045116 | 0.29678 | 0.06602 | 0.269035 | 0.0741 | 0 | 0 | 0.049889 | 0 | 0 | 0 | 0.512448 | 0.861137 |
| TCGA-13-0916-01A | 0.005178 | 0 | 0 | 0.14229 | 0 | 0.054858 | 0 | 0.118447 | 0.034719 | 0 | 0 | 0.093864 | 0 | 0.158444 | 0.180456 | 0.198687 | 0 | 0 | 0 | 0.013055 | 0 | 0 | 0 | 0.332938 | 0.967603 |
| TCGA-13-0920-01A | 0 | 0.005337 | 0.051025 | 0.249738 | 0 | 0.087533 | 0 | 0.065839 | 0.051219 | 0 | 0 | 0.104755 | 0.021357 | 0.073274 | 0.088871 | 0.062396 | 0 | 0.002627 | 0.136028 | 0 | 0 | 0 | 0.33 | 0.058649 | 1.080446 |
| TCGA-13-0923-01A | 0 | 0.059421 | 0.120239 | 0.273326 | 0 | 0 | 0 | 0.07273 | 0.126234 | 0 | 0 | 0.11028 | 0.024191 | 0.087647 | 0.069296 | 0.017344 | 0 | 0.001065 | 0 | 0.028476 | 0 | 0.009752 | 0.26 | 0.097049 | 1.061103 |
| TCGA-13-0924-01A | 0.030041 | 0 | 0 | 0.23212 | 0 | 0.08853 | 0 | 0.076558 | 0.059663 | 0 | 0 | 0.073187 | 0.022772 | 0.016164 | 0.146865 | 0.180548 | 0.001 | 0 | 0 | 0.07256 | 0 | 0 | 0.01 | 0.252345 | 0.99841 |
| TCGA-13-1403-01A | 0.027546 | 0 | 0 | 0.03617 | 0 | 0.130288 | 0 | 0 | 0.089235 | 0 | 0 | 0.165035 | 0 | 0 | 0 | 0.198132 | 0 | 0.201201 | 0 | 0.054711 | 0 | 0.097681 | 0.57 | 0.010446 | 1.101099 |
| TCGA-13-1404-01A | 0 | 0.056606 | 0 | 0.067225 | 0 | 0.098812 | 0 | 0.031027 | 0.059394 | 0 | 0 | 0 | 0.032576 | 0.046355 | 0.059253 | 0.432066 | 0.017447 | 0 | 0.099239 | 0 | 0 | 0 | 0.07 | 0.198597 | 1.013162 |
| TCGA-13-1405-01A | 0 | 0 | 0 | 0.283483 | 0 | 0.157064 | 0 | 0.022722 | 0.109397 | 0 | 0 | 0.116373 | 0.034858 | 0 | 0.077569 | 0.195972 | 0 | 0 | 0 | 0 | 0 | 0.002563 | 0.15 | 0.13118 | 1.07127 |
| TCGA-13-1407-01A | 0.128321 | 0 | 0 | 0.129538 | 0 | 0 | 0 | 0.125697 | 0 | 0.003796 | 0 | 0.123225 | 0.002719 | 0.232431 | 0.030475 | 0.14206 | 0 | 0.056661 | 0 | 0.025075 | 0 | 0 | 0.95 | -0.04 | 1.133993 |
| TCGA-13-1408-01A | 0.091762 | 0 | 0.009182 | 0.095017 | 0 | 0.15867 | 0 | 0.015961 | 0.083562 | 0 | 0 | 0.07198 | 0.016294 | 0.08629 | 0.132546 | 0.228321 | 0 | 0 | 0 | 0.010416 | 0 | 0 | 0.09 | 0.187207 | 1.022779 |
| TCGA-13-1409-01A | 0 | 0.035458 | 0.027977 | 0.07938 | 0 | 0.12149 | 0 | 0.079097 | 0.043719 | 0 | 0 | 0.110986 | 0 | 0.251098 | 0 | 0.018118 | 0.0001 | 0 | 0.196547 | 0 | 0 | 0.035993 | 0.33 | 0.058874 | 1.078588 |
| TCGA-13-1410-01A | 0 | 0.085619 | 0 | 0.062719 | 0 | 0.224307 | 0 | 0.087523 | 0.108918 | 0 | 0 | 0.048638 | 0.016343 | 0.08449 | 0.163211 | 0.118232 | 0 | 0 | 0 | 0 | 0 | 0 | 0.03 | 0.234383 | 1.006484 |
| TCGA-13-1411-01A | 0.020918 | 0 | 0 | 0.072581 | 0 | 0.07679 | 0.020333 | 0.014071 | 0 | 0 | 0.048136 | 0 | 0 | 0.125335 | 0.077585 | 0.438816 | 0 | 0 | 0 | 0.078441 | 0 | 0.026994 | 0 | 0.664256 | 0.757182 |
| TCGA-13-1477-01A | 0 | 0.023016 | 0 | 0.036436 | 0 | 0.221573 | 0 | 0.045849 | 0.075329 | 0 | 0 | 0.08476 | 0.01721 | 0.144031 | 0.143535 | 0.158253 | 0 | 0 | 0.050009 | 0 | 0 | 0 | 0.4 | 0.033847 | 1.097333 |
| TCGA-13-1483-01A | 0.008852 | 0 | 0 | 0.050648 | 0 | 0.086937 | 0 | 0.087903 | 0.115745 | 0 | 0 | 0.072479 | 0.007903 | 0.129383 | 0.034683 | 0.277214 | 0 | 0.022709 | 0 | 0.104303 | 0 | 0.00124 | 0.11 | 0.159066 | 1.032059 |
| TCGA-13-1485-01A | 0 | 0.015973 | 0 | 0 | 0 | 0.270242 | 0 | 0.049387 | 0.09636 | 0 | 0 | 0.154297 | 0.017948 | 0.148196 | 0.004609 | 0.101328 | 0.054857 | 0 | 0 | 0.066078 | 0 | 0.020724 | 0.17 | 0.121064 | 1.053049 |
| TCGA-13-1487-01A | 0 | 0.04873 | 0 | 0.13391 | 0 | 0.208075 | 0 | 0.074103 | 0.138443 | 0 | 0 | 0.058224 | 0 | 0.131959 | 0.090808 | 0.074068 | 0 | 0.001711 | 0.019092 | 0 | 0 | 0.020877 | 0.3 | 0.070455 | 1.079844 |
| TCGA-13-1488-01A | 0 | 0.056734 | 0.033953 | 0.171029 | 0 | 0 | 0.045658 | 0.092466 | 0 | 0 | 0 | 0.112852 | 0 | 0.172814 | 0.158621 | 0.093321 | 0 | 0.0007 | 0 | 0.061854 | 0 | 0 | 0 | 0.308377 | 0.972701 |
| TCGA-13-1489-01A | 0 | 0.085556 | 0.013923 | 0.073154 | 0 | 0.181498 | 0 | 0.031854 | 0.182955 | 0 | 0 | 0.035594 | 0.043451 | 0.208045 | 0.007373 | 0.010498 | 0.009298 | 0.018857 | 0.065656 | 0 | 0 | 0.032288 | 0.33 | 0.061354 | 1.066912 |
| TCGA-13-1492-01A | 0.074948 | 0 | 0.078371 | 0.186438 | 0 | 0 | 0 | 0.010651 | 0.070156 | 0 | 0 | 0.069582 | 0.012057 | 0.036763 | 0.040676 | 0.33236 | 0 | 0.017126 | 0.00542 | 0 | 0.048077 | 0.017375 | 0.57 | 0.011168 | 1.080163 |
| TCGA-13-1495-01A | 0 | 0.039066 | 0 | 0.10702 | 0 | 0.235534 | 0 | 0.030369 | 0.101459 | 0.002002 | 0 | 0.087226 | 0 | 0.100292 | 0.167438 | 0.023517 | 0.012264 | 0 | 0 | 0.022523 | 0 | 0.071291 | 0.3 | 0.069839 | 1.095922 |
| TCGA-13-1496-01A | 0 | 0.070555 | 0.055632 | 0.152956 | 0 | 0 | 0.071744 | 0.021809 | 0 | 0 | 0.006784 | 0.112135 | 0.016488 | 0 | 0.099893 | 0.343479 | 0 | 0.002334 | 0.015334 | 0 | 0 | 0.030857 | 0.02 | 0.238665 | 0.993243 |
| TCGA-13-1497-01A | 0 | 0 | 0.002616 | 0.10813 | 0 | 0.172243 | 0 | 0.103592 | 0.032151 | 0 | 0 | 0.036836 | 0.020889 | 0.173275 | 0.130086 | 0.111201 | 0 | 0 | 0 | 0.10898 | 0 | 0 | 0.02 | 0.241921 | 1.002907 |
| TCGA-13-1498-01A | 0 | 0.015048 | 0 | 0.083788 | 0 | 0.143296 | 0 | 0.087374 | 0.039631 | 0 | 0 | 0.112363 | 0.011179 | 0.111704 | 0.093923 | 0.193298 | 0.054661 | 0 | 0 | 0.053414 | 0 | 0.0003 | 0.01 | 0.25719 | 0.987973 |
| TCGA-13-1499-01A | 0.085634 | 0.002742 | 0 | 0.149599 | 0 | 0.19882 | 0.016954 | 0.067214 | 0.058289 | 0 | 0.016871 | 0 | 0.039344 | 0 | 0.180849 | 0.171682 | 0.009407 | 0 | 0 | 0 | 0 | 0.002595 | 0 | 0.479521 | 0.877242 |
| TCGA-13-1501-01A | 0.0029 | 0.004779 | 0.003479 | 0.071037 | 0 | 0.168146 | 0 | 0.027046 | 0.116349 | 0 | 0.001282 | 0.0001 | 0 | 0.089507 | 0.018859 | 0.318368 | 0.001716 | 0 | 0.172771 | 0 | 0 | 0.003698 | 0.3 | 0.075678 | 1.061881 |
| TCGA-13-1505-01A | 0.040533 | 0 | 0.013199 | 0.100923 | 0 | 0.282645 | 0 | 0.095483 | 0.04137 | 0 | 0 | 0.100923 | 0 | 0 | 0.081567 | 0.147331 | 0 | 0.024795 | 0.010136 | 0 | 0 | 0.061095 | 0.18 | 0.114908 | 1.058748 |
| TCGA-13-1506-01A | 0.173995 | 0 | 0 | 0.038676 | 0 | 0.189449 | 0.062624 | 0.023098 | 0.0078 | 0 | 0.024974 | 0.012372 | 0.022176 | 0 | 0.028381 | 0.201619 | 0 | 0.070396 | 0 | 0.116377 | 0 | 0.028062 | 0.3 | 0.087287 | 1.041538 |
| TCGA-13-1507-01A | 0 | 0 | 0.040577 | 0.110504 | 0 | 0.055625 | 0.013014 | 0.09498 | 0.016785 | 0 | 0 | 0.133764 | 0.014889 | 0.036519 | 0.173042 | 0.251188 | 0.022827 | 0 | 0 | 0.00828 | 0.004634 | 0.023372 | 0 | 0.341788 | 0.954417 |
| TCGA-13-1509-01A | 0 | 0.028624 | 0.041178 | 0.187181 | 0 | 0.017407 | 0 | 0.089081 | 0.067663 | 0 | 0 | 0.063303 | 0 | 0 | 0.145495 | 0.276915 | 0.022193 | 0 | 0 | 0.031772 | 0 | 0.029188 | 0.07 | 0.195233 | 1.017655 |
| TCGA-13-1510-01A | 0 | 0.039428 | 0 | 0.153086 | 0 | 0.013654 | 0 | 0.010996 | 0.09845 | 0 | 0 | 0.095224 | 0 | 0.147647 | 0.069534 | 0.184255 | 0.005541 | 0.021351 | 0.160836 | 0 | 0 | 0 | 0.17 | 0.120616 | 1.044495 |
| TCGA-13-1511-01A | 0 | 0 | 0 | 0.04644 | 0 | 0.160121 | 0 | 0.022408 | 0.077317 | 0 | 0 | 0.052402 | 0.005181 | 0.026048 | 0.009726 | 0.43105 | 0 | 0.1139 | 0 | 0.051363 | 0 | 0.004044 | 0.3 | 0.088992 | 1.066707 |
| TCGA-13-1512-01A | 0.002046 | 0 | 0 | 0.06091 | 0 | 0.067565 | 0 | 0.046104 | 0.046824 | 0 | 0 | 0.043298 | 0.0001 | 0.13748 | 0.125176 | 0.397289 | 0.016181 | 0 | 0 | 0.057076 | 0 | 0 | 0.01 | 0.251855 | 1.009275 |
| TCGA-13-2060-01A | 0.003046 | 0 | 0.041964 | 0.136544 | 0 | 0.196603 | 0 | 0.064637 | 0.036456 | 0 | 0 | 0.075714 | 0.006596 | 0 | 0.214343 | 0.205468 | 0 | 0.0001 | 0 | 0.01061 | 0 | 0.007893 | 0 | 0.353823 | 0.95564 |
| TCGA-13-A5FT-01A | 0.055728 | 0 | 0.021759 | 0.035355 | 0 | 0.244372 | 0 | 0.097576 | 0.067942 | 0 | 0.009337 | 0.011536 | 0.019008 | 0.092487 | 0.092116 | 0.051692 | 0 | 0.132737 | 0 | 0.059144 | 0 | 0.009211 | 0.11 | 0.152075 | 1.036717 |
| TCGA-20-0987-01A | 0 | 0.042653 | 0 | 0.080153 | 0 | 0.046765 | 0 | 0.012772 | 0.074082 | 0 | 0.044583 | 0.084634 | 0.071459 | 0.226491 | 0.041692 | 0.262636 | 0 | 0 | 0.012079 | 0 | 0 | 0 | 0.03 | 0.236066 | 1.012135 |
| TCGA-20-0991-01A | 0 | 0 | 0 | 0.236662 | 0 | 0 | 0.108357 | 0.037058 | 0 | 0 | 0 | 0.04184 | 0.007509 | 0.080285 | 0.087871 | 0.354298 | 0 | 0 | 0 | 0.024631 | 0 | 0.021489 | 0 | 0.436054 | 0.900117 |
| TCGA-20-1682-01A | 0.013954 | 0 | 0 | 0.215121 | 0 | 0 | 0.010286 | 0.059548 | 0.039337 | 0.044171 | 0 | 0.09101 | 0.00895 | 0.086828 | 0.11461 | 0.164446 | 0.031566 | 0 | 0.120172 | 0 | 0 | 0 | 0.04 | 0.217935 | 1.009416 |
| TCGA-20-1683-01A | 0.081808 | 0 | 0.249083 | 0.121304 | 0 | 0.122539 | 0 | 0.049127 | 0.016968 | 0 | 0 | 0.045676 | 0 | 0.065799 | 0.05805 | 0.165544 | 0 | 0 | 0.01416 | 0 | 0 | 0.009943 | 0.33 | 0.059683 | 1.044017 |
| TCGA-20-1686-01A | 0 | 0 | 0.0007 | 0.098124 | 0 | 0.166389 | 0 | 0 | 0.114594 | 0 | 0 | 0 | 0.044928 | 0.160374 | 0.090324 | 0.157711 | 0 | 0.011825 | 0.155076 | 0 | 0 | 0 | 0.3 | 0.076003 | 1.064879 |
| TCGA-20-1687-01A | 0.007797 | 0.0002 | 0 | 0.091148 | 0 | 0.169649 | 0 | 0.043984 | 0.061077 | 0 | 0 | 0.063509 | 0.092711 | 0 | 0.056846 | 0.287716 | 0.024045 | 0 | 0 | 0.079612 | 0 | 0.021708 | 0.24 | 0.10365 | 1.045331 |
| TCGA-23-1021-01B | 0.090782 | 0 | 0 | 0.133379 | 0 | 0.117442 | 0 | 0.033622 | 0.049035 | 0 | 0 | 0.118132 | 0.018156 | 0 | 0.002523 | 0.281805 | 0 | 0.089213 | 0 | 0.024746 | 0 | 0.041165 | 0.32 | 0.065719 | 1.066039 |
| TCGA-23-1022-01A | 0 | 0.045847 | 0 | 0.021401 | 0 | 0.085588 | 0 | 0 | 0.062213 | 0 | 0.008009 | 0 | 0 | 0.280292 | 0.031038 | 0.396447 | 0 | 0 | 0.069166 | 0 | 0 | 0 | 0.61 | 0.005008 | 1.164038 |
| TCGA-23-1023-01A | 0 | 0.061621 | 0.011321 | 0.153233 | 0 | 0 | 0 | 0.015158 | 0.086916 | 0 | 0 | 0.09666 | 0.073317 | 0.060109 | 0.078474 | 0.246484 | 0.006732 | 0 | 0.109974 | 0 | 0 | 0 | 0.11 | 0.155153 | 1.022559 |
| TCGA-23-1024-01A | 0.020293 | 0 | 0 | 0.168933 | 0 | 0.126409 | 0 | 0.10617 | 0.054467 | 0 | 0 | 0.076263 | 0.015665 | 0 | 0.050073 | 0.232514 | 0 | 0.08146 | 0 | 0.040652 | 0 | 0.027101 | 0.3 | 0.075006 | 1.066369 |
| TCGA-23-1026-01B | 0.003932 | 0 | 0 | 0.103499 | 0 | 0.182694 | 0 | 0.028743 | 0.088864 | 0 | 0 | 0.172773 | 0.018179 | 0.016935 | 0.105451 | 0.208101 | 0.007552 | 0 | 0 | 0.047399 | 0 | 0.015879 | 0.09 | 0.182066 | 1.032807 |
| TCGA-23-1027-01A | 0 | 0.034083 | 0 | 0.025592 | 0 | 0.125552 | 0 | 0.04739 | 0.075452 | 0 | 0 | 0.077596 | 0.001984 | 0.205953 | 0.00941 | 0.186777 | 0 | 0 | 0 | 0.200842 | 0 | 0.009369 | 0.05 | 0.210174 | 1.018782 |
| TCGA-23-1028-01A | 0 | 0.053586 | 0 | 0.064292 | 0 | 0.23605 | 0 | 0.05764 | 0.021526 | 0 | 0 | 0.089256 | 0.029703 | 0.017298 | 0.15733 | 0.048127 | 0.022077 | 0.006607 | 0 | 0.196507 | 0 | 0 | 0.26 | 0.095622 | 1.068811 |
| TCGA-23-1029-01B | 0.151006 | 0 | 0.092622 | 0.047659 | 0 | 0.152752 | 0 | 0.034085 | 0.029201 | 0 | 0 | 0.071159 | 0.016203 | 0 | 0.040892 | 0.267386 | 0 | 0.034165 | 0 | 0 | 0 | 0.062869 | 0.3 | 0.073636 | 1.046565 |
| TCGA-23-1030-01A | 0 | 0.02316 | 0 | 0.040221 | 0 | 0.060172 | 0 | 0.048318 | 0.082655 | 0 | 0 | 0.078921 | 0 | 0.257066 | 0.032066 | 0.194755 | 0 | 0.04621 | 0 | 0.113747 | 0 | 0.022709 | 0.29 | 0.092086 | 1.083808 |
| TCGA-23-1107-01A | 0 | 0.065202 | 0.07966 | 0.101213 | 0 | 0.009219 | 0 | 0.048708 | 0.086242 | 0.015134 | 0 | 0.036478 | 0.030252 | 0.267191 | 0.013017 | 0.23241 | 0.005725 | 0 | 0.009549 | 0 | 0 | 0 | 0.09 | 0.178055 | 1.036089 |
| TCGA-23-1109-01A | 0 | 0.050638 | 0 | 0.072781 | 0 | 0.100539 | 0 | 0.026061 | 0.107855 | 0 | 0.052946 | 0 | 0.031313 | 0.241573 | 0.041291 | 0.197784 | 0.05853 | 0 | 0 | 0.01869 | 0 | 0 | 0 | 0.36219 | 0.941365 |
| TCGA-23-1110-01A | 0.184046 | 0 | 0.285661 | 0.01817 | 0 | 0.144431 | 0 | 0 | 0.031675 | 0 | 0.02095 | 0 | 0.04561 | 0.148609 | 0.0105 | 0.070471 | 0 | 0.022702 | 0.017177 | 0 | 0 | 0 | 0.36 | 0.050705 | 1.04629 |
| TCGA-23-1111-01A | 0.091119 | 0 | 0 | 0.018437 | 0 | 0.268492 | 0 | 0 | 0.0712 | 0 | 0 | 0.137069 | 0.047099 | 0.014419 | 0.012399 | 0.277615 | 0 | 0.031251 | 0 | 0 | 0 | 0.030901 | 0.38 | 0.042971 | 1.07757 |
| TCGA-23-1113-01A | 0 | 0.129045 | 0 | 0.033948 | 0 | 0.128716 | 0 | 0.034452 | 0.092834 | 0 | 0 | 0.025142 | 0.014798 | 0.080677 | 0.006571 | 0.269385 | 0.005637 | 0 | 0.168275 | 0 | 0 | 0.010521 | 0.51 | 0.02305 | 1.069204 |
| TCGA-23-1114-01B | 0 | 0.002287 | 0.003312 | 0.020223 | 0 | 0.36405 | 0 | 0.063709 | 0.039823 | 0 | 0 | 0.185912 | 0.006432 | 0.091058 | 0.001434 | 0.00997 | 0.008876 | 0 | 0 | 0.202915 | 0 | 0 | 0.54 | 0.015426 | 1.125604 |
| TCGA-23-1116-01A | 0.012608 | 0 | 0 | 0.008451 | 0 | 0.075879 | 0 | 0.028575 | 0.025675 | 0 | 0 | 0.031467 | 0.0003 | 0.160506 | 0.071369 | 0.346813 | 0.022808 | 0 | 0 | 0.171064 | 0.0002 | 0.044265 | 0 | 0.492278 | 0.870501 |
| TCGA-23-1118-01A | 0 | 0.01499 | 0 | 0.11111 | 0 | 0.173583 | 0 | 0.056567 | 0.00128 | 0 | 0 | 0.057415 | 0.012345 | 0.028402 | 0.159149 | 0.305564 | 0.019376 | 0.006825 | 0 | 0.037864 | 0 | 0.015529 | 0 | 0.262752 | 0.992999 |
| TCGA-23-1119-01A | 0 | 0.051967 | 0 | 0.110693 | 0 | 0.062723 | 0.008097 | 0.010355 | 0.028862 | 0.025361 | 0 | 0.067105 | 0.00246 | 0 | 0.169977 | 0.280424 | 0.016679 | 0 | 0.152297 | 0 | 0 | 0.012999 | 0.04 | 0.218096 | 1.008946 |
| TCGA-23-1120-01A | 0 | 0.0005 | 0 | 0.107082 | 0 | 0.074184 | 0.036412 | 0.043686 | 0.065104 | 0 | 0 | 0.112064 | 0.018085 | 0.122102 | 0.177397 | 0.23885 | 0 | 0 | 0 | 0 | 0 | 0.004582 | 0 | 0.376922 | 0.940018 |
| TCGA-23-1122-01A | 0 | 0.035365 | 0 | 0.037643 | 0 | 0.159826 | 0 | 0.033344 | 0.078186 | 0 | 0 | 0.050037 | 0.008211 | 0.119757 | 0.08568 | 0.239915 | 0.054965 | 0 | 0.097071 | 0 | 0 | 0 | 0.18 | 0.114866 | 1.04391 |
| TCGA-23-1123-01A | 0 | 0.028197 | 0 | 0.192348 | 0 | 0 | 0 | 0.094576 | 0.052239 | 0 | 0 | 0.086198 | 0 | 0.022806 | 0.110716 | 0.35919 | 0.007689 | 0 | 0 | 0.015442 | 0 | 0.030598 | 0 | 0.270207 | 0.985595 |
| TCGA-23-1809-01A | 0.139687 | 0 | 0 | 0.037369 | 0 | 0.067385 | 0 | 0.071402 | 0.042489 | 0 | 0 | 0.039598 | 0.0005 | 0.384866 | 0.055164 | 0.15541 | 0 | 0.002074 | 0 | 0.004073 | 0 | 0 | 0.3 | 0.073791 | 1.12191 |
| TCGA-23-2077-01A | 0 | 0.013829 | 0 | 0.060846 | 0 | 0.156754 | 0 | 0.033501 | 0.075136 | 0 | 0 | 0.036763 | 0.011308 | 0.035456 | 0.050458 | 0.401981 | 0.018884 | 0 | 0.089097 | 0 | 0 | 0.015987 | 0.15 | 0.12754 | 1.04093 |
| TCGA-23-2078-01A | 0.098012 | 0 | 0 | 0.13478 | 0 | 0.211552 | 0 | 0.043705 | 0.08707 | 0 | 0 | 0.069343 | 0.010555 | 0.004046 | 0.210952 | 0.12493 | 0.005057 | 0 | 0 | 0 | 0 | 0 | 0 | 0.335882 | 0.967466 |
| TCGA-23-2084-01A | 0 | 0.0001 | 0 | 0.03288 | 0 | 0.188788 | 0 | 0.035801 | 0.088196 | 0 | 0 | 0.111476 | 0.013431 | 0 | 0.022337 | 0.390364 | 0 | 0.030662 | 0 | 0.011535 | 0 | 0.074405 | 0.23 | 0.105073 | 1.055659 |
| TCGA-24-0966-01A | 0.002743 | 0 | 0 | 0.171486 | 0 | 0.025901 | 0 | 0.014374 | 0.098975 | 0 | 0 | 0.081741 | 0.044749 | 0 | 0.014004 | 0.433945 | 0.0009 | 0.074892 | 0 | 0.028195 | 0 | 0.008098 | 0.12 | 0.143796 | 1.041359 |
| TCGA-24-0968-01A | 0.0008 | 0 | 0 | 0.154096 | 0 | 0.0006 | 0 | 0.088686 | 0.099119 | 0 | 0 | 0.130891 | 0.016434 | 0 | 0.085576 | 0.37881 | 0 | 0 | 0 | 0.026518 | 0 | 0.018538 | 0.03 | 0.235598 | 1.002267 |
| TCGA-24-0970-01B | 0.093845 | 0 | 0 | 0.018496 | 0 | 0.154231 | 0 | 0.011724 | 0.05831 | 0 | 0 | 0.039073 | 0 | 0.135773 | 0.007256 | 0.429613 | 0 | 0.026618 | 0 | 0.020603 | 0 | 0.004457 | 0.3 | 0.075671 | 1.087364 |
| TCGA-24-0979-01A | 0 | 0.03758 | 0 | 0.194347 | 0 | 0.127618 | 0 | 0.049731 | 0.103537 | 0 | 0 | 0.0631 | 0.02285 | 0.112396 | 0.071028 | 0.006771 | 0.0007 | 0 | 0.186634 | 0 | 0.023756 | 0 | 0.21 | 0.107799 | 1.050681 |
| TCGA-24-0982-01A | 0.035191 | 0 | 0.036523 | 0.130104 | 0 | 0.219899 | 0 | 0.075112 | 0.039703 | 0 | 0 | 0.083229 | 0.011053 | 0.083746 | 0.071883 | 0.188692 | 0 | 0.014121 | 0 | 0.003548 | 0 | 0.007196 | 0.23 | 0.106788 | 1.056758 |
| TCGA-24-1103-01A | 0.0009 | 0 | 0.012702 | 0.22671 | 0 | 0.082427 | 0 | 0.079907 | 0.09137 | 0 | 0 | 0.146673 | 0.032886 | 0.202757 | 0.071196 | 0 | 0 | 0 | 0.004806 | 0 | 0.04771 | 0 | 0.35 | 0.052131 | 1.108718 |
| TCGA-24-1104-01A | 0 | 0.0002 | 0 | 0.168672 | 0 | 0.20179 | 0 | 0.034746 | 0.140451 | 0 | 0 | 0.137952 | 0.022412 | 0 | 0.057325 | 0.186059 | 0 | 0 | 0 | 0.034512 | 0 | 0.015919 | 0.03 | 0.23301 | 1.009115 |
| TCGA-24-1105-01A | 0.042876 | 0 | 0 | 0.1007 | 0 | 0.13844 | 0.046863 | 0.044922 | 0.008328 | 0 | 0 | 0.081535 | 0 | 0.058523 | 0.085065 | 0.375002 | 0 | 0 | 0 | 0.01461 | 0 | 0.003135 | 0.32 | 0.064637 | 1.078469 |
| TCGA-24-1413-01A | 0 | 0 | 0.009433 | 0.048877 | 0 | 0.183673 | 0 | 0.052777 | 0.048017 | 0 | 0 | 0.098532 | 0.01226 | 0.041921 | 0.041163 | 0.300214 | 0 | 0.039688 | 0.003988 | 0 | 0.070156 | 0.049301 | 0.3 | 0.090179 | 1.056118 |
| TCGA-24-1416-01A | 0 | 0.003325 | 0.037316 | 0.082437 | 0 | 0.178757 | 0 | 0.007398 | 0.096548 | 0 | 0 | 0.162104 | 0.024181 | 0 | 0.006571 | 0.203523 | 0 | 0.052881 | 0 | 0.095376 | 0.015832 | 0.03375 | 0.33 | 0.061942 | 1.068424 |
| TCGA-24-1417-01A | 0.00855 | 0 | 0.010126 | 0.110837 | 0 | 0.051209 | 0 | 0.038346 | 0.130382 | 0 | 0 | 0.102948 | 0 | 0.125487 | 0.06774 | 0.331052 | 0 | 0 | 0 | 0.022262 | 0 | 0.001059 | 0 | 0.274915 | 0.984578 |
| TCGA-24-1418-01A | 0.057282 | 0 | 0 | 0.067057 | 0 | 0.294149 | 0 | 0.017745 | 0.056951 | 0 | 0 | 0.079505 | 0.029909 | 0 | 0.079014 | 0.198039 | 0 | 0.073314 | 0 | 0.017514 | 0.008053 | 0.021469 | 0.3 | 0.083961 | 1.063208 |
| TCGA-24-1419-01A | 0 | 0.003802 | 0.068359 | 0.048821 | 0 | 0.139394 | 0 | 0 | 0.151792 | 0 | 0 | 0.081258 | 0.098178 | 0 | 0 | 0.231653 | 0 | 0.043168 | 0 | 0.057672 | 0.040521 | 0.035382 | 0.4 | 0.039581 | 1.058162 |
| TCGA-24-1422-01A | 0 | 0.007571 | 0 | 0.069099 | 0 | 0.157768 | 0 | 0.016443 | 0.131696 | 0 | 0 | 0.20535 | 0.045506 | 0.009371 | 0.033858 | 0.281591 | 0.002549 | 0.0006 | 0 | 0.022294 | 0 | 0.01633 | 0.05 | 0.213429 | 1.01595 |
| TCGA-24-1423-01A | 0 | 0.049988 | 0 | 0.100073 | 0 | 0.142199 | 0 | 0.095026 | 0.094504 | 0 | 0 | 0.116716 | 0.014124 | 0.140325 | 0.089379 | 0.068726 | 0.003089 | 0 | 0.076762 | 0 | 0.005684 | 0.003404 | 0.23 | 0.104178 | 1.055967 |
| TCGA-24-1424-01A | 0 | 0.08419 | 0 | 0.105186 | 0 | 0.101444 | 0 | 0.049232 | 0.065022 | 0 | 0 | 0.080615 | 0.022527 | 0.113495 | 0.104733 | 0.195404 | 0 | 0 | 0.078152 | 0 | 0 | 0 | 0.17 | 0.119344 | 1.043067 |
| TCGA-24-1425-01A | 0.083242 | 0 | 0 | 0.056378 | 0 | 0.074803 | 0 | 0 | 0.14842 | 0 | 0 | 0.072861 | 0 | 0.177701 | 0.064311 | 0.271112 | 0 | 0 | 0 | 0.020802 | 0 | 0.03037 | 0.09 | 0.188317 | 1.025164 |
| TCGA-24-1426-01A | 0 | 0.004167 | 0 | 0 | 0 | 0.242023 | 0 | 0.004944 | 0.151413 | 0 | 0.082068 | 0 | 0.020118 | 0.035246 | 0.011516 | 0.218306 | 0 | 0.003938 | 0.19211 | 0 | 0 | 0.034151 | 0.73 | -0.0117 | 1.086789 |
| TCGA-24-1427-01A | 0.003281 | 0.004042 | 0 | 0.213571 | 0 | 0.176642 | 0 | 0.016264 | 0.106634 | 0 | 0 | 0.049576 | 0.010531 | 0 | 0.056882 | 0.310954 | 0.005153 | 0 | 0.010338 | 0 | 0 | 0.036133 | 0.14 | 0.135823 | 1.047167 |
| TCGA-24-1428-01A | 0 | 0.0004 | 0 | 0.029271 | 0 | 0.055395 | 0 | 0.021368 | 0.113223 | 0 | 0 | 0.0593 | 0.002646 | 0.244994 | 0.057582 | 0.308976 | 0 | 0 | 0 | 0.106854 | 0 | 0 | 0 | 0.519317 | 0.854018 |
| TCGA-24-1430-01A | 0.055036 | 0 | 0 | 0 | 0 | 0.214815 | 0 | 0.059106 | 0.113142 | 0 | 0 | 0.014115 | 0 | 0.072176 | 0.027751 | 0.343987 | 0 | 0 | 0.013157 | 0 | 0 | 0.086715 | 0.57 | 0.010575 | 1.096311 |
| TCGA-24-1431-01A | 0.0006 | 0.002834 | 0 | 0.140436 | 0 | 0.112113 | 0 | 0.029005 | 0.087631 | 0 | 0 | 0.080611 | 0.003849 | 0 | 0.07005 | 0.407911 | 0 | 0 | 0 | 0.01805 | 0 | 0.046916 | 0.4 | 0.037398 | 1.089906 |
| TCGA-24-1434-01A | 0 | 0 | 0 | 0.046701 | 0 | 0.220991 | 0 | 0.037367 | 0.033881 | 0 | 0 | 0.046944 | 0.072952 | 0 | 0.104921 | 0.312906 | 0 | 0 | 0 | 0.090146 | 0.012242 | 0.020947 | 0.01 | 0.24674 | 0.988627 |
| TCGA-24-1435-01A | 0.00452 | 0 | 0 | 0.102474 | 0 | 0.126076 | 0 | 0.041321 | 0.116652 | 0 | 0 | 0.049161 | 0.016577 | 0.070431 | 0.141918 | 0.207815 | 0.038078 | 0 | 0 | 0.084977 | 0 | 0 | 0 | 0.271024 | 0.982313 |
| TCGA-24-1464-01A | 0.004664 | 0.008412 | 0 | 0.359497 | 0 | 0.04409 | 0 | 0.07934 | 0.066313 | 0 | 0 | 0.116088 | 0.017728 | 0 | 0.043801 | 0.237309 | 0 | 0.005565 | 0 | 0 | 0.0007 | 0.016524 | 0 | 0.272387 | 0.99763 |
| TCGA-24-1467-01A | 0.0003 | 0 | 0 | 0.169438 | 0 | 0.155248 | 0 | 0.04755 | 0.119076 | 0 | 0 | 0.082996 | 0.036358 | 0.10863 | 0.079529 | 0.124992 | 0 | 0 | 0.075881 | 0 | 0 | 0 | 0.18 | 0.116948 | 1.052733 |
| TCGA-24-1469-01A | 0.013096 | 0 | 0 | 0.029115 | 0 | 0.135941 | 0 | 0.065995 | 0.044366 | 0 | 0 | 0.092438 | 0 | 0.255228 | 0.101284 | 0.227182 | 0 | 0 | 0 | 0.035354 | 0 | 0 | 0.01 | 0.25649 | 1.007009 |
| TCGA-24-1470-01A | 0.010949 | 0 | 0.022814 | 0.172892 | 0 | 0.01096 | 0 | 0.108338 | 0.163135 | 0 | 0 | 0.07168 | 0.015853 | 0.077892 | 0.13542 | 0.130609 | 0.027274 | 0 | 0 | 0.052183 | 0 | 0 | 0.01 | 0.244244 | 0.995789 |
| TCGA-24-1471-01A | 0 | 0.051867 | 0 | 0.106163 | 0 | 0.165938 | 0 | 0.062304 | 0.111193 | 0 | 0 | 0.052354 | 0.010056 | 0 | 0.008924 | 0.333686 | 0 | 0.01163 | 0 | 0.047314 | 0 | 0.038572 | 0.53 | 0.019936 | 1.08256 |
| TCGA-24-1474-01A | 0 | 0.016337 | 0 | 0 | 0 | 0.164366 | 0 | 0.016717 | 0.035255 | 0 | 0 | 0.171613 | 0.017872 | 0 | 0.036561 | 0.378644 | 0.0003 | 0 | 0.130939 | 0 | 0 | 0.031401 | 0.33 | 0.058017 | 1.072897 |
| TCGA-24-1544-01A | 0.001959 | 0 | 0.015302 | 0.336961 | 0 | 0 | 0 | 0.036134 | 0.07033 | 0 | 0 | 0.059034 | 0 | 0.222183 | 0.024853 | 0 | 0.004453 | 0 | 0.203594 | 0 | 0 | 0.025198 | 0.3 | 0.073421 | 1.084214 |
| TCGA-24-1546-01A | 0.003414 | 0.015627 | 0.026053 | 0.030178 | 0 | 0.128839 | 0 | 0.019142 | 0.088034 | 0 | 0 | 0.052644 | 0.010274 | 0.176178 | 0.018929 | 0.249137 | 0.046356 | 0.002934 | 0 | 0.107139 | 0.002787 | 0.022336 | 0.06 | 0.208907 | 1.012609 |
| TCGA-24-1549-01A | 0 | 0 | 0 | 0.094422 | 0 | 0.098115 | 0.052036 | 0.027397 | 0.150722 | 0 | 0.026971 | 0.171813 | 0.001194 | 0.141454 | 0.103204 | 0.129476 | 0 | 0 | 0 | 0 | 0 | 0.003197 | 0.01 | 0.244562 | 1.01142 |
| TCGA-24-1550-01A | 0.042207 | 0 | 0 | 0.033313 | 0 | 0.094307 | 0 | 0.048975 | 0.016592 | 0 | 0 | 0.058508 | 0 | 0.196475 | 0.137126 | 0.330488 | 0 | 0 | 0 | 0.041488 | 0.0005 | 0 | 0 | 0.609612 | 0.793242 |
| TCGA-24-1551-01A | 0.009844 | 0 | 0 | 0.105901 | 0 | 0.086725 | 0 | 0.045751 | 0.100446 | 0 | 0 | 0.035965 | 0.05593 | 0.145753 | 0.101239 | 0.208345 | 0.009476 | 0 | 0 | 0.094626 | 0 | 0 | 0 | 0.427421 | 0.903752 |
| TCGA-24-1552-01A | 0 | 0.016774 | 0.016845 | 0.137258 | 0 | 0.127846 | 0 | 0.059797 | 0.183587 | 0 | 0 | 0 | 0.025796 | 0.252202 | 0.004489 | 0.083416 | 0 | 0 | 0.09199 | 0 | 0 | 0 | 0.26 | 0.097097 | 1.067003 |
| TCGA-24-1553-01A | 0 | 0.049345 | 0 | 0.328561 | 0 | 0.050913 | 0 | 0.061506 | 0.106634 | 0 | 0 | 0.104821 | 0.001012 | 0.007565 | 0.07493 | 0.136911 | 0.008475 | 0 | 0.03459 | 0 | 0 | 0.034738 | 0 | 0.295904 | 0.979615 |
| TCGA-24-1557-01A | 0.001766 | 0 | 0 | 0.016671 | 0 | 0.205721 | 0 | 0.053154 | 0.104507 | 0 | 0 | 0.027405 | 0.05186 | 0.155039 | 0.084271 | 0.157538 | 0 | 0 | 0.142069 | 0 | 0 | 0 | 0.12 | 0.143049 | 1.032572 |
| TCGA-24-1558-01A | 0 | 0.064741 | 0.013247 | 0 | 0 | 0.084915 | 0 | 0.014993 | 0.071168 | 0.001 | 0 | 0.013574 | 0 | 0.497744 | 0.012939 | 0.19545 | 0 | 0 | 0.030275 | 0 | 0 | 0 | 0.03 | 0.230558 | 1.081645 |
| TCGA-24-1560-01A | 0.068143 | 0 | 0 | 0.082377 | 0 | 0.159182 | 0 | 0.100655 | 0.016441 | 0 | 0 | 0.137547 | 0.005257 | 0 | 0.058226 | 0.163858 | 0 | 0.129208 | 0 | 0 | 0.047123 | 0.031985 | 0.36 | 0.04858 | 1.081327 |
| TCGA-24-1562-01A | 0 | 0.058787 | 0 | 0.099654 | 0 | 0.131299 | 0 | 0.038708 | 0.133091 | 0 | 0.004845 | 0.002089 | 0 | 0.197911 | 0.00925 | 0.215184 | 0.0069 | 0 | 0 | 0.075892 | 0 | 0.026391 | 0.09 | 0.182212 | 1.027251 |
| TCGA-24-1563-01A | 0.014693 | 0 | 0 | 0.029861 | 0 | 0.206414 | 0 | 0.028909 | 0.023245 | 0 | 0 | 0.078599 | 0.001301 | 0.045838 | 0.026965 | 0.244779 | 0 | 0.006453 | 0 | 0.176178 | 0.070679 | 0.046086 | 0.3 | 0.069858 | 1.061161 |
| TCGA-24-1565-01A | 0 | 0 | 0.09815 | 0.170722 | 0 | 0.075557 | 0 | 0.025273 | 0.135167 | 0 | 0 | 0.116266 | 0.006992 | 0 | 0.004904 | 0.282861 | 0 | 0.01077 | 0 | 0.009246 | 0 | 0.064091 | 0.11 | 0.152639 | 1.028385 |
| TCGA-24-1567-01A | 0.160174 | 0 | 0 | 0.167758 | 0 | 0 | 0 | 0.034617 | 0.049207 | 0 | 0 | 0.045134 | 0.004773 | 0.115413 | 0.005032 | 0.368358 | 0 | 0.007754 | 0 | 0.041779 | 0 | 0 | 0.42 | 0.029795 | 1.094984 |
| TCGA-24-1603-01A | 0 | 0.04367 | 0.002689 | 0.067415 | 0 | 0.182155 | 0 | 0.036403 | 0.095959 | 0 | 0 | 0.090824 | 0 | 0.156662 | 0.095729 | 0.055696 | 0.001456 | 0 | 0.102755 | 0 | 0 | 0.068586 | 0.27 | 0.095111 | 1.056154 |
| TCGA-24-1604-01A | 0.031187 | 0 | 0.001325 | 0.063543 | 0 | 0.224433 | 0 | 0.055774 | 0.041132 | 0 | 0 | 0.086023 | 0.054563 | 0 | 0.051929 | 0.275684 | 0 | 0.047343 | 0 | 0.017684 | 0 | 0.049381 | 0.3 | 0.088961 | 1.05499 |
| TCGA-24-1616-01A | 0 | 0.001265 | 0 | 0.033763 | 0 | 0.204875 | 0 | 0.029482 | 0.103908 | 0 | 0 | 0.159411 | 0.024616 | 0.081445 | 0.079753 | 0.132393 | 0 | 0 | 0 | 0.135904 | 0 | 0.013185 | 0.36 | 0.047341 | 1.084421 |
| TCGA-24-1842-01A | 0.019336 | 0 | 0 | 0.212235 | 0 | 0 | 0 | 0.039109 | 0.099835 | 0 | 0 | 0.119219 | 0.024357 | 0.162764 | 0.138183 | 0.159537 | 0.00948 | 0 | 0.01536 | 0 | 0 | 0.0006 | 0 | 0.3219 | 0.968036 |
| TCGA-24-1843-01A | 0 | 0.091399 | 0.044047 | 0.112595 | 0 | 0.039223 | 0 | 0.030507 | 0.077809 | 0 | 0.03041 | 0 | 0 | 0.225055 | 0.046206 | 0.261167 | 0 | 0 | 0 | 0.041583 | 0 | 0 | 0 | 0.519753 | 0.8534 |
| TCGA-24-1844-01A | 0 | 0 | 0.071499 | 0.002709 | 0 | 0.186457 | 0 | 0.001879 | 0.043768 | 0 | 0 | 0.163769 | 0.035162 | 0 | 0.014336 | 0.375721 | 0 | 0 | 0.055106 | 0 | 0.049594 | 0 | 0.53 | 0.017736 | 1.084042 |
| TCGA-24-1845-01A | 0 | 0.070717 | 0 | 0.021019 | 0 | 0.099894 | 0 | 0.045014 | 0.064216 | 0 | 0 | 0.03167 | 0 | 0.156886 | 0.073813 | 0.292308 | 0 | 0.003163 | 0 | 0.129215 | 0 | 0.012084 | 0.11 | 0.162284 | 1.037447 |
| TCGA-24-1846-01A | 0.04271 | 0 | 0 | 0.067858 | 0 | 0 | 0 | 0.064373 | 0.065717 | 0 | 0 | 0.088002 | 0 | 0.290925 | 0.086507 | 0.255348 | 0 | 0 | 0 | 0.03856 | 0 | 0 | 0.03 | 0.2319 | 1.02832 |
| TCGA-24-1847-01A | 0.011184 | 0 | 0.02445 | 0.261925 | 0 | 0.083004 | 0 | 0.062932 | 0.120423 | 0 | 0 | 0.120884 | 0.023709 | 0.042155 | 0.072457 | 0.129512 | 0.011063 | 0 | 0.017239 | 0 | 0 | 0.019064 | 0 | 0.360381 | 0.942879 |
| TCGA-24-1850-01A | 0.001359 | 0 | 0 | 0.147587 | 0 | 0.11875 | 0 | 0.067656 | 0.058724 | 0 | 0 | 0.051532 | 0.001312 | 0.014105 | 0.138389 | 0.318792 | 0.001606 | 0 | 0 | 0.066334 | 0 | 0.013853 | 0.11 | 0.152524 | 1.04207 |
| TCGA-24-1923-01A | 0.065182 | 0 | 0 | 0.042825 | 0 | 0.111641 | 0 | 0.023246 | 0.019138 | 0 | 0 | 0.098194 | 0 | 0 | 0.028131 | 0.365078 | 0 | 0.074056 | 0 | 0.042604 | 0.01148 | 0.118424 | 0.53 | 0.017662 | 1.087805 |
| TCGA-24-1924-01A | 0 | 0.085798 | 0 | 0.222973 | 0 | 0 | 0 | 0.108348 | 0.04107 | 0 | 0 | 0.105625 | 0.017849 | 0 | 0.183204 | 0.1848 | 0.015031 | 0 | 0 | 0.010208 | 0.00445 | 0.020643 | 0 | 0.340109 | 0.958007 |
| TCGA-24-1928-01A | 0.016454 | 0 | 0 | 0.121941 | 0 | 0.268277 | 0 | 0.045087 | 0.025874 | 0 | 0 | 0.047858 | 0.0467 | 0 | 0.112555 | 0.301519 | 0 | 0 | 0 | 0.004823 | 0 | 0.00891 | 0.14 | 0.131372 | 1.051235 |
| TCGA-24-1930-01A | 0 | 0 | 0.01807 | 0.071592 | 0 | 0.183296 | 0 | 0.049046 | 0.035121 | 0 | 0 | 0.047364 | 0 | 0.059974 | 0.124835 | 0.363916 | 0 | 0 | 0 | 0.023264 | 0 | 0.023521 | 0.04 | 0.222801 | 1.0105 |
| TCGA-24-2020-01A | 0.053008 | 0 | 0 | 0.116858 | 0 | 0.054425 | 0 | 0.015644 | 0.079401 | 0 | 0 | 0.034942 | 0.024743 | 0.218587 | 0.0928 | 0.270532 | 0 | 0 | 0 | 0.039059 | 0 | 0 | 0.24 | 0.101818 | 1.079131 |
| TCGA-24-2023-01A | 0.0008 | 0 | 0 | 0.077056 | 0 | 0.216247 | 0 | 0.03068 | 0.054603 | 0 | 0 | 0.145681 | 0.009885 | 0 | 0.074434 | 0.291469 | 0 | 0.0003 | 0 | 0.081622 | 0 | 0.017219 | 0.16 | 0.123429 | 1.05079 |
| TCGA-24-2024-01A | 0.023721 | 0 | 0.017127 | 0.066033 | 0 | 0.103301 | 0 | 0.092111 | 0.031335 | 0 | 0 | 0.131562 | 0 | 0.087509 | 0.065764 | 0.196681 | 0.003757 | 0 | 0 | 0.105313 | 0 | 0.075787 | 0.35 | 0.052233 | 1.074406 |
| TCGA-24-2026-01A | 0 | 0.033254 | 0.047459 | 0.118357 | 0 | 0.057597 | 0 | 0.079154 | 0.093276 | 0 | 0 | 0.058263 | 0.013535 | 0.052199 | 0.167205 | 0.120375 | 0.012688 | 0 | 0 | 0.118393 | 0 | 0.028244 | 0 | 0.263138 | 0.984986 |
| TCGA-24-2027-01A | 0 | 0.002867 | 0 | 0.058947 | 0 | 0.254466 | 0 | 0.035419 | 0.174478 | 0 | 0 | 0.089667 | 0.036281 | 0.100709 | 0.044472 | 0.023052 | 0 | 0 | 0.179642 | 0 | 0 | 0 | 0.64 | -0.0006 | 1.096655 |
| TCGA-24-2033-01A | 0.061402 | 0 | 0 | 0.092763 | 0 | 0.079549 | 0 | 0.037485 | 0.056875 | 0 | 0 | 0.122062 | 0 | 0 | 0.062224 | 0.271332 | 0.0003 | 0 | 0 | 0.136997 | 0.011071 | 0.067913 | 0.42 | 0.028091 | 1.078702 |
| TCGA-24-2035-01A | 0 | 0 | 0 | 0.08272 | 0 | 0.027052 | 0.004327 | 0.043348 | 0.054782 | 0 | 0 | 0.020294 | 0.049358 | 0 | 0.127025 | 0.433673 | 0.029806 | 0 | 0.105445 | 0 | 0 | 0.022169 | 0 | 0.279855 | 0.979978 |
| TCGA-24-2036-01A | 0 | 0.005665 | 0.015207 | 0 | 0 | 0.424917 | 0 | 0.007223 | 0.11195 | 0 | 0.047705 | 0.00294 | 0.018712 | 0.175463 | 0.006935 | 0.040179 | 0.01231 | 0 | 0.130793 | 0 | 0 | 0 | 0.24 | 0.10307 | 1.061473 |
| TCGA-24-2038-01A | 0 | 0.003 | 0.0004 | 0.038751 | 0 | 0.197027 | 0 | 0.002139 | 0.04589 | 0 | 0 | 0.157469 | 0.050919 | 0 | 0.001189 | 0.277191 | 0.047319 | 0 | 0 | 0.062854 | 0 | 0.115821 | 0.74 | -0.0152 | 1.107622 |
| TCGA-24-2254-01A | 0.004478 | 0 | 0 | 0.003534 | 0 | 0.123162 | 0 | 0.023709 | 0.029433 | 0 | 0 | 0.0226 | 0.036919 | 0.21134 | 0.003277 | 0.292111 | 0 | 0 | 0 | 0.249437 | 0 | 0 | 0.11 | 0.162275 | 1.065403 |
| TCGA-24-2261-01A | 0 | 0.027459 | 0 | 0.232869 | 0 | 0.131618 | 0 | 0.083189 | 0.088188 | 0 | 0 | 0.060549 | 0.015991 | 0 | 0.148315 | 0.173572 | 0.008321 | 0 | 0.029928 | 0 | 0 | 0 | 0.03 | 0.231528 | 1.012592 |
| TCGA-24-2262-01A | 0 | 0.006183 | 0.034544 | 0.148548 | 0 | 0.088934 | 0 | 0.069336 | 0.122485 | 0 | 0.054841 | 0 | 0.011039 | 0.130622 | 0.109552 | 0.071482 | 0.031308 | 0 | 0 | 0.100153 | 0 | 0.020972 | 0.06 | 0.208706 | 1.008377 |
| TCGA-24-2267-01A | 0 | 0.023134 | 0 | 0.128413 | 0 | 0.086377 | 0 | 0.079711 | 0.122565 | 0 | 0 | 0.01228 | 0.004267 | 0.199246 | 0.124228 | 0.136939 | 0 | 0 | 0 | 0.063366 | 0 | 0.019474 | 0 | 0.315007 | 0.966065 |
| TCGA-24-2271-01A | 0.025047 | 0 | 0 | 0.113502 | 0 | 0.225119 | 0 | 0.097054 | 0.113968 | 0 | 0 | 0.089452 | 0.027275 | 0.092984 | 0.132192 | 0.042698 | 0.001895 | 0 | 0.029298 | 0 | 0 | 0.009516 | 0.15 | 0.125847 | 1.062572 |
| TCGA-24-2280-01A | 0.021155 | 0 | 0.033752 | 0.092515 | 0 | 0.093116 | 0 | 0.063893 | 0.048646 | 0.011824 | 0 | 0.100639 | 0.021558 | 0 | 0.01891 | 0.317832 | 0 | 0 | 0.156425 | 0 | 0 | 0.019733 | 0.38 | 0.044137 | 1.064135 |
| TCGA-24-2281-01A | 0.014008 | 0 | 0 | 0.0827 | 0 | 0 | 0 | 0.051143 | 0.07172 | 0 | 0.019847 | 0 | 0 | 0.274816 | 0.087356 | 0.354566 | 0 | 0 | 0 | 0.043844 | 0 | 0 | 0 | 0.581253 | 0.812664 |
| TCGA-24-2288-01A | 0.056292 | 0 | 0.072294 | 0.217855 | 0 | 0.13998 | 0 | 0.084234 | 0.062603 | 0 | 0 | 0.07377 | 0.012435 | 0 | 0.081103 | 0.17403 | 0 | 0 | 0 | 0.008567 | 0 | 0.016836 | 0.04 | 0.220527 | 1.004308 |
| TCGA-24-2289-01A | 0.008977 | 0 | 0.014962 | 0.071738 | 0 | 0.172998 | 0 | 0.03272 | 0.096444 | 0 | 0 | 0.072479 | 0.006926 | 0 | 0.023325 | 0.199496 | 0 | 0.005501 | 0 | 0.216089 | 0.034228 | 0.044117 | 0.28 | 0.092953 | 1.048387 |
| TCGA-24-2290-01A | 0.001715 | 0 | 0 | 0.019098 | 0 | 0.107654 | 0 | 0.031852 | 0.053316 | 0 | 0.004039 | 0.008953 | 0.012464 | 0.074304 | 0.021398 | 0.406492 | 0 | 0.027924 | 0 | 0.209703 | 0 | 0.021089 | 0 | 0.261613 | 0.994334 |
| TCGA-24-2293-01A | 0.019149 | 0 | 0 | 0.111741 | 0 | 0.128373 | 0 | 0 | 0.068444 | 0 | 0 | 0.049099 | 0.041949 | 0.101195 | 0.05186 | 0.387807 | 0 | 0 | 0 | 0.026747 | 0 | 0.013635 | 0 | 0.360811 | 0.940368 |
| TCGA-24-2297-01A | 0.05427 | 0 | 0.10836 | 0.066846 | 0 | 0.218545 | 0 | 0.015275 | 0.115866 | 0 | 0 | 0.116572 | 0.045907 | 0.079594 | 0.04871 | 0.127283 | 0 | 0 | 0 | 0.002771 | 0 | 0 | 0.4 | 0.038103 | 1.070996 |
| TCGA-24-2298-01A | 0.009907 | 0 | 0 | 0.085528 | 0 | 0.132577 | 0 | 0.047059 | 0.048839 | 0 | 0 | 0.067543 | 0 | 0.075329 | 0.01692 | 0.350633 | 0 | 0.051418 | 0 | 0.072124 | 0 | 0.042123 | 0.3 | 0.074515 | 1.069595 |
| TCGA-25-1312-01A | 0.040096 | 0 | 0 | 0.075895 | 0 | 0.118237 | 0 | 0.023509 | 0.035822 | 0 | 0 | 0.089359 | 0.091992 | 0 | 0.027482 | 0.372828 | 0 | 0.079636 | 0 | 0.008116 | 0 | 0.037028 | 0.42 | 0.029459 | 1.083947 |
| TCGA-25-1313-01A | 0 | 0.021673 | 0.027258 | 0.033039 | 0 | 0.123546 | 0 | 0.043791 | 0.063652 | 0 | 0 | 0.116965 | 0.035109 | 0 | 0.021233 | 0.387561 | 0 | 0.022652 | 0.087166 | 0 | 0 | 0.016356 | 0.09 | 0.17311 | 1.015855 |
| TCGA-25-1315-01A | 0 | 0.002609 | 0.044366 | 0 | 0 | 0.274828 | 0 | 0 | 0.038945 | 0 | 0 | 0.079694 | 0.004912 | 0.019292 | 0.029229 | 0.309488 | 0 | 0 | 0.168828 | 0 | 0 | 0.02781 | 0.54 | 0.014916 | 1.074522 |
| TCGA-25-1316-01A | 0.010653 | 0 | 0 | 0.300375 | 0 | 0.100014 | 0 | 0.038777 | 0.060065 | 0 | 0 | 0.115164 | 0.003434 | 0.0004 | 0.07508 | 0.212874 | 0.0003 | 0 | 0.082851 | 0 | 0 | 0 | 0.09 | 0.179464 | 1.037109 |
| TCGA-25-1317-01A | 0.090282 | 0 | 0.020407 | 0.057593 | 0 | 0.137856 | 0 | 0.015509 | 0.082611 | 0 | 0 | 0.075515 | 0.00999 | 0.111353 | 0.104376 | 0.181496 | 0 | 0 | 0 | 0.062576 | 0 | 0.050436 | 0.39 | 0.041185 | 1.076949 |
| TCGA-25-1318-01A | 0 | 0.045269 | 0.005271 | 0.031381 | 0 | 0.122334 | 0 | 0.041655 | 0.058083 | 0 | 0 | 0.079014 | 0.003457 | 0.163571 | 0.001182 | 0.384581 | 0 | 0 | 0 | 0.058925 | 0.005275 | 0 | 0.15 | 0.125719 | 1.064088 |
| TCGA-25-1319-01A | 0.02459 | 0 | 0 | 0.116573 | 0 | 0.178832 | 0 | 0.011997 | 0.061508 | 0 | 0 | 0.085886 | 0.081663 | 0 | 0.087274 | 0.339035 | 0 | 0 | 0.004615 | 0 | 0 | 0.008026 | 0.14 | 0.137838 | 1.042323 |
| TCGA-25-1320-01A | 0.056424 | 0 | 0 | 0.048848 | 0 | 0.150321 | 0 | 0.008317 | 0.032332 | 0 | 0 | 0.039768 | 0.005581 | 0.114356 | 0.040063 | 0.332717 | 0.066835 | 0.04461 | 0 | 0.049762 | 0 | 0.010068 | 0.12 | 0.143274 | 1.042849 |
| TCGA-25-1321-01A | 0 | 0.0009 | 0 | 0.038529 | 0 | 0.177058 | 0 | 0.067411 | 0.12473 | 0 | 0 | 0.111695 | 0.026433 | 0.021315 | 0.084051 | 0.213972 | 0.010756 | 0.009985 | 0.072704 | 0 | 0 | 0.040428 | 0.3 | 0.07372 | 1.05991 |
| TCGA-25-1322-01A | 0.031355 | 0 | 0 | 0.010571 | 0 | 0.173064 | 0 | 0.014336 | 0.076666 | 0 | 0 | 0.110237 | 0.028154 | 0 | 0.022399 | 0.369452 | 0 | 0.048334 | 0 | 0.048964 | 0.010558 | 0.055909 | 0.3 | 0.081387 | 1.058129 |
| TCGA-25-1323-01A | 0 | 0.077488 | 0.001005 | 0 | 0 | 0.344379 | 0 | 0.041701 | 0.122067 | 0 | 0 | 0.099837 | 0.016506 | 0.183445 | 0.017304 | 0.081407 | 0 | 0.004234 | 0.010628 | 0 | 0 | 0 | 0.3 | 0.073116 | 1.077412 |
| TCGA-25-1326-01A | 0.001989 | 0 | 0.01292 | 0.0995 | 0 | 0.136256 | 0 | 0.045956 | 0.076829 | 0 | 0 | 0.05422 | 0.003605 | 0.091219 | 0.144379 | 0.248787 | 0.006234 | 0 | 0.061172 | 0 | 0 | 0.016934 | 0 | 0.307233 | 0.96619 |
| TCGA-25-1328-01A | 0.014606 | 0 | 0 | 0.09561 | 0 | 0.152888 | 0 | 0.01721 | 0.075508 | 0 | 0 | 0.126711 | 0.020286 | 0.105825 | 0.037623 | 0.245381 | 0.048094 | 0 | 0 | 0.029136 | 0 | 0.031121 | 0.15 | 0.124709 | 1.049757 |
| TCGA-25-1329-01A | 0.031335 | 0 | 0.006352 | 0.09004 | 0 | 0.085261 | 0 | 0.036192 | 0.065585 | 0 | 0 | 0.070002 | 0 | 0.156065 | 0.024059 | 0.390236 | 0 | 0 | 0 | 0.019939 | 0 | 0.024933 | 0.33 | 0.05415 | 1.09876 |
| TCGA-25-1623-01A | 0 | 0 | 0 | 0.131135 | 0 | 0.087273 | 0 | 0.108902 | 0.059105 | 0.044905 | 0 | 0.079298 | 0.007669 | 0.051981 | 0.141906 | 0.248498 | 0 | 0 | 0 | 0.01351 | 0 | 0.02582 | 0 | 0.353261 | 0.947485 |
| TCGA-25-1626-01A | 0.005136 | 0 | 0 | 0.159376 | 0 | 0.179562 | 0 | 0.030998 | 0.059157 | 0 | 0 | 0.069096 | 0.052996 | 0 | 0.032891 | 0.280147 | 0.01207 | 0.029709 | 0 | 0.039202 | 0.025133 | 0.024528 | 0.09 | 0.182861 | 1.016388 |
| TCGA-25-1627-01A | 0.085081 | 0 | 0.009546 | 0.04516 | 0 | 0.096252 | 0 | 0.032228 | 0.028342 | 0 | 0 | 0.063696 | 0 | 0.177126 | 0.023947 | 0.300324 | 0.017307 | 0.003558 | 0 | 0.068785 | 0.039266 | 0.009382 | 0.3 | 0.087506 | 1.072193 |
| TCGA-25-1628-01A | 0 | 0.007873 | 0 | 0.275863 | 0 | 0.106373 | 0 | 0.056593 | 0.104507 | 0 | 0 | 0.029212 | 0.011127 | 0.03499 | 0.113097 | 0.168632 | 0.008356 | 0 | 0.071823 | 0 | 0 | 0.011554 | 0 | 0.289534 | 0.976313 |
| TCGA-25-1630-01A | 0.010212 | 0 | 0 | 0.038844 | 0 | 0.030186 | 0 | 0.024322 | 0.043127 | 0 | 0 | 0.039428 | 0 | 0.230376 | 0.021025 | 0.285591 | 0.060449 | 0 | 0 | 0.109811 | 0 | 0.106629 | 0 | 0.493365 | 0.871684 |
| TCGA-25-1631-01A | 0.051628 | 0 | 0 | 0.084292 | 0 | 0.140284 | 0 | 0 | 0.080486 | 0 | 0 | 0.077324 | 0.005913 | 0.100991 | 0.042132 | 0.339188 | 0 | 0 | 0 | 0.036848 | 0 | 0.040914 | 0.3 | 0.067835 | 1.075089 |
| TCGA-25-1632-01A | 0.064605 | 0 | 0 | 0.058855 | 0 | 0.065015 | 0 | 0 | 0.080345 | 0 | 0 | 0.071663 | 0.009206 | 0 | 0 | 0.183203 | 0 | 0.122217 | 0 | 0.300025 | 0.012138 | 0.032727 | 0.99 | -0.0539 | 1.117712 |
| TCGA-25-1633-01A | 0.009405 | 0 | 0 | 0.182398 | 0 | 0.210817 | 0 | 0.071906 | 0.069177 | 0 | 0 | 0.041843 | 0.017362 | 0 | 0.098664 | 0.228326 | 0 | 0.003614 | 0 | 0.040881 | 0 | 0.025609 | 0.07 | 0.19915 | 1.018493 |
| TCGA-25-1634-01A | 0 | 0.004055 | 0.039893 | 0 | 0 | 0.299588 | 0 | 0.029821 | 0.02405 | 0 | 0 | 0.173875 | 0 | 0.219842 | 0 | 0.027044 | 0 | 0 | 0.181831 | 0 | 0 | 0 | 0.57 | 0.011579 | 1.108877 |
| TCGA-25-1635-01A | 0.013765 | 0 | 0 | 0.139587 | 0 | 0.261852 | 0 | 0.008086 | 0.058171 | 0 | 0 | 0.073028 | 0.03915 | 0 | 0.049875 | 0.297489 | 0 | 0.009595 | 0 | 0 | 0 | 0.0494 | 0.07 | 0.201222 | 1.015363 |
| TCGA-25-1870-01A | 0 | 0.140237 | 0.001327 | 0.107057 | 0 | 0.080357 | 0 | 0.034379 | 0.036467 | 0 | 0 | 0.166344 | 0.001178 | 0.129551 | 0.060539 | 0.16541 | 0.038145 | 0 | 0.018047 | 0 | 0.001504 | 0.019457 | 0.3 | 0.072538 | 1.071893 |
| TCGA-25-1877-01A | 0 | 0.060058 | 0 | 0.111829 | 0 | 0 | 0.001355 | 0.017348 | 0 | 0.083612 | 0 | 0.058871 | 0 | 0.185082 | 0.140501 | 0.253878 | 0.017229 | 0 | 0.070236 | 0 | 0 | 0 | 0.09 | 0.187494 | 1.040699 |
| TCGA-25-2042-01A | 0.004051 | 0 | 0 | 0.099092 | 0 | 0.165147 | 0 | 0 | 0.105552 | 0 | 0 | 0.062701 | 0.058415 | 0 | 0.020872 | 0.296911 | 0 | 0.028143 | 0 | 0.071442 | 0.042691 | 0.044982 | 0.39 | 0.041209 | 1.06942 |
| TCGA-25-2391-01A | 0.013437 | 0 | 0 | 0.122902 | 0 | 0.07695 | 0 | 0.035533 | 0.074791 | 0 | 0 | 0.073777 | 0.007773 | 0 | 0.006561 | 0.229488 | 0 | 0.113526 | 0 | 0.089535 | 0.05156 | 0.104165 | 0.33 | 0.063407 | 1.059939 |
| TCGA-25-2392-01A | 0 | 0.039037 | 0 | 0.048335 | 0 | 0.075099 | 0 | 0.029465 | 0.114461 | 0 | 0.014733 | 0.014483 | 0.008097 | 0 | 0.047663 | 0.490039 | 0 | 0.009817 | 0 | 0.061501 | 0 | 0.047271 | 0.23 | 0.105083 | 1.059224 |
| TCGA-25-2393-01A | 0.041499 | 0 | 0 | 0.084946 | 0 | 0.03821 | 0 | 0.053468 | 0.006596 | 0.008696 | 0 | 0.030696 | 0.001065 | 0.188898 | 0.047364 | 0.278383 | 0.007955 | 0 | 0 | 0.193683 | 0 | 0.018541 | 0.05 | 0.214543 | 1.023083 |
| TCGA-25-2396-01A | 0.0009 | 0 | 0 | 0.134648 | 0 | 0.211875 | 0 | 0.059695 | 0.051889 | 0 | 0 | 0.100559 | 0.018297 | 0 | 0.102856 | 0.234543 | 0 | 0 | 0 | 0.037215 | 0 | 0.047538 | 0.05 | 0.210264 | 1.013459 |
| TCGA-25-2398-01A | 0 | 0.034443 | 0.00253 | 0.044461 | 0 | 0.099764 | 0 | 0.042869 | 0.051612 | 0 | 0 | 0 | 0 | 0.482356 | 0.007647 | 0.211178 | 0 | 0 | 0.023138 | 0 | 0 | 0 | 0 | 0.3066 | 1.029628 |
| TCGA-25-2399-01A | 0.114809 | 0 | 0 | 0.102635 | 0 | 0.087784 | 0.015795 | 0.00395 | 0.056587 | 0 | 0 | 0.169397 | 0.020469 | 0.042529 | 0.097171 | 0.261529 | 0 | 0 | 0 | 0.005979 | 0 | 0.021366 | 0.03 | 0.229694 | 1.004985 |
| TCGA-25-2400-01A | 0 | 0.0001 | 0 | 0.085661 | 0 | 0.17278 | 0 | 0.050968 | 0.055829 | 0 | 0 | 0.06131 | 0.005621 | 0 | 0.062069 | 0.421085 | 0 | 0.002528 | 0.051306 | 0 | 0 | 0.030705 | 0.3 | 0.070649 | 1.069473 |
| TCGA-25-2401-01A | 0.005721 | 0 | 0 | 0.081871 | 0 | 0.141694 | 0 | 0.040528 | 0.039941 | 0 | 0 | 0.047111 | 0.062564 | 0 | 0.107095 | 0.398617 | 0.011643 | 0 | 0 | 0.048582 | 0 | 0.014633 | 0 | 0.314267 | 0.960804 |
| TCGA-25-2404-01A | 0.033904 | 0 | 0 | 0.109744 | 0 | 0.175361 | 0 | 0.056008 | 0.011686 | 0 | 0 | 0.112656 | 0 | 0 | 0.156085 | 0.273787 | 0 | 0 | 0 | 0.03056 | 0 | 0.040208 | 0.2 | 0.107995 | 1.069286 |
| TCGA-25-2409-01A | 0 | 0.003728 | 0 | 0.23551 | 0 | 0.018592 | 0 | 0.011213 | 0.131264 | 0 | 0 | 0.167362 | 0.028297 | 0 | 0.088258 | 0.248474 | 0.004779 | 0 | 0 | 0.021093 | 0 | 0.041429 | 0.16 | 0.123402 | 1.065797 |
| TCGA-29-1688-01A | 0 | 0.034382 | 0 | 0.082927 | 0 | 0.128542 | 0 | 0.045601 | 0.093107 | 0 | 0 | 0.096855 | 0.003111 | 0.021925 | 0.058069 | 0.394467 | 0 | 0.00405 | 0 | 0.008086 | 0 | 0.028877 | 0.11 | 0.154442 | 1.034703 |
| TCGA-29-1690-01A | 0.026449 | 0 | 0 | 0.039201 | 0 | 0.17165 | 0 | 0.015074 | 0.055931 | 0 | 0 | 0.01128 | 0.117185 | 0.033375 | 0 | 0.44023 | 0.008613 | 0.062938 | 0 | 0.004915 | 0 | 0.013158 | 0 | 0.376562 | 0.931583 |
| TCGA-29-1691-01A | 0.01949 | 0 | 0 | 0.122556 | 0 | 0.188269 | 0 | 0.030636 | 0.098732 | 0 | 0 | 0.066622 | 0.013606 | 0 | 0.140607 | 0.226592 | 0 | 0 | 0.065107 | 0 | 0 | 0.027784 | 0.18 | 0.113282 | 1.055713 |
| TCGA-29-1693-01A | 0 | 0.081373 | 0.010539 | 0.073444 | 0 | 0.075597 | 0 | 0.054145 | 0.106464 | 0.003661 | 0 | 0.130564 | 0 | 0.155407 | 0.109892 | 0.125317 | 0.007787 | 0 | 0.044309 | 0 | 0 | 0.0215 | 0.14 | 0.135213 | 1.043826 |
| TCGA-29-1694-01A | 0 | 0 | 0 | 0.057863 | 0 | 0.325026 | 0 | 0.023351 | 0.057563 | 0 | 0 | 0.09391 | 0.01255 | 0.047379 | 0.056037 | 0.179749 | 0.040508 | 0.043689 | 0 | 0.041179 | 0 | 0.021195 | 0.33 | 0.062931 | 1.073743 |
| TCGA-29-1695-01A | 0.003108 | 0 | 0 | 0.054116 | 0 | 0.076569 | 0 | 0.024297 | 0.02508 | 0 | 0 | 0.041585 | 0 | 0.264105 | 0.06041 | 0.348338 | 0.005179 | 0 | 0 | 0.084835 | 0 | 0.012378 | 0 | 0.73854 | 0.688565 |
| TCGA-29-1696-01A | 0 | 0 | 0 | 0.205053 | 0 | 0.031891 | 0 | 0.04241 | 0.170293 | 0 | 0 | 0.025014 | 0.031959 | 0.100821 | 0.101623 | 0.103659 | 0.019603 | 0.006868 | 0.144249 | 0 | 0 | 0.016557 | 0.12 | 0.143076 | 1.032657 |
| TCGA-29-1697-01A | 0 | 0.00577 | 0.055264 | 0.048143 | 0 | 0.118848 | 0 | 0.040385 | 0.040797 | 0 | 0 | 0.181459 | 0.003508 | 0.1347 | 0.106673 | 0.1169 | 0 | 0 | 0.147554 | 0 | 0 | 0 | 0.53 | 0.019458 | 1.095746 |
| TCGA-29-1701-01A | 0.005376 | 0 | 0.011773 | 0.17577 | 0 | 0.13683 | 0.044802 | 0.067285 | 0.008714 | 0.026573 | 0 | 0.027716 | 0.026537 | 0 | 0.112787 | 0.286228 | 0 | 0.016493 | 0 | 0.010676 | 0.01831 | 0.024132 | 0 | 0.266062 | 0.984567 |
| TCGA-29-1703-01A | 0 | 0 | 0 | 0.10866 | 0 | 0.066598 | 0 | 0.058074 | 0.079154 | 0 | 0 | 0.081227 | 0.017281 | 0 | 0.083825 | 0.29888 | 0.040145 | 0 | 0 | 0.166156 | 0 | 0 | 0.36 | 0.048625 | 1.072933 |
| TCGA-29-1705-01A | 0.024519 | 0 | 0 | 0.048128 | 0 | 0.178469 | 0 | 0.013203 | 0.036545 | 0 | 0 | 0.052643 | 0 | 0.227662 | 0.042997 | 0.346165 | 0 | 0 | 0 | 0.011006 | 0 | 0.018662 | 0.04 | 0.216395 | 1.030971 |
| TCGA-29-1710-01A | 0 | 0.05675 | 0 | 0.103798 | 0 | 0.090241 | 0 | 0.047369 | 0.088909 | 0 | 0 | 0.174541 | 0.015409 | 0.06323 | 0.104819 | 0.186297 | 0.059696 | 0 | 0 | 0.008942 | 0 | 0 | 0 | 0.299915 | 0.970834 |
| TCGA-29-1711-01A | 0.047739 | 0 | 0 | 0.118406 | 0 | 0.227623 | 0 | 0.068775 | 0.050964 | 0.001813 | 0 | 0.071987 | 0 | 0.027361 | 0.137152 | 0.226642 | 0 | 0 | 0 | 0.00824 | 0 | 0.013296 | 0.04 | 0.221117 | 1.012206 |
| TCGA-29-1761-01A | 0.0005 | 0 | 0 | 0.127737 | 0 | 0.013272 | 0 | 0.05442 | 0.032757 | 0.054481 | 0 | 0.014952 | 0 | 0.195093 | 0.117288 | 0.366712 | 0 | 0 | 0 | 0.016306 | 0 | 0.006523 | 0 | 0.590627 | 0.806007 |
| TCGA-29-1762-01A | 0 | 0.004226 | 0.036442 | 0 | 0 | 0.251259 | 0 | 0 | 0.028333 | 0 | 0 | 0.069378 | 0.190606 | 0 | 0 | 0.278617 | 0 | 0 | 0 | 0 | 0.105602 | 0.035537 | 0.33 | 0.053639 | 1.071733 |
| TCGA-29-1763-01A | 0.057292 | 0 | 0 | 0.047428 | 0 | 0.102875 | 0 | 0 | 0.064111 | 0 | 0 | 0.080696 | 0.007935 | 0 | 0.044885 | 0.458445 | 0.028652 | 0 | 0 | 0.047722 | 0 | 0.059961 | 0.3 | 0.082269 | 1.067257 |
| TCGA-29-1766-01A | 0 | 0.074281 | 0 | 0.219983 | 0 | 0.046604 | 0 | 0.048209 | 0.11642 | 0 | 0 | 0.104058 | 0.0054 | 0.116935 | 0.06652 | 0.094146 | 0.0001 | 0.060102 | 0 | 0.03196 | 0 | 0.015296 | 0.07 | 0.199051 | 1.018054 |
| TCGA-29-1768-01A | 0.001585 | 0 | 0 | 0.136209 | 0 | 0.146757 | 0 | 0.041863 | 0.095743 | 0 | 0 | 0.149759 | 0.00907 | 0.02548 | 0.101743 | 0.234956 | 0.012193 | 0 | 0.024961 | 0 | 0 | 0.019681 | 0 | 0.354372 | 0.944553 |
| TCGA-29-1769-01A | 0.006198 | 0 | 0 | 0.109307 | 0 | 0.252464 | 0 | 0.050142 | 0.003649 | 0 | 0 | 0.086694 | 0.005504 | 0 | 0.052226 | 0.239482 | 0 | 0.053122 | 0 | 0.049329 | 0 | 0.091883 | 0.23 | 0.106258 | 1.053846 |
| TCGA-29-1770-01A | 0 | 0.030512 | 0.00108 | 0.058385 | 0 | 0.163891 | 0 | 0.04451 | 0.034172 | 0 | 0 | 0.067779 | 0.004259 | 0.154323 | 0.02246 | 0.316562 | 0 | 0.022232 | 0 | 0.065187 | 0 | 0.014647 | 0.39 | 0.039991 | 1.094177 |
| TCGA-29-1774-01A | 0.126161 | 0 | 0.016383 | 0.076047 | 0 | 0.14521 | 0 | 0 | 0.106536 | 0 | 0 | 0.087185 | 0.047056 | 0.253269 | 0.00554 | 0.136614 | 0 | 0 | 0 | 0 | 0 | 0 | 0.42 | 0.027982 | 1.101273 |
| TCGA-29-1776-01A | 0.030766 | 0 | 0 | 0.125264 | 0 | 0.257359 | 0 | 0.039168 | 0.061877 | 0 | 0 | 0.042075 | 0.006694 | 0 | 0.024878 | 0.279131 | 0 | 0.057079 | 0 | 0.018363 | 0 | 0.057345 | 0.18 | 0.115387 | 1.048679 |
| TCGA-29-1777-01A | 0.002002 | 0 | 0.026934 | 0.056187 | 0 | 0.150011 | 0 | 0.114482 | 0 | 0 | 0 | 0.096671 | 0 | 0.030296 | 0.119035 | 0.197347 | 0.028236 | 0.018109 | 0 | 0.117765 | 0.009528 | 0.033396 | 0.05 | 0.210728 | 1.004801 |
| TCGA-29-1778-01A | 0.011114 | 0 | 0 | 0.0839 | 0 | 0.121779 | 0 | 0.053023 | 0.093323 | 0.0007 | 0 | 0.11642 | 0 | 0.042922 | 0.085045 | 0.23221 | 0.01881 | 0 | 0.104176 | 0 | 0 | 0.036556 | 0.09 | 0.179798 | 1.015877 |
| TCGA-29-1781-01A | 0.014095 | 0 | 0.090017 | 0.187436 | 0 | 0 | 0.009466 | 0.044303 | 0.044895 | 0.012286 | 0 | 0.049942 | 0 | 0.121011 | 0.11824 | 0.290212 | 0 | 0 | 0 | 0.004551 | 0 | 0.013548 | 0 | 0.47195 | 0.880297 |
| TCGA-29-1783-01A | 0 | 0.001673 | 0.005793 | 0.093538 | 0 | 0.143755 | 0 | 0.050649 | 0.043798 | 0 | 0 | 0.043823 | 0.008848 | 0.055151 | 0.088714 | 0.374578 | 0.023529 | 0 | 0 | 0.026725 | 0 | 0.039427 | 0.09 | 0.186154 | 1.023679 |
| TCGA-29-1784-01A | 0 | 0.055247 | 0 | 0.158506 | 0 | 0.057796 | 0 | 0.063471 | 0.078007 | 0 | 0 | 0.041325 | 0.02231 | 0.058249 | 0.129913 | 0.273555 | 0.020859 | 0 | 0.040762 | 0 | 0 | 0 | 0 | 0.387884 | 0.923478 |
| TCGA-29-1785-01A | 0.005966 | 0 | 0 | 0.189006 | 0 | 0.115584 | 0.0002 | 0.045078 | 0.087013 | 0 | 0 | 0.09725 | 0.009458 | 0 | 0.172425 | 0.26127 | 0.001544 | 0 | 0 | 0.002836 | 0 | 0.012347 | 0 | 0.425277 | 0.911028 |
| TCGA-29-2414-01A | 0 | 0.036457 | 0.014924 | 0.100683 | 0 | 0.172726 | 0 | 0.05136 | 0.040929 | 0 | 0 | 0.122453 | 0.014912 | 0.247531 | 0.004508 | 0.032058 | 0.004328 | 0 | 0.157131 | 0 | 0 | 0 | 0.4 | 0.036281 | 1.093688 |
| TCGA-29-2425-01A | 0.020692 | 0 | 0 | 0.127849 | 0 | 0.020155 | 0 | 0.063689 | 0.055671 | 0 | 0 | 0.078004 | 0.001968 | 0.251509 | 0.069309 | 0.270915 | 0.019165 | 0 | 0 | 0.016605 | 0 | 0.004469 | 0.19 | 0.109602 | 1.084547 |
| TCGA-29-2427-01A | 0 | 0.078773 | 0 | 0.155471 | 0 | 0.086873 | 0 | 0.057465 | 0.05951 | 0 | 0 | 0.085582 | 0.004318 | 0.038021 | 0.097037 | 0.302495 | 0.006606 | 0 | 0.02785 | 0 | 0 | 0 | 0 | 0.330955 | 0.950931 |
| TCGA-29-2428-01A | 0 | 0.002589 | 0 | 0.147976 | 0 | 0.060352 | 0.034895 | 0.060251 | 0.039428 | 0.045498 | 0 | 0.012887 | 0 | 0.135675 | 0.084389 | 0.343675 | 0.001926 | 0 | 0 | 0.030458 | 0 | 0 | 0 | 0.324359 | 0.962137 |
| TCGA-29-A5NZ-01A | 0.051632 | 0 | 0 | 0.034232 | 0 | 0.208771 | 0 | 0.026682 | 0.030425 | 0 | 0.032484 | 0 | 0 | 0.28945 | 0.142087 | 0.078565 | 0.028081 | 0 | 0 | 0.07759 | 0 | 0 | 0.11 | 0.162312 | 1.059197 |
| TCGA-30-1714-01A | 0.078053 | 0 | 0 | 0.022702 | 0 | 0.112456 | 0 | 0.054176 | 0 | 0 | 0 | 0.119138 | 0.014143 | 0 | 0.011653 | 0.359113 | 0.007789 | 0.01098 | 0 | 0.176695 | 0 | 0.033103 | 0.43 | 0.027478 | 1.082463 |
| TCGA-30-1718-01A | 0 | 0.004522 | 0.001879 | 0.104545 | 0 | 0.213526 | 0 | 0.066065 | 0.054812 | 0 | 0 | 0.08406 | 0 | 0 | 0.060571 | 0.282428 | 0 | 0.032708 | 0 | 0.028184 | 0 | 0.0667 | 0.18 | 0.11224 | 1.050981 |
| TCGA-30-1853-01A | 0.005332 | 0 | 0.017464 | 0 | 0 | 0.3908 | 0 | 0.020817 | 0.075344 | 0 | 0 | 0.113883 | 0 | 0.171205 | 0.0005 | 0.031924 | 0 | 0 | 0.170222 | 0 | 0 | 0.002532 | 0.71 | -0.0087 | 1.112476 |
| TCGA-30-1857-01A | 0 | 0.083771 | 0 | 0.090915 | 0 | 0.130979 | 0 | 0.030462 | 0.080443 | 0 | 0.02361 | 0 | 0.023242 | 0.1691 | 0.080662 | 0.256623 | 0.005267 | 0 | 0 | 0.024927 | 0 | 0 | 0 | 0.306441 | 0.966354 |
| TCGA-30-1860-01A | 0 | 0 | 0.021398 | 0.148647 | 0 | 0.049645 | 0 | 0.061184 | 0.126776 | 0 | 0 | 0.13982 | 0.012176 | 0.008345 | 0.250429 | 0.134878 | 0 | 0 | 0 | 0.040605 | 0 | 0.006096 | 0 | 0.458612 | 0.901033 |
| TCGA-30-1861-01A | 0.013042 | 0 | 0 | 0.038601 | 0 | 0.217912 | 0 | 0 | 0.040917 | 0 | 0.010833 | 0.010619 | 0 | 0.139367 | 0.084384 | 0.39634 | 0 | 0 | 0 | 0.04175 | 0 | 0.006235 | 0.57 | 0.010517 | 1.123621 |
| TCGA-30-1862-01A | 0.007179 | 0 | 0.031941 | 0.14665 | 0 | 0.247636 | 0 | 0.026266 | 0.061627 | 0 | 0 | 0.083569 | 0.008478 | 0 | 0.0978 | 0.263224 | 0 | 0 | 0 | 0.005646 | 0 | 0.019984 | 0.19 | 0.109917 | 1.060385 |
| TCGA-30-1866-01A | 0 | 0.026548 | 0 | 0.328825 | 0 | 0 | 0.005378 | 0.110731 | 0.015913 | 0 | 0 | 0.119595 | 0.035779 | 0 | 0.074735 | 0.188163 | 0.013653 | 0 | 0.080681 | 0 | 0 | 0 | 0.33 | 0.0602 | 1.08961 |
| TCGA-30-1891-01A | 0.001477 | 0 | 0.002235 | 0.178275 | 0 | 0.090119 | 0 | 0.027433 | 0.061361 | 0 | 0 | 0.110547 | 0 | 0.139292 | 0.076722 | 0.212542 | 0 | 0 | 0 | 0.038757 | 0 | 0.061241 | 0.05 | 0.209652 | 1.01879 |
| TCGA-30-1892-01A | 0.019958 | 0 | 0 | 0.075997 | 0 | 0.108121 | 0 | 0.024144 | 0.049725 | 0 | 0 | 0.083219 | 0 | 0.231616 | 0.036077 | 0.304177 | 0 | 0 | 0 | 0.025308 | 0.014159 | 0.027499 | 0.07 | 0.195197 | 1.038119 |
| TCGA-31-1944-01A | 0.041766 | 0 | 0 | 0.061459 | 0 | 0.226359 | 0.022145 | 0.031918 | 0.034935 | 0 | 0 | 0.078707 | 0.046212 | 0 | 0.062276 | 0.26312 | 0.024163 | 0 | 0 | 0.061678 | 0.014395 | 0.030867 | 0.14 | 0.137802 | 1.029491 |
| TCGA-31-1946-01A | 0.039726 | 0 | 0 | 0.050345 | 0 | 0.088977 | 0 | 0.03531 | 0.047262 | 0 | 0 | 0.035325 | 0 | 0.264463 | 0.048281 | 0.368314 | 0 | 0 | 0 | 0.021997 | 0 | 0 | 0 | 0.383972 | 0.943312 |
| TCGA-31-1950-01A | 0.043292 | 0 | 0 | 0.070522 | 0 | 0.16244 | 0.011654 | 0.057268 | 0.058667 | 0 | 0 | 0.049353 | 0.013731 | 0 | 0.17757 | 0.3162 | 0 | 0.001693 | 0 | 0.012316 | 0 | 0.025294 | 0.11 | 0.150499 | 1.049106 |
| TCGA-31-1951-01A | 0 | 0 | 0.018865 | 0.081198 | 0 | 0.098825 | 0 | 0.081216 | 0.113973 | 0 | 0 | 0.084474 | 0 | 0.057858 | 0.191756 | 0.16525 | 0.007027 | 0 | 0 | 0.075592 | 0 | 0.023966 | 0 | 0.283381 | 0.985102 |
| TCGA-31-1953-01A | 0.001938 | 0 | 0 | 0.046637 | 0 | 0.145493 | 0 | 0.021205 | 0.081755 | 0 | 0.017762 | 0.083818 | 0.027835 | 0.0895 | 0.09145 | 0.235687 | 0.127386 | 0 | 0 | 0.021255 | 0 | 0.00828 | 0 | 0.499833 | 0.865416 |
| TCGA-31-1956-01A | 0.031789 | 0 | 0 | 0.146024 | 0 | 0.072155 | 0.039027 | 0.055875 | 0.016927 | 0 | 0 | 0.035839 | 0 | 0.1554 | 0.059222 | 0.311863 | 0.042701 | 0 | 0 | 0.033178 | 0 | 0 | 0 | 0.462 | 0.885971 |
| TCGA-31-1959-01A | 0.002134 | 0 | 0 | 0.089717 | 0 | 0.222999 | 0 | 0.049682 | 0.038747 | 0 | 0 | 0.049555 | 0.004226 | 0.066771 | 0.033824 | 0.332346 | 0.014421 | 0.057673 | 0 | 0.020619 | 0 | 0.017286 | 0 | 0.3187 | 0.958633 |
| TCGA-36-1568-01A | 0 | 0.027535 | 0 | 0.082587 | 0 | 0.153409 | 0 | 0 | 0.135893 | 0 | 0 | 0.129802 | 0.018237 | 0.143825 | 0.038469 | 0.252666 | 0 | 0 | 0 | 0.017577 | 0 | 0 | 0 | 0.306036 | 0.967948 |
| TCGA-36-1569-01A | 0.018081 | 0 | 0 | 0.160744 | 0 | 0.207753 | 0 | 0.054928 | 0.055555 | 0 | 0 | 0.055778 | 0.008457 | 0.072883 | 0.110251 | 0.161032 | 0.056856 | 0 | 0.01587 | 0 | 0 | 0.021812 | 0 | 0.266006 | 0.986181 |
| TCGA-36-1570-01A | 0 | 0.03519 | 0.003436 | 0.066147 | 0 | 0.126167 | 0 | 0.045671 | 0.076065 | 0 | 0 | 0.084045 | 0.061468 | 0.10946 | 0.081423 | 0.143502 | 0.0009 | 0.0008 | 0.136171 | 0 | 0.029645 | 0 | 0.12 | 0.145774 | 1.023949 |
| TCGA-36-1571-01A | 0 | 0.015597 | 0 | 0 | 0 | 0.509375 | 0 | 0.03855 | 0.19534 | 0 | 0 | 0.043197 | 0.039238 | 0.039896 | 0.040002 | 0.010366 | 0 | 0.044382 | 0.024057 | 0 | 0 | 0 | 0.3 | 0.082457 | 1.093793 |
| TCGA-36-1574-01A | 0.037566 | 0 | 0 | 0.035527 | 0 | 0.211349 | 0 | 0.05147 | 0.011668 | 0 | 0.007694 | 0.023913 | 0.008332 | 0.206723 | 0.139327 | 0.152746 | 0 | 0.045433 | 0 | 0.068252 | 0 | 0 | 0 | 0.328834 | 0.963499 |
| TCGA-36-1576-01A | 0 | 0 | 0 | 0.162104 | 0 | 0.182102 | 0 | 0.044073 | 0.037023 | 0 | 0 | 0 | 0.023842 | 0.063538 | 0.119307 | 0.275135 | 0.032971 | 0 | 0.059905 | 0 | 0 | 0 | 0 | 0.32179 | 0.958016 |
| TCGA-36-1577-01A | 0.059863 | 0 | 0 | 0.056672 | 0 | 0.072497 | 0 | 0.016402 | 0.072782 | 0 | 0 | 0.028856 | 0.016091 | 0.245135 | 0 | 0.333559 | 0 | 0.014387 | 0 | 0.038296 | 0 | 0.045461 | 0.38 | 0.044251 | 1.121989 |
| TCGA-36-1580-01A | 0.018736 | 0 | 0 | 0.100758 | 0 | 0.195524 | 0 | 0.047893 | 0.041666 | 0 | 0 | 0.043593 | 0.016954 | 0.140198 | 0.080518 | 0.270878 | 0.022157 | 0 | 0 | 0.013687 | 0 | 0.007438 | 0 | 0.28395 | 0.979104 |
| TCGA-36-1581-01A | 0.019222 | 0 | 0.033853 | 0.185695 | 0 | 0.073163 | 0 | 0.10554 | 0.10489 | 0 | 0 | 0.083176 | 0 | 0.109271 | 0.08447 | 0.144585 | 0 | 0.003649 | 0.011669 | 0 | 0 | 0.040817 | 0.05 | 0.215181 | 1.008605 |
| TCGA-57-1582-01A | 0.007962 | 0 | 0 | 0.130363 | 0 | 0.189905 | 0 | 0.032788 | 0.082902 | 0 | 0 | 0.0226 | 0.0348 | 0.091861 | 0.099515 | 0.247389 | 0 | 0 | 0.050685 | 0 | 0.009229 | 0 | 0.18 | 0.111344 | 1.05367 |
| TCGA-57-1583-01A | 0 | 0.039645 | 0.0002 | 0.033048 | 0 | 0.057776 | 0 | 0.004275 | 0.081094 | 0 | 0 | 0.056505 | 0 | 0.478135 | 0.011371 | 0.150592 | 0 | 0 | 0 | 0.078789 | 0 | 0.0086 | 0.04 | 0.219283 | 1.079579 |
| TCGA-57-1584-01A | 0 | 0.020437 | 0 | 0.078977 | 0 | 0.164407 | 0 | 0.00496 | 0.111476 | 0 | 0 | 0.132379 | 0.017355 | 0 | 0.023971 | 0.360533 | 0 | 0.0006 | 0.084918 | 0 | 0 | 0 | 0.46 | 0.026125 | 1.085129 |
| TCGA-57-1585-01A | 0 | 0.03578 | 0 | 0.088318 | 0 | 0.198824 | 0 | 0.050628 | 0.082675 | 0 | 0 | 0.009986 | 0 | 0.239614 | 0.037418 | 0.238994 | 0 | 0 | 0 | 0.017763 | 0 | 0 | 0 | 0.270608 | 0.994326 |
| TCGA-57-1586-01A | 0.042415 | 0 | 0.011052 | 0.106843 | 0 | 0.190449 | 0 | 0.051096 | 0.130944 | 0 | 0 | 0.011722 | 0.017678 | 0.146736 | 0.082826 | 0.126525 | 0 | 0 | 0.081714 | 0 | 0 | 0 | 0.09 | 0.173539 | 1.021424 |
| TCGA-57-1993-01A | 0.030962 | 0 | 0 | 0.018515 | 0 | 0.083956 | 0 | 0.00954 | 0.091208 | 0 | 0 | 0.031535 | 0 | 0.290291 | 0.035647 | 0.37253 | 0 | 0 | 0.035217 | 0 | 0 | 0.0006 | 0.09 | 0.187036 | 1.064252 |
| TCGA-57-1994-01A | 0.030193 | 0 | 0 | 0.188171 | 0 | 0.179764 | 0 | 0.061696 | 0.08846 | 0 | 0 | 0.023944 | 0 | 0.142727 | 0.086485 | 0.197599 | 0 | 0 | 0 | 0.001 | 0 | 0 | 0 | 0.296794 | 0.975385 |
| TCGA-59-2348-01A | 0.042036 | 0 | 0.131125 | 0.066057 | 0 | 0.140911 | 0 | 0.095393 | 0.045483 | 0 | 0 | 0.056994 | 0.006073 | 0.126947 | 0.087699 | 0.152078 | 0 | 0 | 0 | 0.026948 | 0 | 0.022257 | 0.3 | 0.084691 | 1.049411 |
| TCGA-59-2350-01A | 0 | 0.084392 | 0 | 0.091201 | 0 | 0.097972 | 0 | 0.044605 | 0.099465 | 0 | 0 | 0.115665 | 0.010056 | 0.199155 | 0.062222 | 0.158426 | 0 | 0.0003 | 0.019892 | 0 | 0 | 0.016654 | 0.14 | 0.131326 | 1.048973 |
| TCGA-59-2351-01A | 0 | 0.042971 | 0 | 0.082147 | 0 | 0.123665 | 0 | 0.027725 | 0.080323 | 0 | 0 | 0.066061 | 0.021876 | 0.179841 | 0.1145 | 0.227374 | 0.009024 | 0 | 0.024493 | 0 | 0 | 0 | 0 | 0.427008 | 0.905465 |
| TCGA-59-2352-01A | 0.113521 | 0 | 0.088488 | 0.060249 | 0 | 0.04825 | 0 | 0.066616 | 0.043714 | 0 | 0 | 0.073941 | 0 | 0.182764 | 0.173929 | 0.141398 | 0.003886 | 0 | 0 | 0.003244 | 0 | 0 | 0 | 0.314066 | 0.967193 |
| TCGA-59-2354-01A | 0.048548 | 0 | 0 | 0.089761 | 0 | 0.131109 | 0 | 0.032848 | 0.152489 | 0 | 0 | 0.082397 | 0.006222 | 0.104575 | 0.101214 | 0.225547 | 0 | 0 | 0 | 0.025289 | 0 | 0 | 0 | 0.372604 | 0.931648 |
| TCGA-59-2355-01A | 0.019046 | 0 | 0 | 0.118007 | 0 | 0.225001 | 0 | 0.061343 | 0.06742 | 0 | 0 | 0.103972 | 0.015642 | 0 | 0.138769 | 0.225283 | 0 | 0 | 0 | 0.025518 | 0 | 0 | 0.09 | 0.173701 | 1.037659 |
| TCGA-59-2363-01A | 0 | 0.0002 | 0 | 0.093925 | 0 | 0.075751 | 0 | 0.11984 | 0.079982 | 0 | 0 | 0.12306 | 0.024387 | 0.051597 | 0.060923 | 0.172009 | 0 | 0.023839 | 0 | 0.050567 | 0.031973 | 0.091968 | 0.61 | 0.004137 | 1.09308 |
| TCGA-59-A5PD-01A | 0.039012 | 0 | 0 | 0.025122 | 0 | 0.132714 | 0 | 0.040252 | 0.026784 | 0 | 0 | 0.034961 | 0 | 0.312294 | 0.064672 | 0.227843 | 0 | 0 | 0 | 0.096345 | 0 | 0 | 0 | 0.376834 | 0.945919 |
| TCGA-5X-AA5U-01A | 0.001584 | 0.00901 | 0 | 0 | 0 | 0.284442 | 0 | 0.040333 | 0.074506 | 0 | 0.0459 | 0 | 0 | 0.520769 | 0.004912 | 0 | 0.011748 | 0 | 0 | 0.006796 | 0 | 0 | 0.19 | 0.110389 | 1.143937 |
| TCGA-61-1721-01A | 0.002994 | 0 | 0.010278 | 0.071103 | 0 | 0.174331 | 0 | 0.005561 | 0.04655 | 0 | 0 | 0.075933 | 0.029365 | 0 | 0 | 0.422621 | 0.008643 | 0 | 0.152621 | 0 | 0 | 0 | 0 | 0.261184 | 0.98152 |
| TCGA-61-1724-01A | 0.040244 | 0 | 0.039658 | 0.069202 | 0 | 0.209452 | 0 | 0.01775 | 0.073992 | 0 | 0 | 0.053931 | 0.010275 | 0.144338 | 0.126371 | 0.200503 | 0 | 0 | 0 | 0 | 0 | 0.014283 | 0.09 | 0.190137 | 1.023665 |
| TCGA-61-1725-01A | 0 | 0.002749 | 0.012834 | 0.099725 | 0 | 0.121803 | 0 | 0.034182 | 0.058661 | 0 | 0 | 0.126956 | 0.001345 | 0 | 0.104221 | 0.292077 | 0.0001 | 0 | 0.112508 | 0 | 0 | 0.032799 | 0.01 | 0.246691 | 0.989505 |
| TCGA-61-1728-01A | 0.028676 | 0 | 0 | 0.0293 | 0 | 0.073168 | 0 | 0.028256 | 0.04395 | 0 | 0 | 0.058002 | 0 | 0.269993 | 0.037316 | 0.337323 | 0.004062 | 0 | 0 | 0.07493 | 0 | 0.015026 | 0.09 | 0.179349 | 1.061586 |
| TCGA-61-1733-01A | 0 | 0.001 | 0 | 0.161817 | 0 | 0.12545 | 0 | 0.117544 | 0.011004 | 0 | 0 | 0.078368 | 0.007725 | 0 | 0.06183 | 0.239358 | 0 | 0.043892 | 0 | 0.126924 | 0 | 0.025108 | 0.42 | 0.02905 | 1.084072 |
| TCGA-61-1736-01B | 0.012997 | 0 | 0 | 0.102462 | 0 | 0.120401 | 0 | 0.001796 | 0.099269 | 0 | 0 | 0.133656 | 0.041585 | 0 | 0 | 0.356368 | 0 | 0.04047 | 0 | 0.062032 | 0.008018 | 0.020945 | 0.36 | 0.04789 | 1.078526 |
| TCGA-61-1737-01A | 0 | 0.054199 | 0 | 0.056638 | 0 | 0.120115 | 0 | 0.034966 | 0.036742 | 0 | 0 | 0.058842 | 0.001543 | 0.115165 | 0.07085 | 0.368056 | 0.008487 | 0 | 0 | 0.074397 | 0 | 0 | 0.07 | 0.198372 | 1.020096 |
| TCGA-61-1738-01A | 0.019915 | 0 | 0 | 0.075594 | 0 | 0.044241 | 0 | 0.084523 | 0 | 0 | 0 | 0.069223 | 0.020376 | 0 | 0.090669 | 0.371677 | 0 | 0.136394 | 0 | 0.021008 | 0 | 0.066382 | 0.25 | 0.098508 | 1.068873 |
| TCGA-61-1741-01A | 0.03943 | 0 | 0.034752 | 0.056689 | 0 | 0.188426 | 0 | 0.067158 | 0.055261 | 0 | 0 | 0.014439 | 0.001066 | 0.134248 | 0.179892 | 0.218375 | 0 | 0 | 0 | 0.010265 | 0 | 0 | 0.04 | 0.21822 | 1.021013 |
| TCGA-61-1900-01A | 0.110623 | 0 | 0.024754 | 0.109392 | 0 | 0.187313 | 0 | 0.096334 | 0.009671 | 0 | 0 | 0.087176 | 0.018433 | 0.001073 | 0.084651 | 0.270579 | 0 | 0 | 0 | 0 | 0 | 0 | 0.57 | 0.011832 | 1.091651 |
| TCGA-61-1907-01A | 0.055038 | 0 | 0.003247 | 0.111372 | 0 | 0.08675 | 0 | 0.048853 | 0.055763 | 0 | 0 | 0.059889 | 0.005294 | 0.032419 | 0.146889 | 0.349366 | 0 | 0 | 0 | 0.038619 | 0 | 0.0065 | 0 | 0.36776 | 0.93673 |
| TCGA-61-1910-01A | 0.025445 | 0 | 0 | 0.008692 | 0 | 0.100862 | 0 | 0.027786 | 0.053397 | 0 | 0 | 0.021668 | 0 | 0.098761 | 0.057343 | 0.440927 | 0 | 0.009588 | 0 | 0.101106 | 0 | 0.054425 | 0.12 | 0.148697 | 1.054468 |
| TCGA-61-1911-01A | 0.066297 | 0 | 0.077882 | 0.243511 | 0 | 0.053867 | 0 | 0.053705 | 0.09642 | 0 | 0 | 0.09083 | 0 | 0 | 0.147878 | 0.150036 | 0 | 0 | 0 | 0 | 0 | 0.019573 | 0 | 0.369954 | 0.93719 |
| TCGA-61-1914-01A | 0.034277 | 0 | 0.028334 | 0.039654 | 0 | 0.077175 | 0 | 0.075377 | 0 | 0.040518 | 0 | 0.06367 | 0 | 0.041326 | 0.200209 | 0.238103 | 0 | 0 | 0 | 0.08196 | 0.036921 | 0.042477 | 0 | 0.270705 | 0.992501 |
| TCGA-61-1918-01A | 0.0004 | 0.002443 | 0 | 0.092277 | 0 | 0.112189 | 0 | 0.019711 | 0.189052 | 0 | 0.003264 | 0.032791 | 0.032056 | 0.141129 | 0.072163 | 0.206411 | 0.061338 | 0 | 0 | 0.016834 | 0 | 0.017977 | 0.09 | 0.187167 | 1.019484 |
| TCGA-61-1919-01A | 0.0009 | 0 | 0 | 0.036933 | 0 | 0.146152 | 0.005283 | 0.030819 | 0.013901 | 0 | 0 | 0.026587 | 0 | 0.236293 | 0.138217 | 0.288169 | 0.038611 | 0 | 0 | 0.032194 | 0 | 0.005933 | 0 | 0.530882 | 0.847481 |
| TCGA-61-1995-01A | 0 | 0.025209 | 0 | 0.084279 | 0 | 0.276669 | 0 | 0.026333 | 0.094746 | 0 | 0 | 0.051269 | 0.016237 | 0 | 0.071519 | 0.323902 | 0.003911 | 0 | 0 | 0 | 0 | 0.025926 | 0.08 | 0.192349 | 1.017447 |
| TCGA-61-1998-01A | 0.01041 | 0 | 0 | 0.011144 | 0 | 0.06402 | 0 | 0.024389 | 0.045187 | 0 | 0.01599 | 0 | 0 | 0.3597 | 0.014554 | 0.39408 | 0 | 0 | 0 | 0.038888 | 0 | 0.021637 | 0 | 0.434853 | 0.934978 |
| TCGA-61-2000-01A | 0.010447 | 0 | 0 | 0.037695 | 0 | 0.165506 | 0.021773 | 0.043948 | 0 | 0 | 0 | 0.172317 | 0.006027 | 0 | 0.029266 | 0.294102 | 0.023035 | 0.023946 | 0 | 0.102742 | 0.045219 | 0.023979 | 0.09 | 0.170918 | 1.023661 |
| TCGA-61-2002-01A | 0.01389 | 0 | 0 | 0.202193 | 0 | 0.109768 | 0 | 0.078937 | 0.146976 | 0 | 0 | 0.043201 | 0.030581 | 0.021971 | 0.129396 | 0.161925 | 0.024347 | 0 | 0 | 0.015987 | 0 | 0.020827 | 0 | 0.290634 | 0.976067 |
| TCGA-61-2003-01A | 0 | 0 | 0 | 0.098534 | 0 | 0.153212 | 0 | 0.022318 | 0.062654 | 0 | 0.019036 | 0 | 0.033291 | 0.267793 | 0.042436 | 0.260838 | 0.013713 | 0 | 0.026175 | 0 | 0 | 0 | 0 | 0.549277 | 0.834391 |
| TCGA-61-2008-01A | 0 | 0 | 0 | 0.13293 | 0 | 0.202832 | 0 | 0.050017 | 0.055287 | 0 | 0 | 0.074545 | 0.00972 | 0 | 0.084268 | 0.348953 | 0 | 0 | 0 | 0.021324 | 0.004981 | 0.015144 | 0.16 | 0.12322 | 1.052362 |
| TCGA-61-2009-01A | 0.008591 | 0 | 0 | 0.144753 | 0 | 0.109013 | 0 | 0.05391 | 0.025151 | 0 | 0.007866 | 0 | 0.009019 | 0.046562 | 0.149012 | 0.289375 | 0.052801 | 0 | 0.103947 | 0 | 0 | 0 | 0 | 0.299602 | 0.969022 |
| TCGA-61-2012-01A | 0.046717 | 0 | 0.102601 | 0.125701 | 0 | 0.060797 | 0 | 0.045447 | 0.048735 | 0 | 0 | 0.052573 | 0.003556 | 0.164237 | 0.08704 | 0.115034 | 0 | 0 | 0 | 0.120012 | 0 | 0.027552 | 0 | 0.384201 | 0.923264 |
| TCGA-61-2088-01A | 0 | 0 | 0.04885 | 0 | 0 | 0.358304 | 0 | 0.082303 | 0.055247 | 0 | 0 | 0.011935 | 0.024048 | 0.194196 | 0 | 0 | 0.006689 | 0 | 0.218427 | 0 | 0 | 0 | 0.3 | 0.074943 | 1.069081 |
| TCGA-61-2092-01A | 0.201397 | 0 | 0.06989 | 0.014357 | 0 | 0.069018 | 0 | 0 | 0.061026 | 0 | 0 | 0.048626 | 0 | 0.185903 | 0.005026 | 0.271072 | 0 | 0 | 0 | 0.052656 | 0 | 0.02103 | 0.74 | -0.0156 | 1.112945 |
| TCGA-61-2097-01A | 0.043181 | 0 | 0 | 0 | 0 | 0.120964 | 0 | 0.024678 | 0.015978 | 0 | 0 | 0.04274 | 0.01209 | 0 | 0.009794 | 0.33153 | 0 | 0.037856 | 0 | 0.243627 | 0.084669 | 0.032892 | 0.24 | 0.099962 | 1.053861 |
| TCGA-61-2098-01A | 0 | 0.005634 | 0.042972 | 0.054188 | 0 | 0.299703 | 0 | 0.035471 | 0.045079 | 0 | 0 | 0.147262 | 0 | 0.237724 | 0.003506 | 0.046053 | 0 | 0 | 0.058492 | 0 | 0.023917 | 0 | 0.39 | 0.039927 | 1.103338 |
| TCGA-61-2101-01A | 0.071312 | 0 | 0.005137 | 0.033886 | 0 | 0.197144 | 0 | 0.0008 | 0.028867 | 0 | 0 | 0.049475 | 0 | 0.051197 | 0.060051 | 0.384759 | 0.048876 | 0 | 0.068517 | 0 | 0 | 0 | 0.3 | 0.086129 | 1.059084 |
| TCGA-61-2102-01A | 0.004676 | 0 | 0.011612 | 0 | 0 | 0.145805 | 0 | 0.005803 | 0.069677 | 0 | 0 | 0.045153 | 0.01504 | 0.368061 | 0.005586 | 0.309563 | 0 | 0 | 0.019025 | 0 | 0 | 0 | 0.07 | 0.195807 | 1.075602 |
| TCGA-61-2104-01A | 0.055265 | 0 | 0 | 0.065111 | 0 | 0.129377 | 0 | 0.025554 | 0.058118 | 0 | 0 | 0.042898 | 0.0002 | 0.041025 | 0.125403 | 0.342742 | 0 | 0 | 0 | 0.089204 | 0 | 0.025087 | 0.03 | 0.232226 | 1.000394 |
| TCGA-61-2109-01A | 0 | 0.034027 | 0 | 0.046843 | 0 | 0.176597 | 0 | 0.012958 | 0.094077 | 0 | 0 | 0.099944 | 0 | 0.219863 | 0.009693 | 0.179896 | 0 | 0 | 0 | 0.081023 | 0 | 0.04508 | 0.3 | 0.090691 | 1.074986 |
| TCGA-61-2110-01A | 0 | 0.110521 | 0 | 0.041803 | 0 | 0.252927 | 0 | 0.006737 | 0.097533 | 0 | 0 | 0.099692 | 0.049572 | 0.057769 | 0.006623 | 0.228192 | 0 | 0 | 0 | 0.015908 | 0.013053 | 0.019669 | 0.3 | 0.08127 | 1.056902 |
| TCGA-61-2111-01A | 0.091204 | 0 | 0 | 0.082817 | 0 | 0.14475 | 0 | 0.001373 | 0.083477 | 0 | 0 | 0.056404 | 0.013721 | 0 | 0.028584 | 0.4248 | 0.040825 | 0 | 0.025196 | 0 | 0.001956 | 0.004894 | 0.09 | 0.178708 | 1.018602 |
| TCGA-61-2113-01A | 0.0009 | 0 | 0 | 0.055974 | 0 | 0.279693 | 0 | 0.040328 | 0.0182 | 0 | 0 | 0.025871 | 0.003107 | 0.076439 | 0.107548 | 0.251176 | 0 | 0.056016 | 0 | 0.049128 | 0.003553 | 0.032041 | 0.03 | 0.229275 | 1.004844 |
| TCGA-OY-A56Q-01A | 0.206343 | 0 | 0.032129 | 0.012156 | 0 | 0.19356 | 0 | 0.036339 | 0.019796 | 0 | 0 | 0.093799 | 0 | 0 | 0 | 0.207712 | 0 | 0.051318 | 0.070527 | 0 | 0 | 0.076323 | 0.74 | -0.0155 | 1.080786 |
| TCGA-VG-A8LO-01A | 0 | 0 | 0 | 0.072004 | 0 | 0.29642 | 0 | 0.122729 | 0.055785 | 0 | 0 | 0.17421 | 0 | 0 | 0.009348 | 0.045094 | 0 | 0.121616 | 0 | 0.051783 | 0.014605 | 0.036407 | 0.41 | 0.031705 | 1.116586 |
| TCGA-WR-A838-01A | 0 | 0 | 0.169419 | 0.008145 | 0 | 0.128431 | 0 | 0.152144 | 0.05863 | 0 | 0 | 0.081188 | 0 | 0.157309 | 0 | 0 | 0 | 0.2402 | 0 | 0 | 0.004534 | 0 | 0.32 | 0.064776 | 1.073223 |

**Supplementary Table 2. Pathway enrichment analyses (KEGG) of genes cluster A and B.**

| **Biological processes with significant enrichment in gene cluster A** | | | | | | | | |
| --- | --- | --- | --- | --- | --- | --- | --- | --- |
| ID | Description | GeneRatio | BgRatio | P value | p.adjust | q value | geneID | Count |
| hsa04060 | Cytokine-cytokine receptor interaction | 60/322 | 295/8111 | 3.76E-27 | 9.92E-25 | 7.36E-25 | 4283/2833/6352/10663/356/10563/3458/3561/6373/3627/3560/1234/959/6355/3594/3587/729230/1230/1236/3559/6348/6363/3601/608/6347/1439/3600/3586/5008/3459/1436/7040/3553/6357/3566/9235/3575/6367/3569/8764/6361/3570/8740/1524/6354/3597/3592/9180/3624/7293/6362/2920/2921/2919/6364/6372/8809/8792/3552/958 | 60 |
| hsa04061 | Viral protein interaction with cytokine and cytokine receptor | 36/322 | 100/8111 | 1.06E-25 | 1.40E-23 | 1.04E-23 | 4283/2833/6352/10563/3561/6373/3627/3560/1234/6355/3587/729230/1230/1236/3559/6348/6363/6347/3586/1436/6357/6367/3569/8764/6361/3570/8740/1524/6354/6362/2920/2921/2919/6364/6372/8809 | 36 |
| hsa04062 | Chemokine signaling pathway | 38/322 | 192/8111 | 6.25E-17 | 5.50E-15 | 4.08E-15 | 4283/2833/6352/10663/10563/6373/3627/3702/1234/6355/5880/729230/1230/1236/7454/6348/3055/6363/4067/6347/3717/2268/5294/6357/6367/5330/6361/1524/6354/6362/4792/2920/2921/2919/6364/115/6372/4790 | 38 |
| hsa05323 | Rheumatoid arthritis | 27/322 | 93/8111 | 9.12E-17 | 6.02E-15 | 4.47E-15 | 6352/3458/3683/942/941/6348/7097/3689/6347/3600/7040/3553/940/3569/1513/3383/54/2920/2921/2919/6364/2353/7010/6372/8792/3725/3552 | 27 |
| hsa04380 | Osteoclast differentiation | 29/322 | 128/8111 | 8.95E-15 | 4.73E-13 | 3.51E-13 | 3458/3937/7305/4689/10859/695/29760/11025/3459/1436/7040/10326/3553/8651/11027/11024/1540/1513/5468/54/4792/3716/2353/9021/3726/4790/8792/3725/3552 | 29 |
| hsa04668 | TNF signaling pathway | 27/322 | 112/8111 | 1.52E-14 | 6.68E-13 | 4.95E-13 | 6352/3627/3659/6347/3600/3553/3569/843/3383/840/330/7185/7128/4792/2920/2921/2919/6364/2353/9021/6372/3726/8809/4790/602/3725/4318 | 27 |
| hsa05144 | Malaria | 18/322 | 50/8111 | 2.13E-13 | 8.04E-12 | 5.97E-12 | 3458/3820/3683/959/7097/3689/6347/3586/7040/3553/3569/7057/6403/4615/3383/3592/3082/958 | 18 |
| hsa04064 | NF-kappa B signaling pathway | 24/322 | 104/8111 | 1.30E-12 | 4.30E-11 | 3.19E-11 | 23643/959/7535/695/6363/29760/4067/3553/6357/4615/8740/1540/3383/330/7185/7128/4792/2920/4616/2921/2919/4790/8792/958 | 24 |
| hsa05321 | Inflammatory bowel disease | 19/322 | 65/8111 | 3.29E-12 | 9.66E-11 | 7.16E-11 | 3458/3561/6775/3594/7097/3586/3459/7040/3553/3566/3569/3592/4094/2625/8809/4790/7100/3725/3552 | 19 |
| hsa05142 | Chagas disease | 23/322 | 102/8111 | 6.53E-12 | 1.72E-10 | 1.28E-10 | 915/916/6352/917/356/3458/6348/7097/6347/3586/3459/7040/3553/5330/2769/3569/4615/10333/3592/4792/2353/4790/3725 | 23 |
| hsa04620 | Toll-like receptor signaling pathway | 23/322 | 104/8111 | 1.00E-11 | 2.41E-10 | 1.79E-10 | 4283/6352/6373/3627/23643/942/941/6348/7097/7096/7098/3553/3569/4615/10333/1513/3592/4792/2353/4790/7100/3725/958 | 23 |
| hsa04659 | Th17 cell differentiation | 23/322 | 107/8111 | 1.88E-11 | 4.13E-10 | 3.06E-10 | 915/916/917/3458/3561/3560/7535/3594/3662/3559/3717/3459/7040/3553/3566/3569/3570/4792/2625/3716/2353/4790/3725 | 23 |
| hsa04658 | Th1 and Th2 cell differentiation | 21/322 | 92/8111 | 4.33E-11 | 8.79E-10 | 6.52E-10 | 915/916/917/3458/3561/3560/6775/7535/3594/3559/3717/3459/3566/3592/4094/4792/2625/3716/2353/4790/3725 | 21 |
| hsa05140 | Leishmaniasis | 19/322 | 77/8111 | 8.78E-11 | 1.66E-09 | 1.23E-09 | 3458/4689/1536/7097/3689/3717/3676/3586/3459/7040/3553/4615/3592/4792/3716/2353/4790/3725/3552 | 19 |
| hsa05162 | Measles | 25/322 | 139/8111 | 1.48E-10 | 2.61E-09 | 1.94E-09 | 915/916/917/356/3561/6504/3560/3559/7097/64135/4939/3553/4599/940/3569/4615/3592/4478/7128/4792/3716/2353/4790/3725/3552 | 25 |
| hsa05417 | Lipid and atherosclerosis | 31/322 | 215/8111 | 3.05E-10 | 5.03E-09 | 3.73E-09 | 6352/356/23643/959/4689/1536/6348/7097/4067/6347/3717/3553/5330/5452/3569/6403/4615/10333/3383/3592/840/5468/4792/2920/2921/2919/2353/4790/3725/4318/958 | 31 |
| hsa04514 | Cell adhesion molecules | 25/322 | 149/8111 | 6.91E-10 | 1.07E-08 | 7.95E-09 | 914/925/29851/6402/3683/5788/959/942/5133/3134/3385/3106/941/3689/923/3676/940/6403/3383/3685/3695/1272/3384/958/83700 | 25 |
| hsa05330 | Allograft rejection | 13/322 | 38/8111 | 1.06E-09 | 1.55E-08 | 1.15E-08 | 5551/3002/356/3458/959/942/3134/3106/941/3586/940/3592/958 | 13 |
| hsa04660 | T cell receptor signaling pathway | 20/322 | 104/8111 | 3.23E-09 | 4.40E-08 | 3.26E-08 | 915/916/925/917/3458/29851/3702/3937/9402/5788/959/5133/7535/3586/10125/940/4792/2353/4790/3725 | 20 |
| hsa04657 | IL-17 signaling pathway | 19/322 | 94/8111 | 3.37E-09 | 4.40E-08 | 3.26E-08 | 3458/3627/6347/3553/3569/6280/6361/6354/7128/4792/2920/2921/2919/6364/2353/6372/4790/3725/4318 | 19 |
| hsa04672 | Intestinal immune network for IgA production | 14/322 | 49/8111 | 3.50E-09 | 4.40E-08 | 3.26E-08 | 29851/959/942/941/3601/608/3600/3676/3586/7040/940/3569/3695/958 | 14 |
| hsa05332 | Graft-versus-host disease | 13/322 | 42/8111 | 4.31E-09 | 5.18E-08 | 3.84E-08 | 5551/3002/356/3458/942/3134/3106/3821/941/3553/940/3569/3552 | 13 |
| hsa04640 | Hematopoietic cell lineage | 19/322 | 99/8111 | 8.35E-09 | 9.59E-08 | 7.11E-08 | 915/914/916/925/924/917/952/921/951/3559/3676/1436/3553/3566/3575/3569/3570/7037/3552 | 19 |
| hsa05235 | PD-L1 expression and PD-1 checkpoint pathway in cancer | 18/322 | 89/8111 | 8.71E-09 | 9.59E-08 | 7.11E-08 | 915/916/917/3458/10538/5133/7535/7097/3717/3459/10125/940/4615/4792/3716/2353/4790/3725 | 18 |
| hsa05145 | Toxoplasmosis | 20/322 | 112/8111 | 1.23E-08 | 1.30E-07 | 9.66E-08 | 3458/1234/23643/959/3587/7097/3717/5294/3586/3459/7040/8651/240/4615/3592/330/4792/3716/4790/958 | 20 |
| hsa05340 | Primary immunodeficiency | 12/322 | 38/8111 | 1.34E-08 | 1.36E-07 | 1.01E-07 | 915/916/925/3561/29851/5788/959/7535/695/29760/3575/958 | 12 |
| hsa04630 | JAK-STAT signaling pathway | 24/322 | 162/8111 | 1.97E-08 | 1.93E-07 | 1.43E-07 | 3458/3561/3560/6775/3594/3587/3559/3601/1439/3600/3717/3586/5008/3459/8651/3566/3575/3569/3570/3597/3592/9180/3716/9021 | 24 |
| hsa04940 | Type I diabetes mellitus | 12/322 | 43/8111 | 6.33E-08 | 5.97E-07 | 4.42E-07 | 5551/3002/356/3458/942/3134/3106/941/3553/940/3592/3552 | 12 |
| hsa05152 | Tuberculosis | 24/322 | 180/8111 | 1.55E-07 | 1.41E-06 | 1.05E-06 | 3458/1520/3587/972/11151/7097/4046/3689/3717/7096/3586/3459/7040/3553/3687/3569/4615/10333/843/3592/3716/4790/1263/3552 | 24 |
| hsa05133 | Pertussis | 15/322 | 76/8111 | 2.18E-07 | 1.92E-06 | 1.43E-06 | 23643/3394/3659/3689/3586/3553/3569/4615/3592/840/2353/6372/4790/3725/3552 | 15 |
| hsa05134 | Legionellosis | 13/322 | 57/8111 | 2.41E-07 | 2.05E-06 | 1.52E-06 | 7097/3689/3553/3569/4615/3592/840/4792/2920/2921/2919/4790/7100 | 13 |
| hsa05166 | Human T-cell leukemia virus 1 infection | 26/322 | 219/8111 | 4.84E-07 | 3.99E-06 | 2.96E-06 | 915/916/917/3561/3560/3683/3134/3106/3559/3601/3689/3600/7040/3569/3383/8829/4792/3716/115/2353/2114/4790/7538/3725/1029/958 | 26 |
| hsa04662 | B cell receptor signaling pathway | 15/322 | 82/8111 | 6.17E-07 | 4.94E-06 | 3.66E-06 | 971/5880/10859/695/29760/4067/11025/11027/25780/11024/974/4792/2353/4790/3725 | 15 |
| hsa04621 | NOD-like receptor signaling pathway | 23/322 | 181/8111 | 6.80E-07 | 5.28E-06 | 3.92E-06 | 6352/9447/2633/1536/6347/4939/3553/3428/5330/9051/837/3569/4615/7226/330/7128/4792/2920/2921/3716/2919/4790/3725 | 23 |
| hsa05164 | Influenza A | 22/322 | 172/8111 | 1.07E-06 | 8.07E-06 | 5.98E-06 | 6352/356/3458/3627/6347/3717/64135/4939/91543/3459/7098/3553/4599/3569/4615/3383/3592/4792/3716/9021/4790/3552 | 22 |
| hsa05143 | African trypanosomiasis | 10/322 | 37/8111 | 1.13E-06 | 8.31E-06 | 6.16E-06 | 356/3458/8542/3586/3553/5330/3569/4615/3383/3592 | 10 |
| hsa05169 | Epstein-Barr virus infection | 24/322 | 202/8111 | 1.34E-06 | 9.53E-06 | 7.07E-06 | 915/916/917/3627/3683/3134/3106/695/7097/29760/4067/4939/3569/4615/3383/953/7128/4792/4616/3716/4790/3725/956/958 | 24 |
| hsa04210 | Apoptosis | 19/322 | 136/8111 | 1.54E-06 | 1.07E-05 | 7.96E-06 | 5551/3002/356/1521/1520/1439/1519/1512/843/1513/1522/840/330/7185/4792/4616/2353/4790/3725 | 19 |
| hsa05416 | Viral myocarditis | 12/322 | 60/8111 | 3.14E-06 | 2.13E-05 | 1.58E-05 | 5551/3683/959/942/3134/3106/5880/941/3689/940/3383/958 | 12 |
| hsa05320 | Autoimmune thyroid disease | 11/322 | 53/8111 | 5.56E-06 | 3.67E-05 | 2.72E-05 | 5551/3002/356/959/942/3134/3106/941/3586/940/958 | 11 |
| hsa05167 | Kaposi sarcoma-associated herpesvirus infection | 22/322 | 193/8111 | 7.38E-06 | 4.75E-05 | 3.52E-05 | 1234/942/3134/3106/1230/3055/4067/3717/5294/3459/7098/3569/3383/4792/2920/2921/3716/2919/2353/4790/7538/3725 | 22 |
| hsa04625 | C-type lectin receptor signaling pathway | 15/322 | 104/8111 | 1.34E-05 | 8.40E-05 | 6.23E-05 | 3659/4046/3586/3553/6367/3569/6361/1540/3592/4792/1960/4790/602/1263/3725 | 15 |
| hsa05146 | Amoebiasis | 14/322 | 102/8111 | 4.58E-05 | 0.000281 | 0.000208 | 3458/7097/3689/3586/7040/3553/5330/2769/3569/3592/2920/2921/2919/4790 | 14 |
| hsa04650 | Natural killer cell mediated cytotoxicity | 16/322 | 131/8111 | 5.73E-05 | 0.000344 | 0.000255 | 5551/3002/962/356/3458/3937/3683/7305/7535/3106/5880/3821/3689/3459/3383/3384 | 16 |
| hsa05135 | Yersinia infection | 16/322 | 137/8111 | 9.87E-05 | 0.000579 | 0.000429 | 925/3937/7535/5880/7454/6347/3676/3586/3553/3569/4615/4792/2353/4790/6197/3725 | 16 |
| hsa05171 | Coronavirus disease - COVID-19 | 22/322 | 232/8111 | 0.000128 | 0.000733 | 0.000543 | 3627/719/1536/7097/6347/64135/4939/7098/3553/4599/3569/6403/4615/3570/1839/3592/8829/4792/3716/2353/4790/3725 | 22 |
| hsa05202 | Transcriptional misregulation in cancer | 19/322 | 192/8111 | 0.000212 | 0.001192 | 0.000884 | 3002/3560/942/4005/1436/3087/3569/5468/3695/330/4094/7704/7185/4616/4300/860/4790/4318/958 | 19 |
| hsa05161 | Hepatitis B | 17/322 | 162/8111 | 0.000226 | 0.00122 | 0.000905 | 356/6775/7097/3717/64135/7098/7040/3569/4615/843/4792/3716/1960/2353/4790/3725/4318 | 17 |
| hsa05163 | Human cytomegalovirus infection | 21/322 | 225/8111 | 0.000227 | 0.00122 | 0.000905 | 6352/356/1234/3134/3106/5880/3587/1230/6348/6347/3553/5330/3569/3570/3685/4792/3716/5732/115/4790/1029 | 21 |
| hsa04010 | MAPK signaling pathway | 25/322 | 294/8111 | 0.000254 | 0.001342 | 0.000995 | 356/5880/5778/1436/7040/3553/10125/25780/11221/4615/51347/3082/8605/4616/1843/5801/2353/7010/1847/4790/7039/6197/1844/3725/3552 | 25 |
| hsa05120 | Epithelial cell signaling in Helicobacter pylori infection | 10/322 | 70/8111 | 0.000403 | 0.002085 | 0.001546 | 6352/4067/1839/4792/2920/2921/2919/4790/3725/83700 | 10 |
| hsa04933 | AGE-RAGE signaling pathway in diabetic complications | 12/322 | 100/8111 | 0.000567 | 0.002881 | 0.002137 | 1536/6347/3717/7040/3553/5330/3569/3383/2152/4790/3725/3552 | 12 |
| hsa04670 | Leukocyte transendothelial migration | 12/322 | 114/8111 | 0.001818 | 0.009054 | 0.006715 | 3702/399/3683/4689/5880/1536/3689/3676/3383/4478/4318/83700 | 12 |
| hsa05418 | Fluid shear stress and atherosclerosis | 13/322 | 139/8111 | 0.003436 | 0.0168 | 0.012459 | 3458/5880/6347/3162/3553/3383/3685/1843/2353/4790/3725/4318/3552 | 13 |
| hsa04217 | Necroptosis | 14/322 | 159/8111 | 0.004224 | 0.020275 | 0.015036 | 356/3458/6775/1536/3717/3459/7098/3553/1540/330/8605/7128/3716/3552 | 14 |
| hsa04664 | Fc epsilon RI signaling pathway | 8/322 | 68/8111 | 0.005247 | 0.024734 | 0.018343 | 3937/5880/695/241/4067/240/8605/2205 | 8 |
| hsa04917 | Prolactin signaling pathway | 8/322 | 70/8111 | 0.006258 | 0.028986 | 0.021497 | 3659/3717/8651/2353/9021/4790/8792/2099 | 8 |
| hsa04151 | PI3K-Akt signaling pathway | 24/322 | 354/8111 | 0.007106 | 0.032346 | 0.023989 | 356/3561/3560/3559/7097/3717/3676/5294/5008/1436/3566/3575/3569/7057/3570/3381/3685/3695/9180/3082/3716/7010/4790/7039 | 24 |
| hsa04145 | Phagosome | 13/322 | 152/8111 | 0.007302 | 0.032674 | 0.024232 | 1520/3134/4689/4481/3106/1536/11151/7097/3689/7057/10333/3685/7037 | 13 |
| hsa04611 | Platelet activation | 11/322 | 124/8111 | 0.010105 | 0.04446 | 0.032973 | 3937/695/4067/6916/5294/10125/5330/5023/5739/8605/115 | 11 |
| hsa04066 | HIF-1 signaling pathway | 10/322 | 109/8111 | 0.01111 | 0.048081 | 0.035658 | 3458/1536/3459/3162/3101/3569/3570/7037/7010/4790 | 10 |

| **Biological processes with significant enrichment in gene cluster B** | | | | | | | | |
| --- | --- | --- | --- | --- | --- | --- | --- | --- |
| ID | Description | GeneRatio | BgRatio | P value | p.adjust | q value | geneID | Count |
| hsa04510 | Focal adhesion | 35/524 | 201/8111 | 5.27E-08 | 1.64E-05 | 1.23E-05 | 1298/1288/10298/3911/2318/5296/7059/3265/3674/9475/5601/858/4233/824/3480/5295/3908/1793/3914/3696/83660/2317/3918/7450/1499/8515/5728/2932/7410/3479/55742/1399/1729/23396/5582 | 35 |
| hsa04710 | Circadian rhythm | 11/524 | 31/8111 | 1.90E-06 | 0.000297 | 0.000223 | 1454/5563/6095/23291/406/8864/8945/1408/4862/1385/5564 | 11 |
| hsa05205 | Proteoglycans in cancer | 31/524 | 205/8111 | 6.93E-06 | 0.00072 | 0.00054 | 3593/2260/3481/2318/5296/1975/3265/5335/287/7481/7475/6237/9475/4609/858/4233/23365/3480/5295/2017/3708/2317/1499/5781/3339/8324/6383/7410/3479/6469/5582 | 31 |
| hsa04360 | Axon guidance | 27/524 | 182/8111 | 3.84E-05 | 0.002996 | 0.002244 | 1949/10500/57556/10298/10505/5296/3265/5335/658/4690/1944/6237/5364/9475/4233/23365/5295/54434/1969/5362/5781/2050/2932/5590/8440/6469/3897 | 27 |
| hsa04152 | AMPK signaling pathway | 20/524 | 120/8111 | 7.53E-05 | 0.004701 | 0.003521 | 5213/5296/5210/32/5563/1994/3667/3480/1938/10645/5295/64764/5211/5208/890/1385/3479/5564/31/2194 | 20 |
| hsa04340 | Hedgehog signaling pathway | 12/524 | 56/8111 | 0.000189 | 0.009844 | 0.007373 | 1454/2736/11127/2737/23288/23432/50937/23291/8945/8452/2932/6469 | 12 |
| hsa04919 | Thyroid hormone signaling pathway | 19/524 | 121/8111 | 0.000254 | 0.011315 | 0.008475 | 4624/5213/477/482/5296/3265/5335/842/4854/4609/5295/5211/7067/2626/5208/1499/8648/2932/5582 | 19 |
| hsa04010 | MAPK signaling pathway | 35/524 | 294/8111 | 0.000301 | 0.011735 | 0.008789 | 2260/3481/5598/2318/2261/3265/782/23162/9448/1944/6237/4217/2248/5601/4609/7132/8986/3925/4233/773/11184/1649/3480/8912/9261/1969/6722/2768/2317/5495/1386/3479/1399/5494/5582 | 35 |
| hsa05010 | Alzheimer disease | 41/524 | 369/8111 | 0.000411 | 0.012853 | 0.009627 | 1454/5296/5685/322/3265/292/4040/7481/7416/842/7475/4711/4217/5601/7132/102/1649/4714/824/4717/5710/3667/5295/5692/823/1347/3708/4719/351/1499/4041/4726/317/8324/2932/293/1329/4842/6868/4707/324 | 41 |
| hsa04218 | Cellular senescence | 22/524 | 156/8111 | 0.000412 | 0.012853 | 0.009627 | 5296/3265/292/7416/6237/5883/4609/23291/5934/824/204851/9261/5295/823/2626/472/8945/3708/28996/890/5728/293 | 22 |
| hsa04211 | Longevity regulating pathway | 15/524 | 89/8111 | 0.000518 | 0.01387 | 0.010388 | 5296/3265/107/5563/109/3667/3480/10645/5295/64764/112/1386/1385/3479/5564 | 15 |
| hsa04520 | Adherens junction | 13/524 | 71/8111 | 0.000533 | 0.01387 | 0.010388 | 2260/1496/8936/4008/4233/5795/3480/8976/9855/10810/1499/117178/7082 | 13 |
| hsa04151 | PI3K-Akt signaling pathway | 39/524 | 354/8111 | 0.00067 | 0.016075 | 0.01204 | 1298/5585/2260/1288/2784/8115/3481/3911/5296/1975/2261/7059/3265/842/5563/1944/2248/3674/4609/4233/5586/5934/3667/3480/5295/3908/64764/1969/3914/3696/2786/3918/7450/8515/1386/5728/2932/1385/3479 | 39 |
| hsa05222 | Small cell lung cancer | 15/524 | 92/8111 | 0.000743 | 0.016555 | 0.0124 | 1288/3911/5296/842/7709/3674/4609/5295/3908/3914/1163/7187/3918/5728/317 | 15 |
| hsa04725 | Cholinergic synapse | 17/524 | 113/8111 | 0.000875 | 0.018206 | 0.013636 | 2784/3761/1137/5296/3265/107/773/3759/109/5295/64764/2786/3708/3784/112/1385/5582 | 17 |
| hsa04015 | Rap1 signaling pathway | 26/524 | 210/8111 | 0.00099 | 0.019307 | 0.014461 | 2260/5296/2261/3265/5335/107/1944/6237/2248/3674/4233/136/109/3480/5295/1969/83660/9855/112/1499/5590/7410/3479/1399/5582/26037 | 26 |
| hsa04210 | Apoptosis | 19/524 | 136/8111 | 0.001124 | 0.019477 | 0.014588 | 5366/8739/6709/5296/3265/142/842/1676/4217/5601/7132/1649/824/5295/823/472/3708/317/1677 | 19 |
| hsa05225 | Hepatocellular carcinoma | 22/524 | 168/8111 | 0.001141 | 0.019477 | 0.014588 | 3481/5296/3265/5335/4040/7481/7475/4609/4233/4780/3480/5295/1499/4041/2947/5728/8324/2932/6599/5582/2949/324 | 22 |
| hsa04928 | Parathyroid hormone synthesis, secretion and action | 16/524 | 106/8111 | 0.001186 | 0.019477 | 0.014588 | 2260/4040/5338/107/4205/109/64764/2768/3708/112/4041/11214/1386/1385/9826/5582 | 16 |
| hsa04730 | Long-term depression | 11/524 | 60/8111 | 0.001393 | 0.021737 | 0.016281 | 2781/3265/773/3480/2768/3708/1394/2977/3479/4842/5582 | 11 |
| hsa04935 | Growth hormone synthesis, secretion and action | 17/524 | 119/8111 | 0.001574 | 0.023382 | 0.017513 | 5296/3265/5335/107/5601/109/3667/5295/64764/3708/112/1386/2932/1385/3479/1399/5582 | 17 |
| hsa04810 | Regulation of actin cytoskeleton | 26/524 | 218/8111 | 0.001711 | 0.02427 | 0.018178 | 7114/2260/10152/50649/10298/5296/2261/3265/8936/6237/2248/3674/9475/23365/5295/54434/1793/8976/3696/2768/8515/7410/1399/1729/23396/324 | 26 |
| hsa04213 | Longevity regulating pathway - multiple species | 11/524 | 62/8111 | 0.001838 | 0.024938 | 0.018678 | 5296/3265/107/5563/109/3667/3480/5295/112/3479/5564 | 11 |
| hsa04550 | Signaling pathways regulating pluripotency of stem cells | 19/524 | 143/8111 | 0.002047 | 0.025104 | 0.018803 | 2260/2103/5296/2261/3265/7481/658/4211/7475/4609/3480/3625/5295/1499/8324/2932/3479/10336/324 | 19 |
| hsa05017 | Spinocerebellar ataxia | 19/524 | 143/8111 | 0.002047 | 0.025104 | 0.018803 | 5296/5685/292/4654/7416/4217/23369/5601/6095/773/5710/5295/5692/3708/9698/3752/6712/293/5582 | 19 |
| hsa04960 | Aldosterone-regulated sodium reabsorption | 8/524 | 37/8111 | 0.002092 | 0.025104 | 0.018803 | 477/482/5296/3758/3667/5295/3479/5582 | 8 |
| hsa04934 | Cushing syndrome | 20/524 | 155/8111 | 0.002253 | 0.026035 | 0.0195 | 6770/7481/107/1395/7475/5151/109/8912/64764/3708/1394/112/1499/1386/23746/8324/2932/1385/9070/324 | 20 |
| hsa04390 | Hippo signaling pathway | 20/524 | 157/8111 | 0.002625 | 0.029255 | 0.021911 | 1454/7159/2736/1496/7481/658/7475/4609/23291/10413/7004/8945/1499/8324/2932/5590/3993/654/3996/324 | 20 |
| hsa05224 | Breast cancer | 19/524 | 147/8111 | 0.002817 | 0.030307 | 0.022699 | 2260/5296/3265/4040/7481/7475/2248/4854/4609/3480/5295/1499/4041/8648/5728/8324/2932/3479/324 | 19 |
| hsa05022 | Pathways of neurodegeneration - multiple diseases | 46/524 | 475/8111 | 0.003414 | 0.035462 | 0.026561 | 1454/6804/1639/56171/10540/5685/3265/5335/292/23435/4040/7481/7416/842/10010/7475/4711/4217/5601/7132/1649/4714/824/4717/5710/5692/823/1347/3708/4719/351/1499/4041/4726/9927/317/8324/2932/65018/6712/293/1329/4842/4707/5582/324 | 46 |
| hsa05100 | Bacterial invasion of epithelial cells | 12/524 | 77/8111 | 0.003613 | 0.035462 | 0.026561 | 5296/1496/8936/858/4233/5295/1793/2017/8976/1499/1399/23607 | 12 |
| hsa05213 | Endometrial cancer | 10/524 | 58/8111 | 0.003637 | 0.035462 | 0.026561 | 5296/3265/1496/842/4609/5295/1499/5728/2932/324 | 10 |
| hsa04931 | Insulin resistance | 15/524 | 108/8111 | 0.003829 | 0.0355 | 0.026589 | 5296/32/5563/5601/7132/3667/5295/64764/5781/5728/2932/5836/5590/1385/5564 | 15 |
| hsa05231 | Choline metabolism in cancer | 14/524 | 98/8111 | 0.00396 | 0.0355 | 0.026589 | 5296/3265/10434/5335/5338/8936/1119/5601/6584/5295/10810/1609/23396/5582 | 14 |
| hsa04071 | Sphingolipid signaling pathway | 16/524 | 119/8111 | 0.003982 | 0.0355 | 0.026589 | 5296/3265/5338/56848/2206/4363/4217/9475/5601/7132/10558/5295/2768/5728/5590/5582 | 16 |
| hsa00051 | Fructose and mannose metabolism | 7/524 | 33/8111 | 0.004417 | 0.03828 | 0.028671 | 5213/5210/229/7264/231/5211/5208 | 7 |
| hsa05165 | Human papillomavirus infection | 34/524 | 331/8111 | 0.004602 | 0.038805 | 0.029065 | 1298/1288/3911/5296/7059/3265/7481/7475/3674/4854/7132/5934/9794/5295/3908/64764/3914/3696/472/7187/3918/7450/1499/8515/890/5728/8324/2932/535/5590/1385/3993/3996/324 | 34 |
| hsa05230 | Central carbon metabolism in cancer | 11/524 | 70/8111 | 0.004907 | 0.039785 | 0.029799 | 5213/2260/5296/2261/3265/4609/4233/3948/5295/5211/5728 | 11 |
| hsa04932 | Non-alcoholic fatty liver disease | 19/524 | 155/8111 | 0.005092 | 0.039785 | 0.029799 | 5296/5313/5563/4711/4217/5601/7132/1649/4714/4717/3667/5295/1347/4719/4726/2932/5564/1329/4707 | 19 |
| hsa00640 | Propanoate metabolism | 7/524 | 34/8111 | 0.005259 | 0.039785 | 0.029799 | 32/3948/5095/3030/1629/31/1738 | 7 |
| hsa04022 | cGMP-PKG signaling pathway | 20/524 | 167/8111 | 0.005352 | 0.039785 | 0.029799 | 4624/477/482/292/7416/107/9475/4205/109/3667/64764/2626/6722/2768/3708/112/2977/1386/1385/293 | 20 |
| hsa05163 | Human cytomegalovirus infection | 25/524 | 225/8111 | 0.005356 | 0.039785 | 0.029799 | 2784/5296/3265/107/842/9475/4609/7132/3588/23365/109/5295/64764/2786/2768/3708/112/1499/11214/1386/2932/1385/1399/9826/5582 | 25 |

**Supplementary Table 3. Gene Ontology (GO) enrichment of genes cluster A and B (TOP 50).**

**Biological processes with significant enrichment in gene cluster A**

| ID | Description | GeneRatio | BgRatio | pvalue | p.adjust | qvalue | geneID | Count |
| --- | --- | --- | --- | --- | --- | --- | --- | --- |
| GO:0042110 | T cell activation | 82/447 | 464/18670 | 1.30E-47 | 5.89E-44 | 3.81E-44 | 915/914/916/925/6352/924/917/3458/29851/3702/10875/399/4063/3683/10538/921/9402/5788/959/26191/942/199/2841/5133/7535/3594/3902/5880/3662/10859/972/729230/941/11151/1236/7454/3559/3659/6363/11326/4067/6347/3600/11314/923/634/5294/3586/91543/7040/10326/3553/8651/639/3566/5873/3575/10457/10125/11119/11221/940/3569/8764/3570/8740/1540/3383/3592/101/4478/9308/7704/8876/7293/2625/7037/678/1960/860/8809/602 | 82 |
| GO:0007159 | leukocyte cell-cell adhesion | 69/447 | 337/18670 | 2.30E-44 | 5.22E-41 | 3.38E-41 | 916/6352/3458/29851/10875/6402/3683/921/9402/5788/959/26191/942/199/5133/7535/3594/3902/5880/10859/972/729230/941/11151/1236/3559/3659/6363/11326/3689/4067/6347/3600/11314/923/3676/634/3586/7040/10326/3553/8651/3566/3575/10457/10125/11221/940/3569/6403/8764/6280/3570/8740/1524/1540/3383/3592/3695/101/4478/9308/7704/8876/4907/2625/7037/1960/9314 | 69 |
| GO:0050900 | leukocyte migration | 76/447 | 499/18670 | 2.60E-39 | 3.93E-36 | 2.54E-36 | 914/4283/2833/6352/962/10563/6373/3627/1234/6402/3683/6355/199/2841/7535/719/5880/972/729230/11151/1230/1236/6348/3055/6363/8832/3689/4067/6347/11314/3676/634/5294/3586/3162/7040/3553/6357/683/3687/1240/6367/9056/3569/7057/9023/6403/8764/6280/6361/3570/2683/8740/7226/1524/6354/3383/3592/3685/3695/101/4478/26585/6362/2920/2625/2921/1843/2919/6364/7010/6372/8792/6283/29/83700 | 76 |
| GO:1903037 | regulation of leukocyte cell-cell adhesion | 60/447 | 304/18670 | 1.34E-37 | 1.49E-34 | 9.64E-35 | 916/6352/3458/29851/10875/921/9402/5788/959/26191/942/199/5133/7535/3594/3902/10859/972/729230/941/11151/1236/3559/3659/6363/11326/3689/4067/6347/3600/11314/923/3676/634/3586/7040/10326/3553/8651/3566/3575/10457/10125/11221/940/3569/8764/3570/8740/1540/3383/3592/101/9308/7704/8876/2625/7037/1960/9314 | 60 |
| GO:0002237 | response to molecule of bacterial origin | 63/447 | 343/18670 | 1.64E-37 | 1.49E-34 | 9.64E-35 | 4283/6352/356/10225/10563/6373/3627/1234/23643/26191/9450/942/2672/3587/10859/941/968/3394/1236/6348/3055/7097/4067/6347/1439/3717/923/7096/3586/7040/3553/11027/11221/3569/6403/2643/1524/10333/5739/9516/3383/558/3592/54/8564/7128/10221/4792/2920/2921/8754/2919/5732/2353/6372/3726/4790/8792/5737/6197/7538/29/3725 | 63 |
| GO:0001819 | positive regulation of cytokine production | 71/447 | 464/18670 | 7.86E-37 | 5.85E-34 | 3.79E-34 | 914/916/3458/4063/23643/5788/959/9447/4332/26191/7305/942/199/3134/3594/3805/719/3662/10859/1536/972/729230/941/3394/1236/3659/6348/56253/6363/7097/3600/3717/923/7096/2268/64135/3586/5008/91543/3459/1436/7098/3162/7040/3553/11027/3566/3428/10125/940/3569/7057/8764/4615/3570/10333/1545/3592/101/330/23213/9308/3082/2625/150/8809/4790/7100/602/3552/958 | 71 |
| GO:0050863 | regulation of T cell activation | 60/447 | 314/18670 | 9.38E-37 | 5.85E-34 | 3.79E-34 | 914/916/6352/3458/29851/10875/921/9402/5788/959/26191/942/199/5133/7535/3594/3902/5880/3662/10859/972/729230/941/11151/1236/3559/3659/6363/11326/4067/6347/3600/11314/923/634/3586/7040/10326/3553/8651/639/3566/3575/10457/10125/11221/940/3569/8764/3570/8740/1540/3592/101/9308/7704/8876/2625/7037/1960 | 60 |
| GO:0050867 | positive regulation of cell activation | 66/447 | 394/18670 | 1.03E-36 | 5.85E-34 | 3.79E-34 | 914/916/6352/3458/29851/952/921/9402/5788/959/7305/942/199/5133/3134/7535/3594/5341/10859/972/729230/941/11151/695/1236/3559/6348/6363/3689/4067/6347/3600/3717/923/2268/3586/7040/10326/3553/8651/11027/3566/683/3575/10125/11221/940/3569/7057/6403/8764/3570/8740/10333/1540/558/3592/101/9308/7704/8876/7293/2625/7037/1960/958 | 66 |
| GO:0032496 | response to lipopolysaccharide | 61/447 | 330/18670 | 1.72E-36 | 8.40E-34 | 5.44E-34 | 4283/6352/356/10225/10563/6373/3627/1234/23643/26191/9450/942/2672/3587/10859/941/968/3394/1236/6348/3055/7097/4067/6347/1439/3717/923/3586/7040/3553/11027/11221/3569/6403/2643/1524/5739/9516/3383/558/3592/54/8564/7128/10221/4792/2920/2921/8754/2919/5732/2353/6372/3726/4790/8792/5737/6197/7538/29/3725 | 61 |
| GO:0051249 | regulation of lymphocyte activation | 72/447 | 485/18670 | 1.85E-36 | 8.40E-34 | 5.44E-34 | 914/916/6352/3458/29851/10875/952/921/9402/5788/959/4332/26191/7305/942/199/5133/3134/7535/3594/3902/5880/3662/10859/972/729230/941/11151/695/1236/3559/3659/6363/11326/4067/6347/3600/11314/923/634/3586/7040/10326/3553/8651/639/3566/683/3575/10457/10125/11221/940/3569/8764/3570/8740/1540/558/3592/101/9308/3624/7704/8876/7293/7128/2625/7037/678/1960/958 | 72 |
| GO:0060326 | cell chemotaxis | 58/447 | 304/18670 | 1.69E-35 | 6.98E-33 | 4.52E-33 | 4283/2833/6352/10663/10563/6373/3627/1234/6355/199/2841/719/5880/972/729230/11151/1230/1236/6348/6363/3689/4067/6347/5294/3586/3553/6357/683/1240/6367/3569/7057/9023/6280/6361/3570/8740/7226/1524/6354/4015/1839/3592/101/8829/3082/26585/6362/2920/2921/1843/2919/6364/1960/6372/8792/6283/83700 | 58 |
| GO:0030595 | leukocyte chemotaxis | 51/447 | 224/18670 | 3.01E-35 | 1.14E-32 | 7.36E-33 | 4283/2833/6352/10563/6373/3627/1234/6355/199/2841/719/5880/972/729230/11151/1230/1236/6348/6363/3689/4067/6347/5294/3586/3553/6357/683/1240/6367/3569/7057/9023/6280/6361/3570/8740/7226/6354/3592/101/26585/6362/2920/2921/1843/2919/6364/6372/8792/6283/83700 | 51 |
| GO:0030098 | lymphocyte differentiation | 61/447 | 353/18670 | 9.79E-35 | 3.19E-32 | 2.06E-32 | 915/914/916/925/917/3458/3702/10875/399/4063/10538/5788/959/26191/942/2841/7535/3594/3902/3662/972/729230/941/695/1236/3559/3659/6363/29760/3600/3676/3586/91543/7040/8651/639/3566/3575/10125/3087/974/11221/940/5452/3569/23228/1540/558/3592/101/9308/3624/7704/8876/1316/2625/678/1960/860/8809/602 | 61 |
| GO:1903039 | positive regulation of leukocyte cell-cell adhesion | 50/447 | 218/18670 | 9.84E-35 | 3.19E-32 | 2.06E-32 | 916/6352/3458/29851/921/9402/5788/959/942/199/5133/7535/3594/10859/972/729230/941/11151/1236/3559/6363/3689/4067/6347/3600/923/3676/7040/10326/3553/8651/3566/3575/10125/11221/940/3569/8764/3570/8740/1540/3383/3592/101/9308/7704/8876/2625/7037/1960 | 50 |
| GO:0022409 | positive regulation of cell-cell adhesion | 53/447 | 255/18670 | 1.87E-34 | 5.66E-32 | 3.66E-32 | 916/6352/10563/3458/29851/921/9402/5788/959/942/199/5133/7535/3594/10859/972/729230/941/11151/1236/3559/6363/3689/4067/6347/3600/923/3676/3586/7040/10326/3553/8651/3566/3575/10125/11221/940/3569/8764/3570/8740/1540/8728/3383/3592/101/9308/7704/8876/2625/7037/1960 | 53 |
| GO:0002696 | positive regulation of leukocyte activation | 62/447 | 380/18670 | 8.36E-34 | 2.37E-31 | 1.53E-31 | 914/916/6352/3458/29851/952/921/9402/5788/959/7305/942/199/5133/3134/7535/3594/10859/972/729230/941/11151/695/1236/3559/6348/6363/3689/4067/6347/3600/923/2268/3586/7040/10326/3553/8651/3566/683/3575/10125/11221/940/3569/7057/8764/3570/8740/10333/1540/558/3592/101/9308/7704/8876/7293/2625/7037/1960/958 | 62 |
| GO:0022407 | regulation of cell-cell adhesion | 63/447 | 402/18670 | 2.72E-33 | 7.25E-31 | 4.69E-31 | 916/6352/10563/3458/29851/10875/921/9402/5788/959/26191/942/199/5133/7535/3594/3902/10859/972/729230/941/11151/1236/3559/3659/6363/11326/3689/4067/6347/3600/11314/3717/923/3676/634/3586/7040/10326/3553/8651/3566/3575/10457/10125/11221/940/3569/8764/3570/8740/1540/8728/3383/3592/101/9308/7704/8876/2625/7037/1960/9314 | 63 |
| GO:0045785 | positive regulation of cell adhesion | 63/447 | 403/18670 | 3.15E-33 | 7.95E-31 | 5.14E-31 | 916/6352/10563/3458/29851/921/9402/5788/959/942/199/5133/7535/3594/10859/972/729230/941/11151/1236/3559/6363/3689/4067/6347/3600/3717/923/3676/3586/7040/10326/3553/8651/3566/3575/10125/11221/940/3569/8764/3570/8740/1540/8728/3383/3381/3592/3685/101/9308/8829/7704/8826/8876/2625/7037/8754/1960/7010/10085/7168/8646 | 63 |
| GO:0050870 | positive regulation of T cell activation | 47/447 | 202/18670 | 5.42E-33 | 1.29E-30 | 8.37E-31 | 916/6352/3458/29851/921/9402/5788/959/942/199/5133/7535/3594/10859/972/729230/941/11151/1236/3559/6363/4067/6347/3600/923/7040/10326/3553/8651/3566/3575/10125/11221/940/3569/8764/3570/8740/1540/3592/101/9308/7704/8876/2625/7037/1960 | 47 |
| GO:0030217 | T cell differentiation | 49/447 | 240/18670 | 1.64E-31 | 3.72E-29 | 2.41E-29 | 915/914/916/925/917/3458/3702/10875/399/4063/10538/5788/26191/942/2841/7535/3594/3902/3662/972/729230/941/1236/3559/3659/6363/3600/91543/7040/8651/639/3566/3575/10125/11221/940/3569/1540/3592/101/9308/7704/8876/2625/678/1960/860/8809/602 | 49 |
| GO:1902105 | regulation of leukocyte differentiation | 51/447 | 272/18670 | 6.97E-31 | 1.50E-28 | 9.73E-29 | 914/3458/10875/2124/5788/7305/942/7535/3594/3902/3662/10859/972/729230/941/695/1230/3559/3659/6348/6363/3059/4067/3600/634/11025/7098/7040/8651/639/3566/3575/10125/11221/940/1540/558/3592/101/9308/3624/7704/8876/10221/2625/678/1960/7071/2353/9770/3725 | 51 |
| GO:0051251 | positive regulation of lymphocyte activation | 55/447 | 334/18670 | 3.10E-30 | 6.39E-28 | 4.14E-28 | 916/6352/3458/29851/952/921/9402/5788/959/7305/942/199/5133/3134/7535/3594/10859/972/729230/941/11151/695/1236/3559/6363/4067/6347/3600/923/7040/10326/3553/8651/3566/683/3575/10125/11221/940/3569/8764/3570/8740/1540/558/3592/101/9308/7704/8876/7293/2625/7037/1960/958 | 55 |
| GO:0070661 | leukocyte proliferation | 51/447 | 298/18670 | 6.70E-29 | 1.32E-26 | 8.55E-27 | 916/6352/952/5788/959/4332/6355/26191/7305/942/199/7535/3594/5880/10859/972/729230/941/11151/695/3559/3659/6363/11326/4067/3600/11314/923/5294/3586/1436/7040/3553/683/3575/10457/3087/11119/940/3569/8764/23228/3570/8740/3592/4478/26585/7293/7128/7037/958 | 51 |
| GO:0097529 | myeloid leukocyte migration | 44/447 | 210/18670 | 7.14E-29 | 1.35E-26 | 8.73E-27 | 4283/6352/10563/6373/3627/6355/199/719/5880/972/729230/1230/1236/6348/6363/3689/4067/6347/11314/5294/3553/6357/683/1240/6367/3569/7057/6280/6361/3570/1524/6354/101/26585/6362/2920/2921/1843/2919/6364/6372/8792/6283/83700 | 44 |
| GO:0071219 | cellular response to molecule of bacterial origin | 44/447 | 212/18670 | 1.09E-28 | 1.98E-26 | 1.28E-26 | 4283/6352/10563/6373/3627/1234/23643/26191/9450/942/2672/10859/941/968/3394/6348/3055/7097/4067/6347/923/7096/3586/7040/3553/11027/3569/1524/10333/9516/3383/558/3592/8564/7128/10221/4792/2920/2921/8754/2919/6372/4790/7538 | 44 |
| GO:0070663 | regulation of leukocyte proliferation | 44/447 | 222/18670 | 8.43E-28 | 1.47E-25 | 9.51E-26 | 916/6352/952/5788/959/4332/6355/26191/7305/942/199/7535/3594/5880/10859/972/729230/941/11151/695/3559/3659/6363/11326/4067/3600/11314/923/3586/7040/3553/683/10457/3087/940/3569/8764/3570/3592/26585/7293/7128/7037/958 | 44 |
| GO:0071216 | cellular response to biotic stimulus | 45/447 | 236/18670 | 1.17E-27 | 1.96E-25 | 1.27E-25 | 4283/6352/10563/6373/3627/1234/23643/26191/9450/942/2672/10859/941/695/968/3394/6348/3055/7097/4067/6347/923/7096/3586/7040/3553/11027/3569/1524/10333/9516/3383/558/3592/8564/7128/10221/4792/2920/2921/8754/2919/6372/4790/7538 | 45 |
| GO:0071222 | cellular response to lipopolysaccharide | 42/447 | 205/18670 | 3.48E-27 | 5.64E-25 | 3.65E-25 | 4283/6352/10563/6373/3627/1234/23643/26191/9450/942/2672/10859/941/968/3394/6348/3055/7097/4067/6347/923/3586/7040/3553/11027/3569/1524/9516/3383/558/3592/8564/7128/10221/4792/2920/2921/8754/2919/6372/4790/7538 | 42 |
| GO:0002697 | regulation of immune effector process | 59/447 | 462/18670 | 2.52E-26 | 3.94E-24 | 2.55E-24 | 10225/3458/10875/5788/959/9447/7305/942/3134/3594/3106/3805/3902/719/5880/3662/10859/972/729230/941/695/7454/3559/56253/6363/11326/8832/3689/4067/3600/11314/2268/634/3586/91543/7098/3162/7040/3553/3566/3575/10125/3434/11221/940/3569/8764/3075/3383/3592/330/7293/7128/2625/7037/6372/8809/29/958 | 59 |
| GO:0002683 | negative regulation of immune system process | 57/447 | 435/18670 | 5.04E-26 | 7.61E-24 | 4.93E-24 | 29909/10225/10875/5788/4332/26191/7305/8530/942/2633/2841/5133/3134/3805/3902/10859/972/729230/941/695/968/3559/3659/6348/11326/8832/9404/4067/6347/11314/634/3586/11025/7098/3162/7040/8651/3566/25939/3575/10457/3428/11221/7057/8764/23228/558/5468/3624/7704/26585/7128/10221/4792/1843/7538/29 | 57 |
| GO:0046651 | lymphocyte proliferation | 46/447 | 272/18670 | 6.60E-26 | 9.66E-24 | 6.25E-24 | 916/6352/952/5788/959/4332/26191/7305/942/199/7535/3594/5880/10859/972/729230/941/11151/695/3559/3659/6363/11326/4067/3600/11314/923/5294/3586/7040/3553/683/3575/10457/11119/940/3569/8764/23228/3570/8740/3592/4478/7293/7037/958 | 46 |
| GO:0002685 | regulation of leukocyte migration | 40/447 | 196/18670 | 7.25E-26 | 1.03E-23 | 6.65E-24 | 2833/6352/10563/3627/6355/199/2841/719/5880/972/729230/1230/1236/6348/6363/4067/6347/11314/3676/3162/7040/683/1240/3569/7057/6403/8764/3570/8740/1524/6354/3383/3592/101/4478/26585/1843/6364/29/83700 | 40 |
| GO:0032943 | mononuclear cell proliferation | 46/447 | 274/18670 | 9.14E-26 | 1.26E-23 | 8.13E-24 | 916/6352/952/5788/959/4332/26191/7305/942/199/7535/3594/5880/10859/972/729230/941/11151/695/3559/3659/6363/11326/4067/3600/11314/923/5294/3586/7040/3553/683/3575/10457/11119/940/3569/8764/23228/3570/8740/3592/4478/7293/7037/958 | 46 |
| GO:1903706 | regulation of hemopoiesis | 58/447 | 475/18670 | 7.15E-25 | 9.54E-23 | 6.17E-23 | 914/29909/3458/10875/2124/5788/7305/942/7535/3594/3902/3662/10859/972/729230/941/695/1230/3559/3659/6348/6363/3059/4067/3600/4005/634/5008/11025/7098/7040/8651/639/3566/3575/10125/11221/940/7057/1540/4015/558/3592/101/9308/3624/7704/8876/10221/4792/2625/678/1960/7071/2353/7538/9770/3725 | 58 |
| GO:0050670 | regulation of lymphocyte proliferation | 40/447 | 208/18670 | 7.85E-25 | 1.02E-22 | 6.58E-23 | 916/6352/952/5788/959/4332/26191/7305/942/199/7535/3594/5880/10859/972/729230/941/11151/695/3559/3659/6363/11326/4067/3600/11314/923/3586/7040/3553/683/10457/940/3569/8764/3570/3592/7293/7037/958 | 40 |
| GO:0032944 | regulation of mononuclear cell proliferation | 40/447 | 209/18670 | 9.49E-25 | 1.20E-22 | 7.74E-23 | 916/6352/952/5788/959/4332/26191/7305/942/199/7535/3594/5880/10859/972/729230/941/11151/695/3559/3659/6363/11326/4067/3600/11314/923/3586/7040/3553/683/10457/940/3569/8764/3570/3592/7293/7037/958 | 40 |
| GO:0097530 | granulocyte migration | 34/447 | 141/18670 | 1.13E-24 | 1.38E-22 | 8.94E-23 | 4283/6352/10563/6373/3627/6355/719/5880/972/1236/6348/6363/3689/6347/11314/5294/3553/6357/683/1240/6367/7057/6280/6361/6354/101/6362/2920/2921/2919/6364/6372/6283/83700 | 34 |
| GO:0030593 | neutrophil chemotaxis | 30/447 | 104/18670 | 2.08E-24 | 2.43E-22 | 1.57E-22 | 4283/6352/10563/6373/3627/6355/719/5880/972/1236/6348/6363/3689/6347/5294/3553/6357/683/6367/6280/6361/6354/6362/2920/2921/2919/6364/6372/6283/83700 | 30 |
| GO:0071621 | granulocyte chemotaxis | 32/447 | 123/18670 | 2.09E-24 | 2.43E-22 | 1.57E-22 | 4283/6352/10563/6373/3627/6355/719/5880/972/1236/6348/6363/3689/6347/5294/3553/6357/683/1240/6367/7057/6280/6361/6354/6362/2920/2921/2919/6364/6372/6283/83700 | 32 |
| GO:1902107 | positive regulation of leukocyte differentiation | 34/447 | 144/18670 | 2.38E-24 | 2.70E-22 | 1.75E-22 | 3458/2124/5788/7305/942/7535/3594/972/941/695/1230/3559/6363/3059/3600/7040/8651/3566/3575/10125/11221/1540/558/3592/101/9308/7704/8876/10221/2625/1960/7071/2353/3725 | 34 |
| GO:0042035 | regulation of cytokine biosynthetic process | 31/447 | 114/18670 | 2.56E-24 | 2.83E-22 | 1.83E-22 | 916/3458/5788/7305/942/3902/3662/10859/1536/972/729230/941/3659/3717/7096/3586/7098/3162/3553/940/3569/7057/1524/10333/3624/2625/9314/4790/7538/602/3552 | 31 |
| GO:1990868 | response to chemokine | 29/447 | 97/18670 | 3.95E-24 | 4.17E-22 | 2.69E-22 | 4283/2833/6352/10663/10563/6373/3627/1234/6355/729230/1230/1236/6348/6363/6347/6357/1240/6367/6361/1524/6354/4015/6362/2920/2921/1843/2919/6364/6372 | 29 |
| GO:1990869 | cellular response to chemokine | 29/447 | 97/18670 | 3.95E-24 | 4.17E-22 | 2.69E-22 | 4283/2833/6352/10663/10563/6373/3627/1234/6355/729230/1230/1236/6348/6363/6347/6357/1240/6367/6361/1524/6354/4015/6362/2920/2921/1843/2919/6364/6372 | 29 |
| GO:0045619 | regulation of lymphocyte differentiation | 36/447 | 169/18670 | 4.64E-24 | 4.74E-22 | 3.07E-22 | 914/3458/10875/5788/942/7535/3594/3902/3662/972/729230/941/695/3559/3659/6363/3600/7040/8651/639/3566/3575/10125/11221/940/1540/558/3592/101/9308/3624/7704/8876/2625/678/1960 | 36 |
| GO:0002703 | regulation of leukocyte mediated immunity | 39/447 | 205/18670 | 4.70E-24 | 4.74E-22 | 3.07E-22 | 10225/5788/7305/3134/3594/3106/3805/3902/5880/10859/729230/695/7454/56253/8832/3689/4067/11314/2268/634/3586/91543/7098/3162/7040/3553/3566/3575/10125/940/3569/3383/3592/2625/7037/6372/8809/29/958 | 39 |
| GO:1990266 | neutrophil migration | 31/447 | 118/18670 | 8.08E-24 | 7.97E-22 | 5.15E-22 | 4283/6352/10563/6373/3627/6355/719/5880/972/1236/6348/6363/3689/6347/5294/3553/6357/683/6367/6280/6361/6354/101/6362/2920/2921/2919/6364/6372/6283/83700 | 31 |
| GO:0042089 | cytokine biosynthetic process | 31/447 | 123/18670 | 3.18E-23 | 3.07E-21 | 1.99E-21 | 916/3458/5788/7305/942/3902/3662/10859/1536/972/729230/941/3659/3717/7096/3586/7098/3162/3553/940/3569/7057/1524/10333/3624/2625/9314/4790/7538/602/3552 | 31 |
| GO:0042107 | cytokine metabolic process | 31/447 | 124/18670 | 4.15E-23 | 3.92E-21 | 2.54E-21 | 916/3458/5788/7305/942/3902/3662/10859/1536/972/729230/941/3659/3717/7096/3586/7098/3162/3553/940/3569/7057/1524/10333/3624/2625/9314/4790/7538/602/3552 | 31 |
| GO:0070098 | chemokine-mediated signaling pathway | 27/447 | 88/18670 | 6.94E-23 | 6.42E-21 | 4.15E-21 | 4283/2833/6352/10663/10563/6373/3627/1234/6355/729230/1230/1236/6348/6363/6347/6357/1240/6367/6361/1524/6354/6362/2920/2921/2919/6364/6372 | 27 |
| GO:0046631 | alpha-beta T cell activation | 32/447 | 138/18670 | 1.01E-22 | 9.18E-21 | 5.94E-21 | 916/3458/3702/4063/10538/5788/942/2841/7535/3594/3662/10859/729230/941/3659/6363/3600/11314/91543/8651/639/3566/940/3569/8764/3570/3592/9308/7704/2625/8809/602 | 32 |

**Biological processes with significant enrichment in gene cluster B**

| ID | Description | GeneRatio | BgRatio | pvalue | p.adjust | qvalue | geneID | Count |
| --- | --- | --- | --- | --- | --- | --- | --- | --- |
| GO:0007409 | axonogenesis | 53/873 | 468/18670 | 2.39E-09 | 7.07E-06 | 5.56E-06 | 4131/10494/11313/1949/10500/2736/6709/9024/5598/57556/10505/3975/11127/322/1496/5335/6711/1400/6812/107/2737/658/8153/1944/1000/5364/3925/23032/8301/9637/5295/3908/2017/1969/5362/10013/6722/1382/104/351/5781/5728/2050/3792/2932/6712/1385/26053/6469/5048/3897/26037/3996 | 53 |
| GO:0043254 | regulation of protein-containing complex assembly | 50/873 | 429/18670 | 2.60E-09 | 7.07E-06 | 5.56E-06 | 5366/6804/1639/7114/4131/10152/8115/8739/26146/6709/4926/1496/119/6711/11076/6812/142/8936/4690/830/5601/3925/4233/6653/10602/2017/5830/8976/7067/9886/10456/10013/472/822/9855/23221/5781/2932/1654/65018/5590/6712/1385/157922/55823/8440/5494/23521/1642/118 | 50 |
| GO:0072521 | purine-containing compound metabolic process | 51/873 | 464/18670 | 1.31E-08 | 2.38E-05 | 1.87E-05 | 5729/1633/7114/270/1622/5213/2103/2584/9759/10400/5313/1716/5210/107/142/32/56848/271/5563/229/6095/5151/2618/3948/136/316/109/9429/1969/5211/55746/5903/2646/4967/3614/5208/112/351/2977/54677/8573/10165/65018/3615/3479/22978/1329/31/1738/4842/2194 | 51 |
| GO:0019693 | ribose phosphate metabolic process | 46/873 | 424/18670 | 1.01E-07 | 0.000127 | 0.0001 | 7114/270/1622/5213/2103/2584/9759/5313/5210/107/142/32/56848/271/790/5563/229/6095/5151/2618/3948/109/1969/5211/55746/5903/2646/4967/3614/5208/112/351/2977/54677/8573/10165/22934/5836/65018/3615/3479/22978/1329/31/1738/2194 | 46 |
| GO:0019318 | hexose metabolic process | 32/873 | 244/18670 | 1.35E-07 | 0.000127 | 0.0001 | 5366/5213/2784/2103/2584/3481/216/5313/2822/5210/32/229/23038/6095/2820/231/3667/8864/5211/23275/5903/2646/4123/5208/4041/9563/10165/1609/2932/3479/2526/1642 | 32 |
| GO:0044262 | cellular carbohydrate metabolic process | 35/873 | 282/18670 | 1.40E-07 | 0.000127 | 0.0001 | 5366/2784/2103/2584/3481/9759/2822/5210/32/2137/10331/117/11181/6095/3269/2820/8871/231/3667/8864/9653/5903/2646/5208/8972/5728/1609/2932/5836/8867/2135/3479/3632/1642/5256 | 35 |
| GO:0006163 | purine nucleotide metabolic process | 46/873 | 432/18670 | 1.74E-07 | 0.000135 | 0.000106 | 7114/270/1622/5213/2103/2584/9759/5313/1716/5210/107/142/32/56848/271/5563/229/6095/5151/2618/3948/136/109/1969/5211/55746/5903/2646/4967/3614/5208/112/351/2977/54677/8573/10165/65018/3615/3479/22978/1329/31/1738/4842/2194 | 46 |
| GO:1901653 | cellular response to peptide | 42/873 | 385/18670 | 3.12E-07 | 0.000185 | 0.000145 | 4131/197/3481/5296/5313/6770/782/2822/107/142/4690/1395/1176/23038/9475/858/773/10768/231/4780/109/3667/3480/3625/6653/5295/1394/112/351/8462/890/5781/5728/2932/6599/535/3484/5590/1385/3479/9663/324 | 42 |
| GO:0009150 | purine ribonucleotide metabolic process | 43/873 | 399/18670 | 3.16E-07 | 0.000185 | 0.000145 | 7114/270/1622/5213/2103/2584/9759/5313/5210/107/142/32/56848/271/5563/229/6095/5151/2618/3948/109/1969/5211/55746/5903/2646/4967/3614/5208/112/351/2977/54677/8573/10165/65018/3615/3479/22978/1329/31/1738/2194 | 43 |
| GO:0009259 | ribonucleotide metabolic process | 44/873 | 414/18670 | 3.41E-07 | 0.000185 | 0.000145 | 7114/270/1622/5213/2103/2584/9759/5313/5210/107/142/32/56848/271/790/5563/229/6095/5151/2618/3948/109/1969/5211/55746/5903/2646/4967/3614/5208/112/351/2977/54677/8573/10165/65018/3615/3479/22978/1329/31/1738/2194 | 44 |
| GO:0032535 | regulation of cellular component size | 40/873 | 370/18670 | 7.51E-07 | 0.000371 | 0.000292 | 7114/4131/10152/2784/10500/6709/57556/10505/6344/1496/119/6711/8936/4690/830/6560/6557/8153/23038/6558/8301/10602/2017/8976/9886/10456/6722/822/1382/23221/5728/3792/2932/6712/1385/7410/8440/5048/3897/118 | 40 |
| GO:0071375 | cellular response to peptide hormone stimulus | 36/873 | 321/18670 | 1.13E-06 | 0.000511 | 0.000402 | 4131/197/3481/5296/5313/6770/2822/107/142/4690/1395/1176/23038/9475/858/10768/4780/109/3667/3480/3625/6653/5295/1394/112/890/5781/5728/2932/6599/535/3484/5590/1385/9663/324 | 36 |
| GO:0042752 | regulation of circadian rhythm | 19/873 | 114/18670 | 1.33E-06 | 0.000556 | 0.000438 | 1454/1815/2063/23435/107/5563/9475/5601/6095/23291/406/8864/8945/1408/2932/1385/7874/1642/5582 | 19 |
| GO:1902903 | regulation of supramolecular fiber organization | 38/873 | 353/18670 | 1.57E-06 | 0.00061 | 0.000479 | 1639/7114/4131/10152/10391/9639/6709/146057/4926/1496/119/6711/7481/22843/8936/4690/830/9475/3925/4233/5295/10602/2017/8976/9886/10456/4281/8500/10013/822/23221/351/11214/6712/157922/8440/118/324 | 38 |
| GO:0005996 | monosaccharide metabolic process | 33/873 | 287/18670 | 1.81E-06 | 0.000655 | 0.000515 | 5366/5213/2784/2103/2584/3481/216/5313/2822/5210/32/229/23038/6095/2820/231/3667/8864/5211/23275/5903/2646/4123/5208/4041/9563/10165/1609/22934/2932/3479/2526/1642 | 33 |
| GO:0016052 | carbohydrate catabolic process | 26/873 | 198/18670 | 1.94E-06 | 0.00066 | 0.000519 | 5213/2103/2584/9759/216/5313/5210/1486/5563/229/11181/2820/5211/55746/5903/2646/4967/4123/5208/351/4041/8972/5836/3479/2526/5256 | 26 |
| GO:0051656 | establishment of organelle localization | 41/873 | 401/18670 | 2.31E-06 | 0.000737 | 0.000579 | 1639/4131/10494/9696/10540/547/11235/11127/4926/1063/5108/6812/56848/8936/1176/10652/9525/2647/8301/136/8773/10128/10749/8976/23299/472/1394/1499/4134/8452/9632/65018/8943/5590/149111/8867/8729/4085/3993/5048/3996 | 41 |
| GO:0099003 | vesicle-mediated transport in synapse | 27/873 | 213/18670 | 2.47E-06 | 0.000746 | 0.000587 | 6804/1815/8927/9024/1137/6620/10675/6812/5338/107/1000/8618/8301/136/8773/321/6571/1499/22883/8573/5728/1609/2932/8943/8867/23396/5582 | 27 |
| GO:0051261 | protein depolymerization | 18/873 | 109/18670 | 2.88E-06 | 0.000817 | 0.000642 | 4131/6709/146057/119/6711/830/9525/3925/444/4281/10013/822/57553/6712/157922/8867/118/324 | 18 |
| GO:0018209 | peptidyl-serine modification | 35/873 | 322/18670 | 3.24E-06 | 0.000817 | 0.000642 | 1454/5585/9149/8115/9024/5598/11235/146057/142/4690/5563/8658/9475/5601/8986/7444/2589/11184/5586/204851/9261/2590/10456/10013/472/10769/351/28996/1609/2932/65018/5590/8440/4842/5582 | 35 |
| GO:0043434 | response to peptide hormone | 43/873 | 436/18670 | 3.36E-06 | 0.000817 | 0.000642 | 4131/197/3481/5296/7031/5313/4040/6770/2822/107/142/790/4690/1395/1176/23038/9475/858/10768/4780/109/3667/3480/3625/6653/5295/7049/3030/1394/112/890/5781/5728/2932/6599/535/3484/5590/1385/9663/8890/8614/324 | 43 |
| GO:0050804 | modulation of chemical synaptic transmission | 43/873 | 436/18670 | 3.36E-06 | 0.000817 | 0.000642 | 6804/4131/1815/477/1949/9746/2914/1137/6344/9145/3265/10675/5335/6770/6812/107/1395/1000/3269/7779/773/136/4355/3766/22941/54434/3908/10769/6722/321/1394/3749/351/22883/8573/5728/2932/65018/6146/5590/1385/5582/26037 | 43 |
| GO:0099177 | regulation of trans-synaptic signaling | 43/873 | 437/18670 | 3.56E-06 | 0.000817 | 0.000642 | 6804/4131/1815/477/1949/9746/2914/1137/6344/9145/3265/10675/5335/6770/6812/107/1395/1000/3269/7779/773/136/4355/3766/22941/54434/3908/10769/6722/321/1394/3749/351/22883/8573/5728/2932/65018/6146/5590/1385/5582/26037 | 43 |
| GO:0034329 | cell junction assembly | 41/873 | 409/18670 | 3.78E-06 | 0.000817 | 0.000642 | 421/4131/8927/10391/9746/3911/2318/8292/119/287/7481/1006/9448/8861/1000/5364/9475/9379/347902/5796/5586/5795/928/5318/22941/204851/2017/1969/3914/83660/6722/3918/351/1499/22883/5728/6712/1399/7082/23396/324 | 41 |
| GO:0032271 | regulation of protein polymerization | 27/873 | 218/18670 | 3.86E-06 | 0.000817 | 0.000642 | 1639/7114/4131/10152/6709/4926/1496/119/6711/11076/8936/4690/830/3925/4233/10602/2017/8976/9886/10456/822/23221/65018/6712/157922/8440/118 | 27 |
| GO:0099504 | synaptic vesicle cycle | 25/873 | 193/18670 | 3.91E-06 | 0.000817 | 0.000642 | 6804/8927/9024/1137/6620/10675/6812/5338/107/1000/8618/8301/136/8773/321/6571/1499/8573/5728/1609/2932/8943/8867/23396/5582 | 25 |
| GO:0060828 | regulation of canonical Wnt signaling pathway | 32/873 | 286/18670 | 4.62E-06 | 0.00093 | 0.000732 | 1454/8549/6659/5685/3485/4040/7481/2737/8658/5716/1000/4192/1649/5710/406/10413/9736/10076/5692/4163/8945/1499/8452/8324/2932/25805/1654/27130/3484/5494/6469/324 | 32 |
| GO:0046034 | ATP metabolic process | 33/873 | 304/18670 | 6.32E-06 | 0.001227 | 0.000965 | 7114/4624/5213/2103/477/2584/9759/5313/5210/142/56848/5563/229/4711/3948/4714/4717/5211/1347/55746/5903/2646/4967/5208/4719/351/4726/10165/65018/3479/1329/1738/4707 | 33 |
| GO:0060070 | canonical Wnt signaling pathway | 35/873 | 335/18670 | 7.83E-06 | 0.001466 | 0.001153 | 1454/4624/8549/6659/5685/3485/4040/7481/2737/8658/5716/1000/4192/1649/5710/406/10413/9736/10076/5692/4163/8945/1499/4041/8452/5728/8324/2932/25805/1654/27130/3484/5494/6469/324 | 35 |
| GO:0021695 | cerebellar cortex development | 11/873 | 47/18670 | 8.13E-06 | 0.001473 | 0.001158 | 2736/3975/146057/473/8861/6095/4192/23287/372/5781/6712 | 11 |
| GO:0062013 | positive regulation of small molecule metabolic process | 20/873 | 141/18670 | 9.13E-06 | 0.0016 | 0.001259 | 225/5366/7114/2103/3481/6770/2822/5210/5563/117/3269/136/3667/5208/351/65018/3479/1642/654/4842 | 20 |
| GO:1901880 | negative regulation of protein depolymerization | 13/873 | 66/18670 | 9.56E-06 | 0.001623 | 0.001276 | 4131/6709/146057/119/6711/830/4281/10013/822/6712/157922/118/324 | 13 |
| GO:0051258 | protein polymerization | 31/873 | 283/18670 | 9.98E-06 | 0.001631 | 0.001283 | 1639/7114/4131/10152/6709/4926/1496/119/6711/11076/8936/4690/830/11065/3925/4233/10602/2017/8976/9886/10456/822/10810/23221/10426/65018/6712/157922/8440/1729/118 | 31 |
| GO:0006836 | neurotransmitter transport | 30/873 | 270/18670 | 1.04E-05 | 0.001631 | 0.001283 | 6804/1815/477/2914/1137/2030/10675/6812/107/6538/8618/9379/7779/5799/136/3766/8773/8864/8500/321/6571/3749/8573/10165/6397/2932/8867/4842/23396/5582 | 30 |
| GO:0006006 | glucose metabolic process | 25/873 | 204/18670 | 1.05E-05 | 0.001631 | 0.001283 | 5366/5213/2784/2103/3481/5313/2822/32/229/23038/6095/2820/3667/8864/5211/5903/2646/5208/4041/9563/10165/1609/2932/3479/1642 | 25 |
| GO:0072522 | purine-containing compound biosynthetic process | 25/873 | 205/18670 | 1.14E-05 | 0.001709 | 0.001344 | 1633/7114/270/5313/1716/107/142/32/56848/271/2618/3948/136/109/3614/112/2977/10165/65018/3615/1329/31/1738/4842/2194 | 25 |
| GO:0018105 | peptidyl-serine phosphorylation | 32/873 | 299/18670 | 1.16E-05 | 0.001709 | 0.001344 | 1454/5585/9149/8115/9024/5598/11235/146057/4690/5563/8658/9475/5601/8986/7444/11184/5586/204851/9261/10456/10013/472/10769/351/28996/1609/2932/65018/5590/8440/4842/5582 | 32 |
| GO:0062012 | regulation of small molecule metabolic process | 41/873 | 429/18670 | 1.21E-05 | 0.001709 | 0.001344 | 225/5366/7114/2784/2103/3481/9759/4953/5685/6770/2822/5210/142/32/56848/5563/117/5716/23038/8720/6095/3269/1595/136/5710/3667/5692/55746/5903/2646/5208/351/1609/2932/65018/3479/31/1642/654/4842/2194 | 41 |
| GO:0031333 | negative regulation of protein-containing complex assembly | 19/873 | 132/18670 | 1.23E-05 | 0.001709 | 0.001344 | 7114/26146/6709/119/6711/6812/830/3925/6653/5830/7067/822/2932/1654/5590/6712/5494/23521/118 | 19 |
| GO:0032970 | regulation of actin filament-based process | 38/873 | 388/18670 | 1.45E-05 | 0.001965 | 0.001545 | 7114/10152/10391/477/9639/6709/3265/1496/119/6711/287/7481/22843/8936/4690/830/8153/9475/3925/4233/4192/3759/5318/5295/10602/2017/8976/9886/10456/8500/2626/822/23221/11214/6712/8440/23607/118 | 38 |
| GO:0045913 | positive regulation of carbohydrate metabolic process | 14/873 | 79/18670 | 1.57E-05 | 0.002085 | 0.001639 | 5366/2103/3481/2822/5210/5563/117/3269/3667/5903/5208/351/3479/1642 | 14 |
| GO:0030010 | establishment of cell polarity | 19/873 | 135/18670 | 1.70E-05 | 0.0022 | 0.00173 | 1639/4131/9024/4926/22841/2302/9475/51421/4134/2932/5590/8729/4085/1399/6469/3993/9826/5048/3996 | 19 |
| GO:0021549 | cerebellum development | 16/873 | 102/18670 | 1.98E-05 | 0.002504 | 0.001969 | 2736/3975/146057/473/4040/8861/3746/6095/4192/2618/23287/372/5781/8648/6712/1399 | 16 |
| GO:0098810 | neurotransmitter reuptake | 8/873 | 27/18670 | 2.22E-05 | 0.002742 | 0.002156 | 1815/477/2030/6538/3766/8864/6571/4842 | 8 |
| GO:0006090 | pyruvate metabolic process | 20/873 | 150/18670 | 2.31E-05 | 0.002794 | 0.002197 | 5213/4200/2103/2584/9759/5313/5210/7416/5563/229/3948/5211/55746/5903/2646/4967/5208/351/3479/1738 | 20 |
| GO:0031589 | cell-substrate adhesion | 35/873 | 354/18670 | 2.56E-05 | 0.003023 | 0.002377 | 10391/3911/7059/129080/10675/9448/8861/3674/9475/5796/4192/5795/5295/4289/1793/2017/3914/8500/6239/6722/3918/7450/1499/8515/3339/8573/5728/8324/2932/4585/5590/55742/1399/3897/50488 | 35 |
| GO:0001505 | regulation of neurotransmitter levels | 35/873 | 355/18670 | 2.72E-05 | 0.003059 | 0.002406 | 6804/1815/10003/477/2914/1137/8292/2030/10675/6812/107/6538/8618/9475/9379/1119/6095/7779/2618/5799/136/3766/8773/8864/6542/8500/321/6571/3749/8573/2932/8867/4842/23396/5582 | 35 |
| GO:1901879 | regulation of protein depolymerization | 14/873 | 83/18670 | 2.81E-05 | 0.003059 | 0.002406 | 4131/6709/146057/119/6711/830/444/4281/10013/822/6712/157922/118/324 | 14 |
| GO:0030902 | hindbrain development | 20/873 | 152/18670 | 2.81E-05 | 0.003059 | 0.002406 | 2736/3975/146057/1496/473/4040/8861/3746/6095/4192/2618/5362/23287/372/1499/5781/8648/6712/1399/6469 | 20 |
| GO:0032869 | cellular response to insulin stimulus | 25/873 | 216/18670 | 2.82E-05 | 0.003059 | 0.002406 | 197/3481/5296/5313/6770/2822/142/4690/1176/23038/858/3667/3480/3625/6653/5295/5781/5728/2932/6599/535/3484/5590/9663/324 | 25 |
