# Supplementary material for: Improving ovarian cancer treatment decision using a novel risk predictive tool
Source: Aging (Albany NY). 2022 Apr 19;14(8):3464–83. doi: 10.18632/aging.204023 (PMC9085236; doi:10.18632/aging.204023)
Supplement: Supplementary Tables 4 and 5 [file aging-14-204023-s003.pdf]

Supplementary Tables

Please browse Full Text version to see the data of Supplementary Tables 1–3.

Supplementary Table 1. The proportions of tumor microenvironment cells in 373 ovarian cancer patients.

Supplementary Table 2. Pathway enrichment analyses (KEGG) of genes cluster A and B.

Supplementary Table 3. Gene Ontology (GO) enrichment of genes cluster A and B (TOP 50).

Supplementary Table 4. The detailed medication information in different datasets.

The detailed medication information TCGA

Drug	Number of medication	Overall (333)
Platinum (Carboplatin, Cisplatin, Cisplatin+Gemcitabine, Oxaliplatin, Paclitaxel+Carboplatin, Topotecan+Carboplatin) (%)	0	8 (2.4)
	1	315 (94.6)
	2	10 (3.0)
Paclitaxel (%)	0	35 (10.5)
	1	267 (80.2)
	2	31 (9.3)
Others (%)	0	281 (84.4)
	1	51 (15.3)
	2	1 (0.3)
Unknown (%)	0	331 (99.4)
	1	2 (0.6)

The detailed medication information GSE30161

Drug	Overall (58)
Carboplatin	2 (3.4)
Carboplatin/Cytoxan	1 (1.7)
Carboplatin/Taxol	49 (84.5)
Cisplatin/Cytoxan	1 (1.7)
Cisplatin/Taxol	5 (8.6)

The detailed medication information GSE63885

Drug	Overall (75)
Platinum/Cyclophosphamide	34 (45.3)
Taxane/Platinum	41 (54.7)

The detailed medication information GSE23554

Drug	Overall (28)
Cisplatin	28 (100.0)

Supplementary Table 5. Summary of R package.

R package	Function
Affy	The package contains functions for Affymetrix GeneChip data analysis
clusterProfiler	Statistical analysis and visualization of functional profiles for genes and gene clusters
ComplexHeatmap	The package was used to visualize heatmap
ConsensusClusterPlus	The package was used to perform unsupervised clustering
estimate	The fraction of stromal and immune cells was calculated by estimate package
forestplot	The package was used to visualize forest plot
ggplot2	The package was used for data visualization
limma	Limma was used to identify the differentially expressed genes
maftools	The MAF files was analysed by maftools
NMF	Provides a framework to perform Non-negative Matrix Factorization
org.Hs.eg.db	Genome wide annotation for Human, primarily based on mapping using Entrez Gene identifiers.
psych	Principal component analysis was performed with psych
randomForest	The package was used to identify the differentially expressed genes
rms	The nomogram and calibration curve were generated with rms package
survival	Survival analysis was performed by survival package
survminer	Determine the optimal cutpoint for continuous variables and visualize the survival curves
sva	Removing batch effects in high-throughput experiment
tableone	The baseline patient characteristics was described by tableone
